# SBML level 3 package: spatial processes, version 1, release 1

**DOI:** 10.1515/jib-2022-0054

**Published:** 2023-03-10

**Authors:** James C. Schaff, Anuradha Lakshminarayana, Robert F. Murphy, Frank T. Bergmann, Akira Funahashi, Devin P. Sullivan, Lucian P. Smith

**Affiliations:** University of Connecticut, Storrs, CT, USA; Carnegie Mellon University, Pittsburgh, PA, USA; University of Heidelberg, Heidelberg, Germany; Keio University, Yokohama, Japan; KTH-Royal Institute of Technology, Stockholm, Sweden; University of Washington, Seattle, WA, USA

## Abstract

While many biological processes can be modeled by abstracting away the space in which those processes occur, some modeling (particularly at the cellular level) requires space itself to be modeled, with processes happening not in well-mixed compartments, but spatially-defined compartments. The *SBML Level 3 Core* specification does not include an explicit mechanism to encode geometries and spatial processes in a model, but it does provide a mechanism for SBML *packages* to extend the Core specification and add additional syntactic constructs. The SBML *Spatial Processes* package for SBML Level 3 adds the necessary features to allow models to encode geometries and other spatial information about the elements and processes it describes.

**James C. Schaff and Anuradha Lakshminarayana**, University of Connecticut, Storrs, CT, USA. https://orcid.org/0000-0003-3286-7736 (J. C. Schaff)

**Robert F. Murphy**, Carnegie Mellon University, Pittsburgh, PA, USA. https://orcid.org/0000-0003-0358-901X

**Frank T. Bergmann**, University of Heidelberg, Heidelberg, Germany. https://orcid.org/0000-0001-5553-4702

**Akira Funahashi**, Keio University, Yokohama, Japan. https://orcid.org/0000-0003-0605-239X

SBML Level 3 Package Specification

## Spatial Processes

James C. Schaff
schaff@uchc.edu
Center for Cell Analysis and Modeling
University of Connecticut, US

Anuradha Lakshminarayana
alak04@gmail.com
Center for Cell Analysis and Modeling
University of Connecticut, US

Robert F. Murphy
murphy@cmu.edu
Computational Biology Department
Carnegie Mellon University, US

Frank T. Bergmann
frank.bergmann@bioquant.uni-heidelberg.de
BioQuant/Centre for Organismal Studies (COS)
University of Heidelberg, Germany

Akira Funahashi
funa@bio.keio.ac.jp
Dept. of Biosciences and Informatics
Keio University, Japan

Devin P. Sullivan
devsullivan@gmail.com
Science for Life Laboratory
KTH-Royal Institute of Technology, Sweden*
*(Currently at Encodia, US)

Lucian P. Smith
lpsmith@u.washington.edu
Department of Bioengineering
University of Washington, US

Version 1, Release 1

October 27, 2022

The latest release, past releases, and other materials related to this specification are available at
https://sbml.org/documents/specifications/level-3/version-1/spatial/

*This* release of the specification is available at
https://co.mbine.org/specifications/sbml.level-3.version-1.spatial.version-1.release-1

## Contents

1 Introduction 5
1.1 Proposal corresponding to this package specification 6
1.2 Package dependencies 6
1.3 Document conventions 6
2 Background and context 7
2.1 Problems with current SBML approaches 7
2.2 Past work on this problem or similar topics 7
3 Package syntax and semantics 8
3.1 Overview of spatial extension 8
3.1.1 Geometry 8
3.1.2 Alternative ***GeometryDefinitions*** 8
3.2 Namespace URI and other declarations necessary for using this package 8
3.3 Primitive data types 9
3.3.1 Type `SpId` 9
3.3.2 Type `SpIdRef` 9
3.3.3 Type `BoundaryConditionKind` 9
3.3.4 Type `CoordinateKind` 9
3.3.5 Type `DataKind` 9
3.3.6 Type `DiffusionKind` 10
3.3.7 Type `CompressionKind` 10
3.3.8 Type `FunctionKind` 10
3.3.9 Type `GeometryKind` 10
3.3.10 Type `InterpolationKind` 10
3.3.11 Type `PolygonKind` 10
3.3.12 Type `PrimitiveKind` 10
3.3.13 Type `SetOperation` 11
3.3.14 Type `doubleArray` 11
3.3.15 Type `arrayData` 11
3.4 The extended **Model** object 11
3.5 The extended **Compartment** object 11
3.6 The **CompartmentMapping** class 12
3.6.1 The `id` and `name` attributes 12
3.6.2 The `domainType` attribute 12
3.6.3 The `unitSize` attribute 12
3.7 The extended **Species** object 13
3.7.1 The `isSpatial` attribute 13
3.8 The extended **Parameter** object 14
3.9 The **SpatialSymbolReference** class 14
3.9.1 The `spatialRef` attribute 15
3.10 The **DiffusionCoefficient** class 15
3.10.1 The `variable` attribute 15
3.10.2 The `type` and `coordinateReference` attributes 15
3.10.3 **DiffusionCoefficient** uniqueness 15
3.11 The **AdvectionCoefficient** class 16
3.11.1 The `variable` attribute 16
3.11.2 The `coordinate` attribute 16
3.12 The **BoundaryCondition** class 16
3.12.1 The `variable` attribute 17
3.12.2 The `type` attribute 17
3.12.3 The `coordinateBoundary` attribute 17
3.12.4 The `boundaryDomainType` attribute 17
3.13 Extended **Parameter** examples 17
3.14 The extended **Reaction** object 18
3.14.1 The `isLocal` attribute 18
3.15 The **CoordinateComponent** class 18
3.15.1 The `id` and `name` attributes 19
3.15.2 The `type` attribute 19
3.15.3 The `unit` attribute 19
3.16 The **SampledField** class 20
3.16.1 The `id` and `name` attributes 20

3.16.2 The interpolationType attribute . . . 21
3.16.3 The numSamples1, numSamples2, numSamples3 attributes . . . 21
3.16.4 The compression attribute . . . 21
3.16.5 The samplesLength attribute . . . 21
3.16.6 The dataType attribute . . . 21
3.16.7 The Samples text child . . . 22
3.16.8 A **SampledField** example . . . 22
3.17 The **Geometry** class . . . 22
3.17.1 The id and name attributes . . . 23
3.17.2 The coordinateSystem attribute . . . 24
3.17.3 The listOf container classes . . . 24
3.18 The **Boundary** class . . . 24
3.18.1 The id and name attributes . . . 24
3.18.2 The value attribute . . . 24
3.18.3 A **CoordinateComponent** example . . . 25
3.19 The **DomainType** class . . . 25
3.19.1 The id and name attributes . . . 25
3.19.2 The spatialDimensions attribute . . . 26
3.20 The **Domain** class . . . 26
3.20.1 The id and name attributes . . . 26
3.20.2 The domainType attribute . . . 27
3.21 The **InteriorPoint** class . . . 27
3.21.1 The coord1, coord2, and coord3 attributes . . . 27
3.22 The **AdjacentDomains** class . . . 27
3.22.1 The id and name attributes . . . 27
3.22.2 The domain1 and domain2 attributes . . . 27
3.23 Domain example . . . 28
3.24 The **GeometryDefinition** class . . . 28
3.24.1 The id and name attributes . . . 28
3.24.2 The isActive attribute . . . 29
3.25 The **AnalyticGeometry** class . . . 29
3.26 The **AnalyticVolume** class . . . 29
3.26.1 The id and name attributes . . . 29
3.26.2 The functionType attribute . . . 29
3.26.3 The domainType attribute . . . 30
3.26.4 The ordinal attribute . . . 30
3.27 The **Math** class . . . 31
3.28 **AnalyticGeometry** example . . . 31
3.29 The **SampledFieldGeometry** class . . . 31
3.29.1 The sampledField attribute . . . 31
3.30 The **SampledVolume** class . . . 31
3.30.1 The id and name attributes . . . 31
3.30.2 The domainType attribute . . . 32
3.30.3 The sampledValue attribute . . . 32
3.30.4 The minValue attribute . . . 32
3.30.5 The maxValue attribute . . . 32
3.31 **SampledFieldGeometry** example . . . 33
3.32 The **CSGeometry** class . . . 33
3.33 The **CSGObject** class . . . 33
3.33.1 The id and name attributes . . . 33
3.33.2 The domainType attribute . . . 34
3.33.3 The ordinal attribute . . . 34
3.33.4 The [csgNode] child . . . 34
3.34 The **CSGNode** class . . . 34
3.34.1 The id and name attributes . . . 35
3.35 The **CSGPrimitive** class . . . 35
3.35.1 The primitiveType attribute . . . 35
3.36 The **CSGSetOperator** class . . . 35
3.36.1 The operationType attribute . . . 36
3.36.2 The complementA and complementB attributes . . . 36
3.37 The **ListOfCSGNodes** class . . . 36
3.38 The **CSGTransformation** class . . . 36

3.38.1 The csgNode child . . . 37
3.39 The **CSGTranslation** class . . . 37
3.39.1 The translateX attribute . . . 37
3.39.2 The translateY attribute . . . 38
3.39.3 The translateZ attribute . . . 38
3.40 The **CSGRotation** class . . . 38
3.40.1 The rotateX, rotateY, and rotateZ attributes . . . 38
3.40.2 The rotateAngleInRadians attribute . . . 38
3.41 The **CSGScale** class . . . 38
3.41.1 The scaleX attribute . . . 38
3.41.2 The scaleY attribute . . . 39
3.41.3 The scaleZ attribute . . . 39
3.42 The **CSGHomogeneousTransformation** class . . . 39
3.43 The **TransformationComponent** class . . . 39
3.43.1 The components attribute . . . 39
3.43.2 The componentsLength attribute . . . 39
3.44 CSGObject examples . . . 39
3.45 The **ParametricGeometry** class . . . 40
3.46 The **SpatialPoints** class . . . 40
3.46.1 The compression attribute . . . 40
3.46.2 The arrayDataLength attribute . . . 40
3.46.3 The dataType attribute . . . 40
3.46.4 The ArrayData text child . . . 41
3.46.5 The id and name attributes . . . 42
3.47 The **ParametricObject** class . . . 42
3.47.1 The id and name attributes . . . 42
3.47.2 The polygonType attribute . . . 42
3.47.3 The domainType attribute . . . 42
3.47.4 The compression attribute . . . 42
3.47.5 The pointIndexLength attribute . . . 42
3.47.6 The dataType attribute . . . 42
3.47.7 The PointIndex text child . . . 43
3.48 A **ParametricGeometry** example . . . 43
3.49 The **MixedGeometry** class . . . 44
3.50 The **OrdinalMapping** class . . . 45
3.50.1 The geometryDefinition attribute . . . 45
3.50.2 The ordinal attribute . . . 45
**4 Examples** **46**
**5 Interaction with other packages** **47**
5.1 SBML Level 3 Version 2 . . . 47
5.2 The comp package . . . 47
5.3 The distrib package . . . 47
5.4 The multi package . . . 47
5.5 The qual package . . . 47
5.6 The layout and render packages . . . 48
**A Validation of SBML documents** **49**
A.1 Validation and consistency rules . . . 49
**Acknowledgments** **78**
**References** **79**

## 1 Introduction

There is no better test of our understanding of a biological system than to create a mechanistic model of that system and show that it possesses all known properties of the system – i.e., that it is consistent with all measurements or observations of the system in hand. However, the creation of such models is even more useful when a system is not already understood, because the model may suggest mechanisms by which known behaviors are accomplished, or predict behaviors not previously expected. Extensive work has been done on the creation of methods for simulating biochemical systems (Andrews and Bray, 2004; Blinov et al., 2004; Hoops et al., 2006; Loew and Schaff, 2001; Stiles et al., 1998; Tomita et al., 1999), including the development of languages, such as the Systems Biology Markup Language (SBML), for saving and exchanging the definition of the system being simulated and the simulation parameters used (Hucka et al., 2003; Keating et al., 2020). Historically, most tools for biochemical simulations either did not consider possible spatial relationships or compartmentalization of system components, or used a compartmental modeling approach (Jacquez et al., 1985) in which spatial organization is approximated by a set of compartments (e.g. membrane-bound organelles in a cell) containing well-mixed populations of molecules. However, a growing number of modeling and simulation tools do permit specification of the size, shape and positions of compartments and the initial spatial distributions of components (typically referred to as a 'geometry' definition). While SBML Level 3 Core has explicit support for multi-compartmental modeling, it does not have a standardized mechanism to store or exchange geometries. We address this deficit here through the definition of a package of extensions to SBML termed SBML spatial.

Saving geometries in a standardized way is useful to ensure that the definition is complete (which is important when, for example, provided as part of supplementary information for published studies), and to permit comparison of simulations of the same spatial system using different simulation tools, to enable simulations of a new biochemical model using the same geometry as a previous model (or vice versa). Historically, the creation of a geometry has been done (often through painstaking manual work) by the same investigators who defined a biochemical model and performed a simulation. The advent of SBML has permitted a partial division of this labor, by enabling databases of biochemical models, such as the BioModels database (Le Novere et al., 2006; Li et al., 2010; Malik-Sheriff et al., 2020), to be created. Building on this theme, having a mechanism to exchange geometries will enable an open access 'marketplace' in which some investigators focus on the creation of well-characterized geometries (and distribute them through databases or repositories) enabling others to focus on biochemical model creation and/or performance of simulations. These geometries, however, typically need to be more than raw images. To be most broadly useful across different modeling approaches, they need, for example, to define explicit watertight boundaries for cells and intracellular compartments and contain statistically accurate estimates of concentrations of components at various locations. This can be accomplished for individual images by hand segmentation (Loew and Schaff, 2001) or automated segmentation (Perez et al., 2014). An alternative is to produce synthetic geometries that are drawn from generative models of cell shape and organization learned from many images (Zhao and Murphy, 2007). In both cases, the availability of well-characterized geometries for examples of different cell types (and multiple cells of each type) can enhance the use of simulation methods.

Examples of simulations using biochemical models and geometries from different sources have already occurred using a preliminary version of the SBML spatial standard (Donovan et al., 2016; Sullivan et al., 2015). In these, cell geometries created using CellOrganizer and saved using the SBML spatial extension were combined with biochemical models created using BioNetGen and saved in SBML.

Additionally, properties of molecules or reactions may vary spatially, and rates of transport between locations need to be specified.

In summary, the purpose of this SBML Level 3 extension is to meet the needs identified above by defining standardized ways of representing geometries, spatial mappings of species and reactions, and explicit species transport.

### 1.1 Proposal corresponding to this package specification

This specification for Spatial in SBML Level 3 Version 1 is available at the following URL:

https://github.com/sbmlteam/sbml-specifications/tree/release/sbml-level-3/version-1/spatial/specification

The Package Working Group for the Spatial package is coordinated on the mailing list https://lists.sourceforge.net/lists/listinfo/sbml-spatial. More information about the spatial package is available at http://sbml.org/Documents/Specifications/SBML_Level_3/Packages/spatial.

### 1.2 Package dependencies

The Spatial package has no dependencies on other SBML Level 3 packages.

### 1.3 Document conventions

UML 1.0 (Unified Modeling Language; Eriksson and Penker 1998; Oestereich 1999) notation is used in this document to define the constructs provided by this package. Colors in the diagrams carry the following additional information for the benefit of those viewing the document on media that can display color:

- *Black*: Items colored black in the UML diagrams are components taken unchanged from their definition in the SBML Level 3 Core specification document.
- *Green*: Items colored green are components that exist in SBML Level 3 Core, but are extended by this package. Class boxes are also drawn with dashed lines to further distinguish them.
- *Blue*: Items colored blue are new components introduced in this package specification. They have no equivalent in the SBML Level 3 Core specification.
- *Red lines*: Classes with red lines in the corner are fully defined in a different figure.

The following typographical conventions distinguish the names of objects and data types from other entities; these conventions are identical to the conventions used in the SBML Level 3 Core specification document:

***AbstractClass***: Abstract classes are never instantiated directly, but rather serve as parents of other classes. Their names begin with a capital letter and they are printed in a slanted, bold, sans-serif typeface. In electronic document formats, the class names defined within this document are also hyperlinked to their definitions; clicking on these items will, given appropriate software, switch the view to the section in this document containing the definition of that class. (However, for classes that are unchanged from their definitions in SBML Level 3 Core, the class names are not hyperlinked because they are not defined within this document.)

**Class**: Names of ordinary (concrete) classes begin with a capital letter and are printed in an upright, bold, sans-serif typeface. In electronic document formats, the class names are also hyperlinked to their definitions in this specification document. (However, as in the previous case, class names are not hyperlinked if they are for classes that are unchanged from their definitions in the SBML Level 3 Core specification.)

`SomeThing`, `otherThing`: Attributes of classes, data type names, literal XML, and tokens *other* than SBML class names, are printed in an upright typewriter typeface.

`[elementName]`: In some cases, an element may contain a child of any class inheriting from an abstract base class. In this case, the name of the element is indicated by giving the abstract base class name in brackets, meaning that the actual name of the element is the de-capitalized form of whichever subclass is used.

For other matters involving the use of UML and XML, this document follows the conventions used in the SBML Level 3 Core specification document.

## 2 Background and context

### 2.1 Problems with current SBML approaches

There is no standard way of specifying spatial models in SBML short of introducing an explicit spatial discretization in the form of a large number of compartments with duplicate species and reaction information, with additional reactions for coupling due to transport. This approach hard-codes the numerical methods and discretization schema which destroys portability and is not practical beyond a few compartments. Tools have been forced to resort to proprietary extensions (e.g. MesoRD custom annotations) to encode geometry.

### 2.2 Past work on this problem or similar topics

There are many standards for the exchange of geometric information of engineered parts in Computer Aided Design and Manufacturing. These formats are designed for geometric shapes which are directly specified by a designer rather than the data driven, freeform biological structures encountered in cell biology.

There also exist standards for the representation of unstructured computational meshes that can encode these freeform biological structures more readily. However, it is important to note that while a computational mesh necessarily encodes an approximation to the shapes of geometric objects, the particular form will be algorithm dependent.

To ensure model interoperability, we must encode the geometric shapes in a way that is independent of the numerical methods and even the mathematical framework. The representation of a spatial model within SBML should be largely invariant of the particular encoding of the geometry definition within that model. For example, a spatial model represented in SBML that encodes geometry as a set of geometric primitives (e.g. spheres, cylinders) should be easily portable to a tool that only supports polygonal surface tessellation. It is expected that a geometry translation library will be very useful for interoperability the same way that libSBML greatly improved model interchange by solving similar implementation problems in a standard way.

## 3 Package syntax and semantics

This section contains a definition of the syntax and semantics of the Spatial package for SBML Level 3 Version 1 Core. The Spatial package involves several new object classes, and extends the existing **Model**, **Compartment**, **Species**, **Reaction**, and **Parameter** object class. Section 4 on page 46 contains complete examples of using the constructs in SBML models.

### 3.1 Overview of spatial extension

The SBML **Compartment**, **Reaction** and **Species**, and molecular transport mechanisms (**DiffusionCoefficient**, **AdvectionCoefficient**, **BoundaryCondition**) are mapped to geometric domains to describe spatial models within SBML. The primary mechanism to accomplish this mapping is to simply map **Compartments** to collections of geometric Domains called **DomainTypes**. Each **Domain** is a contiguous patch of volumetric space or a contiguous surface patch that is ultimately described by a single system of equations (whichever mathematical framework is used). In analogy with initial conditions, the mathematical system defined within a domain often needs a definition of what happens at the domain boundary (e.g. boundary conditions) to complete the specification. Because of this, the boundaries between adjacent domains need to be identified so that appropriate boundary conditions can be specified. For compactness of representation, rather than map to each individual **Domain**, **Compartments** are mapped to **DomainTypes**, along with the corresponding **Species** and **Reactions** (with the new compartment attribute).

#### 3.1.1 Geometry

The **Geometry** object within a model is completely modular and does not reference the rest of the model, promoting reuse of the same geometry in different models. The geometry separately defines a coordinate system, a list of domain types, a list of domains and their adjacency relationships, and a list of alternate geometric representations.

#### 3.1.2 Alternative *GeometryDefinitions*

Modeling and simulation tools will each natively support some subset (often just one) of the possible ***GeometryDefinitions*** (analytic, sampled field, constructive solid geometry, parametric, or mixed shapes). Interoperability will be enhanced if tools write as many geometry definitions as they are able. Upon reading the model, a tool will typically choose the most convenient geometry definition, i.e. the one that it natively supports. If a tool does not edit the geometry, it has the ability to preserve the alternate representations during model editing (because the mapping of the model to the geometry is not stored in the geometry).

There are two general classes of geometric representation specification: those that explicitly specify surfaces and those that implicitly specify surfaces. For example, a level set is a field where a specific isosurface of the field specifies a geometric surface. A geometry described using constructive solid geometry of geometric primitives (e.g. spheres, cylinders) specifies directly which points are "inside" an object. Alternatively, explicit surface representations explicitly declare the set of points belonging to surfaces (e.g. polygonal tessellations).

### 3.2 Namespace URI and other declarations necessary for using this package

Every SBML Level 3 package is identified uniquely by an XML namespace URI. For an SBML document to be able to use a given Level 3 package, it must declare the use of that package by referencing its URI. The following is the namespace URI for this version of the Spatial package for SBML Level 3 Version 1 Core:

“`http://www.sbml.org/sbml/level3/version1/spatial/version1`”

In addition, SBML documents using a given package must indicate whether the package can be used to change the mathematical interpretation of a model. This is done using the attribute `required` on the `<sbml>` element in the SBML document. For the Spatial package, the value of this attribute must be “`true`”, because the use of the Spatial package can change the mathematical meaning of a model.

The following fragment illustrates the beginning of a typical SBML model using SBML Level 3 Version 1 Core and this version of the Spatial package:

```
<?xml version="1.0" encoding="UTF-8"?>
<sbml xmlns="http://www.sbml.org/sbml/level3/version1/core" level="3" version="1"
      xmlns:spatial="http://www.sbml.org/sbml/level3/version1/spatial/version1"
      spatial:required="true">
```

### 3.3 Primitive data types

The Spatial package uses a number of the primitive data types described in Section 3.1 of the SBML Level 3 Version 1 Core specification, and adds several additional primitive types described below.

#### 3.3.1 *Type* `SpId`

The type `SpId` is derived from `SId` (SBML Level 3 Version 1 Core specification Section 3.1.7) and has identical syntax. The `SpId` type is used as the data type for the identifiers of various objects in the Spatial Processes package. The purpose of having a separate type for such identifiers is to enable the space of possible spatial identifier values to be separated from the space of all other identifier values in SBML. The equality of `SpId` values is determined by an exact character sequence match; i.e., comparisons of these identifiers must be performed in a case-sensitive manner. All spatial objects with `id` attributes must have unique values among the other spatial objects in the same **Model**, but need not be unique among elements in other namespaces, such as the `SId` core namespace.

#### 3.3.2 *Type* `SpIdRef`

Type `SpIdRef` is used for all attributes that refer to identifiers of type `SpId`. It is derived from `SpId`, but with the restriction that the value of an attribute having type `SpIdRef` must match the value of a `SpId` attribute in the relevant model; in other words, the value of the attribute must be an existing spatial identifier in the referenced model. As with `SpId`, the equality of `SpIdRef` values is determined by exact character sequence match; i.e., comparisons of these identifiers must be performed in a case-sensitive manner.

#### 3.3.3 *Type* `BoundaryConditionKind`

The `BoundaryConditionKind` primitive data type is used in the definition of the **BoundaryCondition** class. It is derived from type `string` and its values are restricted to being one of the following possibilities: "`Neumann`" or "`Dirichlet`". Attributes of type `BoundaryConditionKind` cannot take on any other values. The meaning of these values is discussed in the context of the **BoundaryCondition** class's definition in Section 3.12 on page 16.

#### 3.3.4 *Type* `CoordinateKind`

The `CoordinateKind` primitive data type is used in the definition of the **CoordinateComponent** class. It is derived from type `string` and its values are restricted to being one of the following possibilities: "`cartesianX`", "`cartesianY`", and "`cartesianZ`". Attributes of type `CoordinateKind` cannot take on any other values. The meaning of these values is discussed in the context of the **CoordinateComponent** class's definition in Section 3.15 on page 18.

#### 3.3.5 *Type* `DataKind`

The `DataKind` primitive data type is used in the definition of the **SampledField** and **ParametricGeometry** classes. It is derived from type `string` and its values are suggested to be limited to the following three options: "`uint`", "`int`", or "`double`". For backwards compatibility, and for space reasons, the values "`float`", "`uint8`", "`uint16`", and "`uint32`" are also allowed. Attributes of type `DataKind` cannot take on any other values. The meaning of these values is discussed in the context of the classes' definitions in Section 3.16 on page 20 and Section 3.45 on page 40.

#### 3.3.6 *Type* DiffusionKind

The `DiffusionKind` primitive data type is used in the definition of the **DiffusionCoefficient** class. It is derived from type `string` and its values are restricted to being one of the following possibilities: "`isotropic`", "`anisotropic`", and "`tensor`". Attributes of type `DiffusionKind` cannot take on any other values. The meaning of these values is discussed in the context of the **DiffusionCoefficient** class's definition in Section 3.10 on page 15.

#### 3.3.7 *Type* CompressionKind

The `CompressionKind` primitive data type is used in the definition of the **SampledField** and **ParametricObject** classes. It is derived from type `string` and its values are restricted to being one of the following possibilities: "`uncompressed`", and "`deflated`". Attributes of type `CompressionKind` cannot take on any other values. The meaning of these values is discussed in the context of the classes' definitions in Section 3.46 on page 40, Section 3.47 on page 42, and Section 3.16 on page 20.

#### 3.3.8 *Type* FunctionKind

The `FunctionKind` primitive data type is used in the definition of the **AnalyticVolume** class. It is derived from type `string` and its values are restricted to being the single possibility "`layered`". Attributes of type `FunctionKind` cannot take on any other values. The meaning of these values is discussed in the context of the **AnalyticVolume** class's definition in Section 3.26 on page 29.

#### 3.3.9 *Type* GeometryKind

The `GeometryKind` primitive data type is used in the definition of the **Geometry** class. It is derived from type `string` and its values are restricted to being the single possibility "`cartesian`". Other `GeometryKind` types may be used in future versions of the specification. Attributes of type `GeometryKind` cannot take on any other values. The meaning of these values is discussed in the context of the **Geometry** class's definition in Section 3.17 on page 22.

#### 3.3.10 *Type* InterpolationKind

The `InterpolationKind` primitive data type is used in the definition of the **SampledField** class. It is derived from type `string` and its values are restricted to being one of the following possibilities: "`nearestNeighbor`" and "`linear`". Attributes of type `InterpolationKind` cannot take on any other values. The meaning of these values is discussed in the context of the **SampledField** class's definition in Section 3.16 on page 20.

#### 3.3.11 *Type* PolygonKind

The `PolygonKind` primitive data type is used in the definition of the **ParametricObject** class. It is derived from type `string` and its values are restricted to be the single value "`triangle`". Other `PolygonKind` types are held in reserve for future versions of this specification, and may include "`quadrilateral`". Attributes of type `PolygonKind` cannot take on any other values. The meaning of these values is discussed in the context of the **ParametricObject** class's definition in Section 3.47 on page 42.

#### 3.3.12 *Type* PrimitiveKind

The `PrimitiveKind` primitive data type is used in the definition of the **CSGPrimitive** class. It is derived from type `string` and its values are restricted to being one of the following possibilities: "`sphere`", "`cube`", "`cylinder`", and "`cone`", for three-dimensional **CSGObject** elements, and "`circle`", and "`square`", for two-dimensional **CSGObject** elements. Attributes of type `PrimitiveKind` cannot take on any other values. The meaning of these values is discussed in the context of the **CSGPrimitive** class's definition in Section 3.16 on page 20.

#### *3.3.13 Type* `SetOperation`

The `SetOperation` primitive data type is used in the definition of the **CSGSetOperator** class. It is derived from type `string` and its values are restricted to being one of the following possibilities: "`union`", "`intersection`", and "`difference`". Attributes of type `SetOperation` cannot take on any other values. The meaning of these values is discussed in the context of the **CSGSetOperator** class's definition in Section 3.36 on page 35.

#### *3.3.14 Type* `doubleArray`

The `doubleArray` primitive data type is a space-delimited list of `double` values in a single string.

#### *3.3.15 Type* `arrayData`

The `arrayData` primitive data type is used in the definition of the **SampledField** and **ParametricObject** classes. It consists of a possibly-compressed whitespace-and-semicolon-delimited list of numerical values in a single string. The meaning and possible content of these values is discussed in the context of the classes' definitions in Section 3.16 on page 20 and Section 3.45 on page 40.

### 3.4 The extended Model object

The **Model** object is extended in the spatial package to contain a new **Geometry** child, as seen in Figure 1. The **Geometry** element is contained in the Model element in the 'spatial' namespace. In order to specify a spatial geometry, some of the existing SBML elements need to be extended (**Compartment**, **Species**, **Parameter**, and **Reaction**). These extensions to the SBML elements are discussed in the sections that follow.

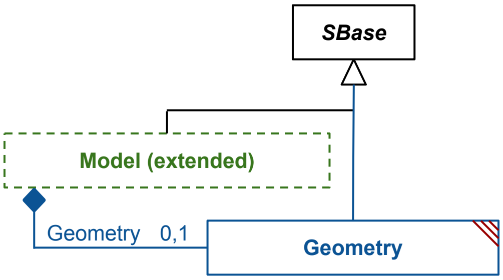

***Figure 1:*** *The definition of the extended* ***Model*** *object from the Spatial package. The* ***Geometry*** *object and its children are defined in their own sections.*

### 3.5 The extended Compartment object

The **Compartment** in the SBML core is extended while defining a spatial model. An SBML model with spatial geometry defines domain types (classes of domains that are anatomically and physiologically similar). These domain types need to be mapped to a compartment in the SBML model. **Compartments** are extended to define **CompartmentMappings** that map compartments to **DomainTypes** such that each corresponding **DomainType** is assigned the same biological and mathematical function. Within SBML L3 Core, the compartment Sid refers to the size of that compartment and is specified by the size attribute or may be set by a rule. For spatial models, the compartment size is calculated as the product of the unit size specified in the compartment mapping and the size of the current domain. The definition for the extension of the Compartment element is shown in Figure 2 on the following page.

The **Compartment** element has an optional **CompartmentMapping** child which indicates the **DomainType** to which the **Compartment** is mapped. If there is no **CompartmentMapping** for a **Compartment** in a spatial model, then that **Compartment** is excluded from the spatial version of the model. In the same way, if a **DomainType** is not mapped to

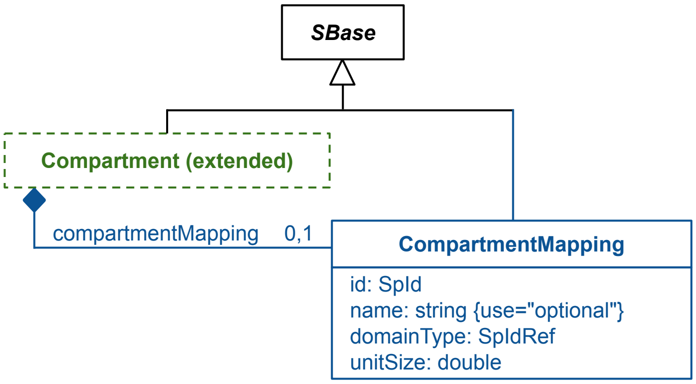

***Figure 2:** The definition of the extension to the **Compartment** element, and the definition of the **CompartmentMapping** class. The SBML core attributes of **Compartment** are not displayed.*

one or more **Compartments**, then the corresponding **Domains** in the geometry have no assigned function.

### 3.6 The CompartmentMapping class

Each **Compartment** in a model that defines a spatial geometry may contain an optional **CompartmentMapping**. A **CompartmentMapping** is defined as part of the model rather than part of the geometry so that the geometry is modular and may be readily shared between models and reused. A **CompartmentMapping** maps a **Compartment** defined in the model to a **DomainType** defined in the geometry such that each corresponding **DomainType** is assigned the same biological and mathematical function described by the set of **Compartments** that are mapped to that **DomainType**.

This mapping need not be one-to-one. In fact, it is common to map er-lumen, er-membrane, and cytosol to the same cell interior volume or 3D **DomainType**. The `unitSize` attribute specifies the relative quantity of each **Compartment** that is mapped to the **DomainType**.

#### *3.6.1 The `id` and `name` attributes*

The `id` attribute is a mandatory attribute of type `SpId` that is used to uniquely identify a **CompartmentMapping** in the model. All identifiers of type `SpId` must be unique within the **Model**. The mathematical value of a **CompartmentMapping** is its `unitSize` attribute, and can be bound to a **Parameter** by using a **SpatialSymbolReference** to the **CompartmentMapping** id. The optional `name` attribute is of type `string`, may be used to add a human-readable label to the object, and has no uniqueness requirements.

#### *3.6.2 The `domainType` attribute*

The mandatory `domainType` attribute is of type `SpIdRef` that indicates a **DomainType** defined in the **Geometry** element.

#### *3.6.3 The `unitSize` attribute*

The `unitSize` attribute is of type `double` and represents the relative size of the **Compartment** with respect to the size of the **Domains** to which they are mapped. Thus for any infinitesimal subset of the **Domain** with size S, there exists an amount of $\text{Compartment}_i$ of size $(S*\text{unitSize}_i)$ for i=1..N compartments mapped to that **DomainType**. For example, a 3D **Compartment** (and **DomainType**) which is mapped to a 3D **DomainType** has a `unitSize` which is a volume fraction of dimensionless unit. The total set of all such volume fractions mapped to a particular **DomainType** will typically sum to one.

If the `spatialDimensions` attribute of the parent **Compartment** is different than the `spatialDimensions` attribute of referenced **DomainType**, the `unitSize` attribute is a conversion factor between the two. The most common example of this would be a 2D **Compartment** being mapped to a 3D **DomainType**, such as an ER-membrane being mapped to a volumetric cell interior. In this case, the `unitSize` is a surface-to-volume ratio.

If connected to a **Parameter** via a **SpatialSymbolReference**, an **InitialAssignment** may override the value of the `unitSize` attribute. It is theoretically possible to have this value change in time through the use of a **Rule** or **Event**, but some (if not all) software tools may not support this setup. If the value is set to change, and the dimensionality of the parent **Compartment** and referenced **DomainType** is the same, the other **CompartmentMapping** elements for the same **DomainType** will typically change in concert, so that they continue to sum to one. Also note that **Species** amounts are preserved in SBML, so a changing `unitSize` may induce a corresponding change in any correlated **Species** concentration.

Any bound **Parameter**'s units should be equivalent to the units of the parent **Compartment** divided by the units of the referenced **DomainType**.

Here is an example of a **Compartment** element extended to have spatial information:

```
<compartment id="Extracellular" spatialDimensions="3" constant="true">
  <spatial:compartmentMapping spatial:id="spatialExtracellular"
      spatial:domainType="Extracellular" spatial:unitSize="1"/>
</compartment>
```

### 3.7 The extended Species object

The SBML core **Species** is extended when a spatial geometry is defined in the model with the addition of a single new required boolean "`isSpatial`" attribute. The extension to the **Species** element is shown in Figure 3.

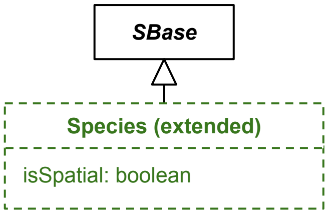

***Figure 3:*** *The extension to the **Species** element. The attributes of **Species** from SBML Level 3 Version 1 Core are not displayed.*

#### *3.7.1 The* `isSpatial` *attribute*

The `isSpatial` attribute is of data type boolean. If it is set to true, the **Species** is spatially distributed in a possibly nonhomogeneous manner within the **Domains** of the **DomainType** of the **Compartment** in which the **Species** resides.

For continuous deterministic models (described by partial differential equations), a spatial **Species** will result in a concentration field described by a partial differential equation which incorporates contributions from **Reactions**, diffusion (**DiffusionCoefficient**) and advection (**AdvectionCoefficient**) and are subject to boundary conditions (**BoundaryCondition**) and initial conditions (**InitialAssignment** and **Rule**). All of these quantities can be explicit functions of the spatial coordinates as well as spatial and nonspatial **Parameters** and **Species**.

For stochastic models, the **Species** is represented as a collection of particles that are distributed throughout the **Domains** and are subject to reactions, diffusion and advection. Simulation algorithms either track individual particles (e.g. Particle-based methods) or use spatial discretization to track a large number of well stirred pools (e.g. Next-Subvolume Method).

The `compartment` of any **Species** set `isSpatial` = "`true`" must have a child **CompartmentMapping**: if it did not, its compartment would not actually be a part of the spatial model.

### 3.8 The extended Parameter object

When an SBML model defines a spatial geometry, the SBML core **Parameter** is used to define the diffusion coefficient, transport velocity (advection) and boundary conditions for species and the coordinate components defined in the **Geometry**. One **Parameter** is created for each quantity, by adding a child **DiffusionCoefficient**, **AdvectionCoefficient**, or **BoundaryCondition**. Conversely, some elements defined in the spatial package may need to be referenced by mathematics in core constructs, or even have their value set by core constructs such as **InitialAssignment** or **Rule**. These spatial elements can be semantically linked to a **Parameter** by giving it a child **SpatialSymbolReference** pointing to that element.

A **Parameter** that has been extended for the Spatial package can have only one of the above listed objects. For example, if a **Parameter** is extended to represent the diffusion coefficient of a species, the existing attributes of the **Parameter** (id, name, value, units, constant) are defined according to SBML core specifications, along with a **DiffusionCoefficient** child that contains the information about the species it represents. Figure 4 represents the extension to the **Parameter** element.

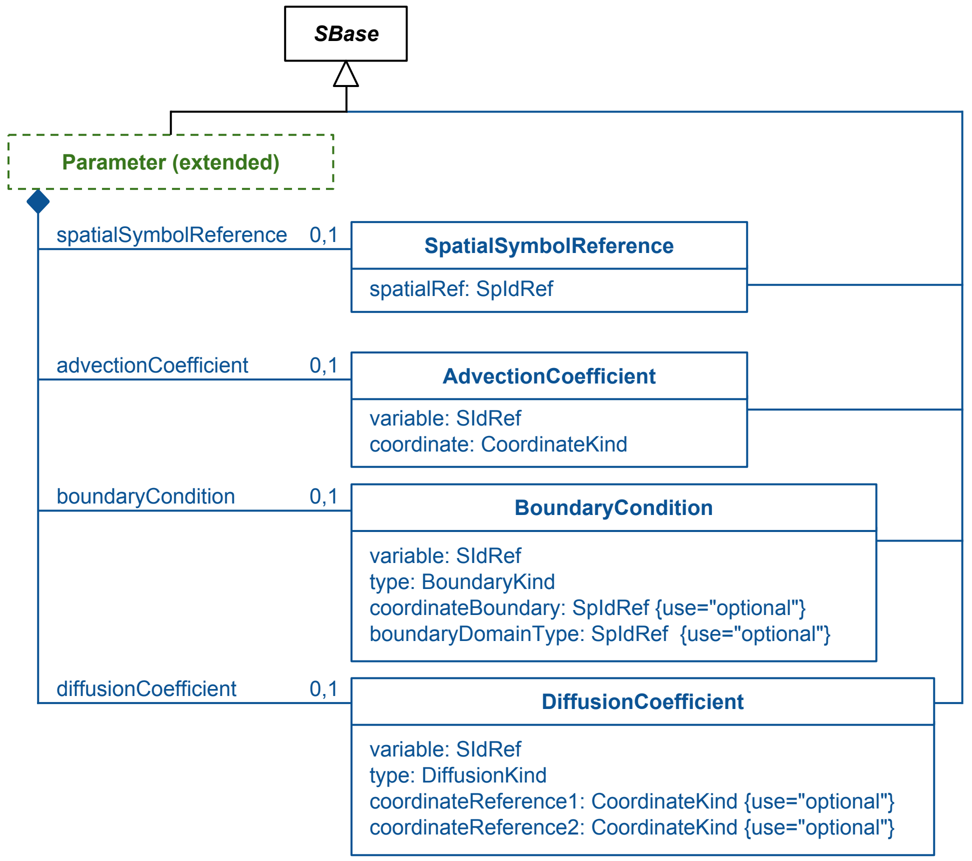

***Figure 4:*** *The **Parameter** element extension for spatial package, and the definitions of the **SpatialSymbolReference**, **AdvectionCoefficient**, **BoundaryCondition**, and **DiffusionCoefficient** classes. The SBML Level 3 Version 1 Core attributes for **Parameter** are not displayed in this figure.*

### 3.9 The SpatialSymbolReference class

A **Parameter** is extended with a **SpatialSymbolReference** element, when a symbol from the defined spatial geometry (`id` of any element contained in **Geometry**) is required to be used in the SBML core model. Typically, the **SpatialSymbolReference** is used to represent the coordinate components defined in the **Geometry**'s listOfCoordinateComponents. For example, if the **Geometry** is defined in a 2-dimensional Cartesian coordinate system with X and Y defined as coordinate components, two **Parameters** (one each for **CoordinateComponents** X and Y) are created in the model. The value of the parameter is not required to be set. For each of these parameters, a **SpatialSymbolReference** object is created.

#### *3.9.1 The* `spatialRef` *attribute*

The `spatialRef` attribute of **SpatialSymbolReference**, is of type `SpIdRef` and refers to the `SpId` of any element with mathematical meaning defined in the **Geometry** of the model.

### 3.10 The DiffusionCoefficient class

When a species in a spatial model has a diffusion rate constant, a **Parameter** for this diffusion constant is created in the SBML model with a **DiffusionCoefficient** child, which is used to identify the **Species** whose diffusion rate the **Parameter** represents. The diffusion coefficient can then be set like any other variable: its initial value can be set using the **Parameter**'s `value` attribute or through an **InitialAssignment**, and if the diffusion coefficient changes in time, this can be defined with a **Rule** or **Event**. If set, the units of this **Parameter** should be $length^2/time$. If left unset, the **DiffusionCoefficient** will inherit the model units of $length^2/time$ (typically $cm^2 s^{-1}$ or $um^2 s^{-1}$).

It is possible to define both diffusion and advection for the same **Species**.

#### *3.10.1 The* `variable` *attribute*

The required `variable` attribute of **DiffusionCoefficient** is of type `SIdRef` and is the `SId` of the **Species** or **Parameter** in the model whose diffusion coefficient is being set.

#### *3.10.2 The* `type` *and* `coordinateReference` *attributes*

The required `type` attribute of **DiffusionCoefficient** is of type `DiffusionKind` and indicates whether the diffusion coefficient is "`isotropic`" (i.e. applies equally in all dimensions/directions), "`anisotropic`" (i.e. applies only for a single coordinate), or "`tensor`" (i.e. applies only for a particular pair of coordinates). Coefficients of type "`isotropic`" may not have any `coordinateReference` attributes defined, since diffusion is defined for all axes. Coefficients of type "`anisotropic`" must define the `coordinateReference1` attribute and not the `coordinateReference2` attribute, and applies in the direction of that axis. Coefficients of type "`tensor`" must define both the attributes `coordinateReference1` and `coordinateReference2`, defining diffusion in relation to the direction due to a gradient in the diagonal term of the diffusion tensor for the two coordinates. In no case may `coordinateReference2` be defined but not `coordinateReference1`.

#### *3.10.3 DiffusionCoefficient uniqueness*

Only one **DiffusionCoefficient** may be defined per **Species** per axis or pair of valid axes in the **Compartment** in which it resides. Since isotropic diffusion is defined for all axes at once, this means that if an isotropic **DiffusionCoefficient** is defined for a **Species**, it may have no other diffusion coefficients. Similarly, if a tensor **DiffusionCoefficient** is defined for a **Species** for a pair of axes, the model must not also define an anisotopic **DiffusionCoefficient** for either of those axes. This means that the following types of diffusion coefficients are allowed in a three-dimensional **Geometry**:

- A single isotropic diffusion coefficient.
- Up to three tensor diffusion coefficients, one per pair of axes.
- Up to three anisotropic diffusion coefficients, one per axis.
- One tensor diffusion coefficient for one pair of axes, and a second anisotropic diffusion coefficient, for the third axis.

For a two-dimensional **Geometry**, the following are allowed:

- A single isotropic diffusion coefficient.
- A single tensor diffusion coefficient, defined for the two axes (equivalent to a single isotropic diffusion coefficient).
- Up to two anisotropic diffusion coefficients, one per axis.

For a one-dimensional **Geometry**, the following are allowed:

- A single isotropic diffusion coefficient.
- A single anisotropic diffusion coefficient, defined for the single axis (equivalent to a single isotropic diffusion coefficient).

Tensor diffusion coefficients may not be defined for one-dimensional geometries, as they apply in two dimensions.

### 3.11 The AdvectionCoefficient class

The **AdvectionCoefficient** is the extension to **Parameter** in SBML core that is used to represent transport velocity of a species, if it exists. The transport velocity for the species is defined in a manner similar to the diffusion constant with a unit of length/time (regardless of the units of the corresponding **Species**' "`compartment`" attribute). A **Parameter** is created in SBML code for the velocity with an **AdvectionCoefficient** child to identify the **Species** whose velocity is represented by the **Parameter**; its initial value is set either through the `value` attribute or an **InitialAssignment**. If the advection coefficient changes in time or space, this can be modeled with a **Rule** or **Event**.

If defined, the units of the parent **Parameter** should be in length/time; if not defined, it inherits from the model-wide units of length divided by the model-wide units of time.

It is possible to define both diffusion and advection for the same **Species**.

#### 3.11.1 The `variable` attribute

The `variable` attribute of **AdvectionCoefficient** is of type `SIdRef` and is the SId of the **Species** or **Parameter** in the model whose advection coefficient (transport velocity) is being set.

#### 3.11.2 The `coordinate` attribute

The `coordinate` is of type `CoordinateKind` and represents the coordinate component of the velocity. For example, if the **Geometry** is defined in the Cartesian coordinate system and is 2-dimensional, the species can have velocity terms for both X and Y. If the **Parameter** represents the transport velocity of the species in the X-coordinate, the `coordinate` attribute will take a value of "`cartesianX`", and if it represents the velocity in the Y-coordinate, the attribute will take a value of "`cartesianY`". Only one **AdvectionCoefficient** may be defined per **Species** per valid `coordinate`.

### 3.12 The BoundaryCondition class

A **Species** in a spatial model that has a diffusion rate or an advection velocity needs to have specified boundary conditions. A boundary condition is either the concentration of the species or the flux density of the species at a boundary. The boundary refers to either an internal membrane boundary or a face of the box defined by the minimum and maximum coordinates of the geometry (the geometries bounding box).

When creating a spatial SBML model, species boundary conditions are created as parameters, one for each boundary condition, by adding a child **BoundaryCondition** that points to the corresponding **Species** and boundary, depending on the coordinate system.

For Cartesian Geometries, there are two boundaries for every axis being modeled. For example, in a 2D cartesian geometry for the external boundaries, there could be up to four parameters or parameter sets created for each spatial **Species** whose **Compartments** abut the minimum and maximums of the X and Y axes).

For internal boundaries, one may either define a **BoundaryCondition** for a **Species** at that boundary, or one may define one or more transport reactions that describe how the physical entities that **Species** represent are moved (or converted) from one side of the boundary to the other. One may not define both a **BoundaryCondition** and a **Reaction** that describes the same phenomenon, as this would result in the equivalent of an overdetermined system, not dissimilar from the reason that the change in a **Species** may not be defined by both a **Reaction** and a **RateRule**.

If neither a **BoundaryCondition** nor a **Reaction** is defined for a particular **Species**/boundary pair, the flux of that **Species** at that boundary is zero.

The `boundaryCondition` attribute on an SBML core **Species** defines whether it is ("`false`") or is not ("`true`") changed by any **Reaction** it may appear in as a product or reactant. In a spatial context, setting this attribute to "`true`" additionally means that is will not be changed by advection, diffusion, or (confusingly) any **BoundaryCondition**. This is because the 'boundary' of a **BoundaryCondition** refers to a physical boundary, while the 'boundary' of the `boundaryCondition` attribute refers to the conceptual boundary of the reaction network. As in SBML Core, a **Species** with a `boundaryCondition` of "`true`" may only be changed by a **Rule** (**AssignmentRule**, **RateRule**, or **AlgebraicRule**).

The **Parameter**'s value is set either through the `value` attribute or an **InitialAssignment**. If the boundary condition changes in time, it can be set with a **Rule** or **Event**. If set, the **Parameter** unit must be equal to the appropriate unit for its `type` (see below). Only one **BoundaryCondition** may be defined per **Species** per boundary (regardless of type).

#### *3.12.1 The `variable` attribute*

The `variable` attribute of **BoundaryCondition** is of type `SIdRef` and is the SId of the **Species** or **Parameter** in the model whose boundary condition is being set.

#### *3.12.2 The `type` attribute*

The `type` attribute is of type `BoundaryConditionKind` and indicates the type of boundary condition. The boundary condition types come in two groups: for Neumann boundaries, "`Neumann`" (the inward normal flux) is used. For Dirichlet boundaries, "`Dirichlet`" (the value) is used.

The unit of the boundary condition is determined by the type, and the unit for density and velocity. For "`Dirichlet`", the unit would be the unit of concentration. For "`Neumann`", the unit would be concentration*length/time.

#### *3.12.3 The `coordinateBoundary` attribute*

The `coordinateBoundary` attribute is of type `SpIdRef` and refers to the `SpId` of either the `boundaryMin` or the `boundaryMax` object of the **CoordinateComponent** defined in **Geometry**. This `SpId` indicates the boundary condition (minimum or maximum) in the **CoordinateComponent**. A **Parameter** that is extended with a **BoundaryCondition** object can only define the `coordinateBoundary` attribute or the `boundaryDomainType` attribute, but not both.

#### *3.12.4 The `boundaryDomainType` attribute*

The `boundaryDomainType` attribute is of type `SpIdRef` and refers to the `SpId` of the **DomainType** of the location of the species whose boundary condition is being defined. A **Parameter** that is extended with a **BoundaryCondition** object can only define the `coordinateBoundary` attribute or the `boundaryDomainType` attribute, but not both.

### 3.13 Extended Parameter examples

As an example, here are four examples of parameters that have been extended with spatial information. These four parameters are extended with the four different possible spatial extensions: the **SpatialSymbolReference** to allow the model to refer to the spatial id "x" as the SBML core id "x"; a **BoundaryCondition** to define the boundary condition for "s1_nuc" to be zero; a **DiffusionCoefficient** to define the diffusion coefficient for "s1_nuc" to be "0.1"; and an **AdvectionCoefficient** to define the advection coefficient for "s2_nuc" to be "0.13".

```
<listOfParameters>
  <parameter id="x" constant="false">
    <spatial:spatialSymbolReference spatial:spatialRef="x"/>
  </parameter>
  <parameter id="s1_nuc_BC_Xm" value="0" constant="true">
    <spatial:boundaryCondition spatial:variable="s1_nuc" spatial:type="Dirichlet"
                               spatial:coordinateBoundary="Xmin"/>
  </parameter>
  <parameter id="s1_nuc_diff" value="0.1" constant="true">
    <spatial:diffusionCoefficient spatial:variable="s1_nuc" spatial:type="isotropic"/>
  </parameter>
  <parameter id="s2_nuc_advec" value="0.13" constant="true">
    <spatial:advectionCoefficient spatial:variable="s2_nuc" spatial:coordinate="cartesianX"/>
  </parameter>
</listOfParameters>
```

### 3.14 The extended Reaction object

The SBML core **Reaction** is extended when a spatial geometry is defined in the model with the addition of a single new required boolean isLocal attribute. Figure 5 displays the definition of the extension of the **Reaction** element.

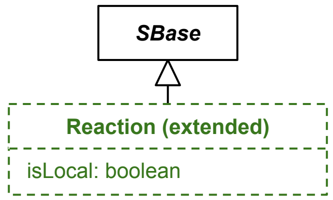

***Figure 5:*** *The extension to the* ***Reaction*** *element. The SBML Level 3 Version 1 Core atrributes and children for* ***Reaction*** *are not displayed in the figure.*

#### *3.14.1 The* isLocal *attribute*

The isLocal attribute for a **Reaction** is of type Boolean. The attribute is set to true if the reaction is to be considered a local description of the reaction in terms of concentration/time defined at each point in space rather than substance/time over an entire **Compartment** or "pool". Note that this means that the units of the **KineticLaw** are different depending on whether the **Reaction** is local or not.

If a **Reaction** is defined to be a local (having an isLocal value of "true"), the value of the compartment attribute of the **Reaction** must be defined. This is because the interpretation of the **Reaction** is very different if the reaction takes place at the boundary of the **Compartment** of the **Species** (where the reaction rate units are flux densities) than if it takes place within the interior of that **Compartment** (where the reaction rate units are concentration/time define throughout the volume). The first will give you gradients in the solution, while the other will not.

If the referenced **Species** come from multiple compartments, the compartment of the **Reaction** must be a **Compartment** that makes physical sense for the individual **Species** to meet.

### 3.15 The CoordinateComponent class

A **CoordinateComponent** object explicitly defines a coordinate component of the coordinate axes and gives them names, units, and formally associates them with a coordinate system. The **CoordinateComponent** also defines the minimum and maximum values of the coordinate axis it represents. The definition of **CoordinateComponent** is shown in Figure 6.

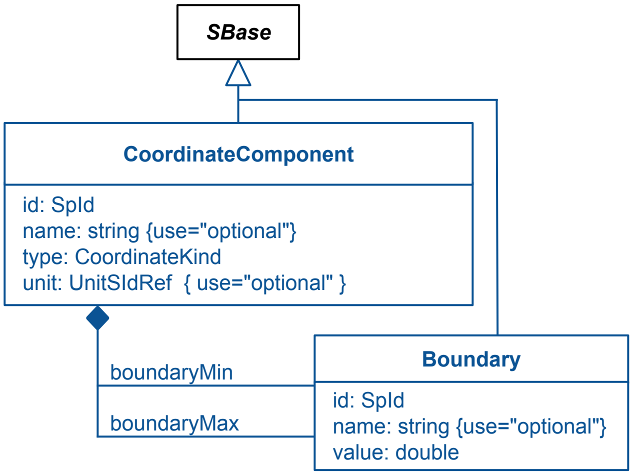

***Figure 6:*** *The **CoordinateComponent** and **Boundary** object definitions. One or more instances of **CoordinateComponent** objects in a **ListOfCoordinateComponents** can be present in **Geometry**.*

#### *3.15.1 The `id` and `name` attributes*

A **CoordinateComponent** is identified with the `id` attribute which is of type `SpId`. The mathematical value of a **CoordinateComponent** is the local value of the coordinate, and can be bound to a **Parameter** by using a **SpatialSymbolReference** to the **CoordinateComponent** id. The optional `name` attribute is of type `string`, may be used to add a human-readable label to the object, and has no uniqueness requirements.

Because a **CoordinateComponent** represents an entire axis, it is not appropriate (should it be connected to a **Parameter** via a **SpatialSymbolReference**) for it to be set via an **InitialAssignment** or **Rule**. Rather, it is treated like the SBML core `csymbol` "`time`", and can be used as an independent variable in other calculations.

#### *3.15.2 The `type` attribute*

The `type` attibute of type `CoordinateKind` represents the type of the coordinate component, and may take one of a subset of all possible `CoordinateKind` values depending on its parent **Geometry**, as defined in Table 1 on the next page. For Cartesian geometries, one-dimensional geometries must be defined by having a single "`cartesianX`" **CoordinateComponent**; two-dimensional geometries must be defined by having exactly two **CoordinateComponent** children with `type` values of "`cartesianX`" and "`cartesianY`", and three-dimensional geometries must be defined by having exactly three **CoordinateComponent** children with `type` values of "`cartesianX`", "`cartesianY`", and "`cartesianZ`". No **Geometry** may have two **CoordinateComponent** children with the same `type`.

#### *3.15.3 The `unit` attribute*

The unit of a **CoordinateComponent** is represented by the `unit` attribute, of type `UnitSIdRef`. If not specified, the unit of a **CoordinateComponent** inherits from the `lengthUnits` attribute of the **Model** object, and if that in turn is not specified, the **CoordinateComponent** units cannot be determined.

| GeometryKind | Dimensions | CoordinateKinds |
|---|---|---|
| `cartesian` | 1 | "`cartesianX`" (`coord1`) |
| `cartesian` | 2 | "`cartesianX`" (`coord1`) and "`cartesianY`" (`coord2`) |
| `cartesian` | 3 | "`cartesianX`" (`coord1`), "`cartesianY`" (`coord2`), and "`cartesianZ`" (`coord3`) |

***Table 1:*** *Correspondance between the `type` of a **Geometry** and the possible `type`s of its child **CoordinateComponent** elements. Also noted is the corresponding attribute (`coord1`, `coord2`, or `coord3`) corresponding to each axis when defining **InteriorPoint** elements (see Section 3.21 on page 27).*

### 3.16 The SampledField class

A **SampledField** is a sampled scalar field such as an image or samples from a level set. The attributes of **SampledField** represent the specification of a sample dataset (the number of samples in x, y, z coordinates, data type of the sample representation, etc.) and the text child of the **SampledField** is the actual sampled data, defined in Figure 7. The purpose of a **SampledField** is to fill the **Geometry** with values that can be used in math and/or in **SampledFieldGeometry** elements. Values are defined at lattice points within the **Geometry**, and are interpolated to fill the remainder of all off-lattice locations.

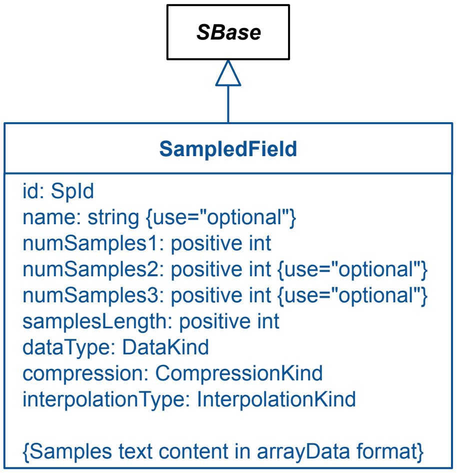

***Figure 7:*** *The definition of the **SampledField** class from the Spatial package.*

#### *3.16.1 The `id` and `name` attributes*

The `id` attribute identifies a **SampledField**. It is of type `SpId` and is a required attribute. The mathematical value of a **SampledField** is the value and dimensionality of the field itself, and can be bound to a **Parameter** by using a **SpatialSymbolReference** to the **SampledField** id. If used in conjunction with the SBML Level 3 "`arrays`" package, it can be used and manipulated as if it was an array of the appropriate dimensions, even though its *meaning* is the value of the field at all points within its borders, not just those at the lattice points. However, even without the use of the "`arrays`" package, it can be used in SBML Level 3 Version 1 Core MathML to set the value of a spatially-distributed SBML symbol such as a **Species** or **Parameter**, such as through an **InitialAssignment**, **Rule**, or **EventAssignment**. The optional `name` attribute is of type `string`, may be used to add a human-readable label to the object, and has no uniqueness requirements.

The size of the field is assumed to match the axes (the **CoordinateComponent** children) of the parent **Geometry**, and is assumed to be regularly spaced in each dimension, but is not required to be spaced the same way in all dimensions. In other words, if the Geometry defines a 10 cm by 10 cm square, and a SampledField is a 10x5 array, the "`[0,0]`" entry in the array will correspond to the point "`0 cm, 0 cm`" in the Geometry, and the "`[9,4]`" entry in the array will correspond to the point "10 cm, 10 cm" in the Geometry. Off-latice points (such as the value at "9 cm, 9 cm" in this example) have no direct corresponding value in the **SampledField**, and are determined according to the interpolationType attribute, defined below.

When tied to a **SpatialSymbolReference**, regardless of its useage, each **SampledField** still must represent values across the entire **Geometry**. If used in an **InitialAssignment** to assign values to a **Species** that only exists in a particular **DomainType** within the **Geometry**, entries in the **SampledField** that correspond to areas of space not covered by that **DomainType** will simply be ignored. Those values may be set to zero, or could be used in other contexts. For example, a **SampledField** could represent 'the concentration of ATP in the Geometry', and one **InitialAssignment** could be used to apply the field to the species 'ATP in the cytosol' and a second **InitialAssignment** could be used to apply the same field to the species 'ATP in the nucleus', with different values being examined and used in each case.

When used in a **SampledFieldGeometry**, again, the values of the field are interpolated to all off-lattice points to determine whether or not any arbitrary location within the **Geometry** is part of a given **SampledVolume** or not.

#### 3.16.2 The interpolationType attribute

The required interpolationType attribute is type InterpolationKind. It is used to specify how values at off-lattice locations are to be calculated. A value of "nearestNeighbor" means that the nearest lattice point value is to be returned, calculated using the L2 norm (or Euclidean distance). A value of "linear" means that the value to be returned is the linear interpolation from nearby lattice points, either simple linear in the case of one-dimensional interpolation, bilinear in the case of two-dimensional interpolation, or trilinear in the case of three-dimensional interpolation.

#### 3.16.3 The numSamples1, numSamples2, numSamples3 attributes

The numSamples1, numSamples2, and numSamples3 attributes represent the number of samples in each of the coordinate components. (e.g. numX, numY, numZ) in an image dataset. These attributes are of type positive int and are required to specify the **SampledField**. The samples are assumed to be uniformly sampled. It is required to have as many numSamples attributes as there are **CoordinateComponent** elements in the **Geometry**, with numSamples1 defined if there is a cartesianX element; numSamples2 defined if there is a cartesianY element, and numSamples3 defined if there is a cartesianZ element, each attribute corresponding to the **CoordinateComponent** with the respective type.

#### 3.16.4 The compression attribute

The required compression attribute is of type CompressionKind. It is used to specify the compression used when encoding the data, and can have the value "uncompressed" if no compression was used, or "deflated" if the deflation algorithm was used to compress the data. The deflation compression algorithm to be used is gzip, which adds a header to the deflated data. This algorithm is freely available. The version of the data to be compressed is the string version of the values in the array, which may consist of numbers, whitespace, commas, and semicolons.

#### 3.16.5 The samplesLength attribute

The samplesLength attribute is of type positive int and is required. It represents the array length of the arrayData text child of this node. If uncompressed, this will equal the product of the numSamples* attributes, but if compressed, this will equal the new compressed length of the array, not including any added whitespace. It is included for convenience and validation purposes.

#### 3.16.6 The dataType attribute

The dataType attribute is of type DataKind and is optional. It is used to specify the type of the data being stored, so that the uncompressed data can be stored in an appropriate storage type. The three main value types are "uint" for unsigned integers, "int" for signed integers, and "double" for double-precision floating point values. For backwards compatibility, and for cases where storage space might be an issue, other values may also be used:

"float" to indicate single-precision (32-bit) floating point values, and "uint8", "uint16", and "uint32" to indicate 8-bit, 16-bit, and 32-bit unsigned integer values, respectively.

#### *3.16.7 The* Samples *text child*

The Samples text child of the **SampledField** is where the data for the **SampledField** resides. It is of type arrayData, which is defined as whitespace-delimited, possibly-compressed numerical values. Whether or not the data is compressed (and how, if so) is stated with the compression attribute, and the type of numerical values included is stated in the dataType attribute. The total number of entries in the array can be derived from the numSamples attributes, by multiplying them together (if present). A semicolon may be used in uncompressed data to visually distinguish grouped values.

The order of data points in the Samples should be the same as the dimensionality of the object, that is, first by the first (x) dimension, then by the second (y) dimension (if present), and then by the third (z) dimension (if present). Thus, the array is indexed such that to access data point (x, y, z), one would look in entry:

$$samples[x + numSamples1 * y + numsamples1 * numSamples2 * z]$$

#### *3.16.8 A **SampledField** example*

Here are snippets of an example in which a **SampledField** element is used in an initial assignment, where the field's value inside the nucleus ("img") is used in a formula to compute the ATP level:

```
[...]
  <species id="ATP_nuc" compartment="Nucleus" hasOnlySubstanceUnits="false"
           boundaryCondition="false" constant="false" spatial:isSpatial="true"/>
[...]
  <parameter id="img" constant="false">
    <spatial:spatialSymbolReference spatial:spatialRef="imgvals"/>
  </parameter>
[...]
  <initialAssignment symbol="ATP_nuc">
    <math xmlns="http://www.w3.org/1998/Math/MathML">
      <apply>
        <plus/>
        <apply>
          <divide/>
          <ci> img </ci>
          <cn type="integer"> 2 </cn>
        </apply>
        <cn type="integer"> 3 </cn>
      </apply>
    </math>
  </initialAssignment>
[...]
  <spatial:sampledField spatial:id="imgvals" spatial:dataType="uint8"
           spatial:numSamples1="51" spatial:numSamples2="59"
           spatial:numSamples3="23" spatial:interpolationType="linear"
           spatial:compression="uncompressed" spatial:samplesLength="69207">
    120 218 237 221 235 114 219 48 [...]
  </spatial:sampledField>
[...]
```

### 3.17 The Geometry class

A single geometry must be defined within the model if the spatial extension is to be used. Figure 8 on the next page shows the definition of the **Geometry** element.

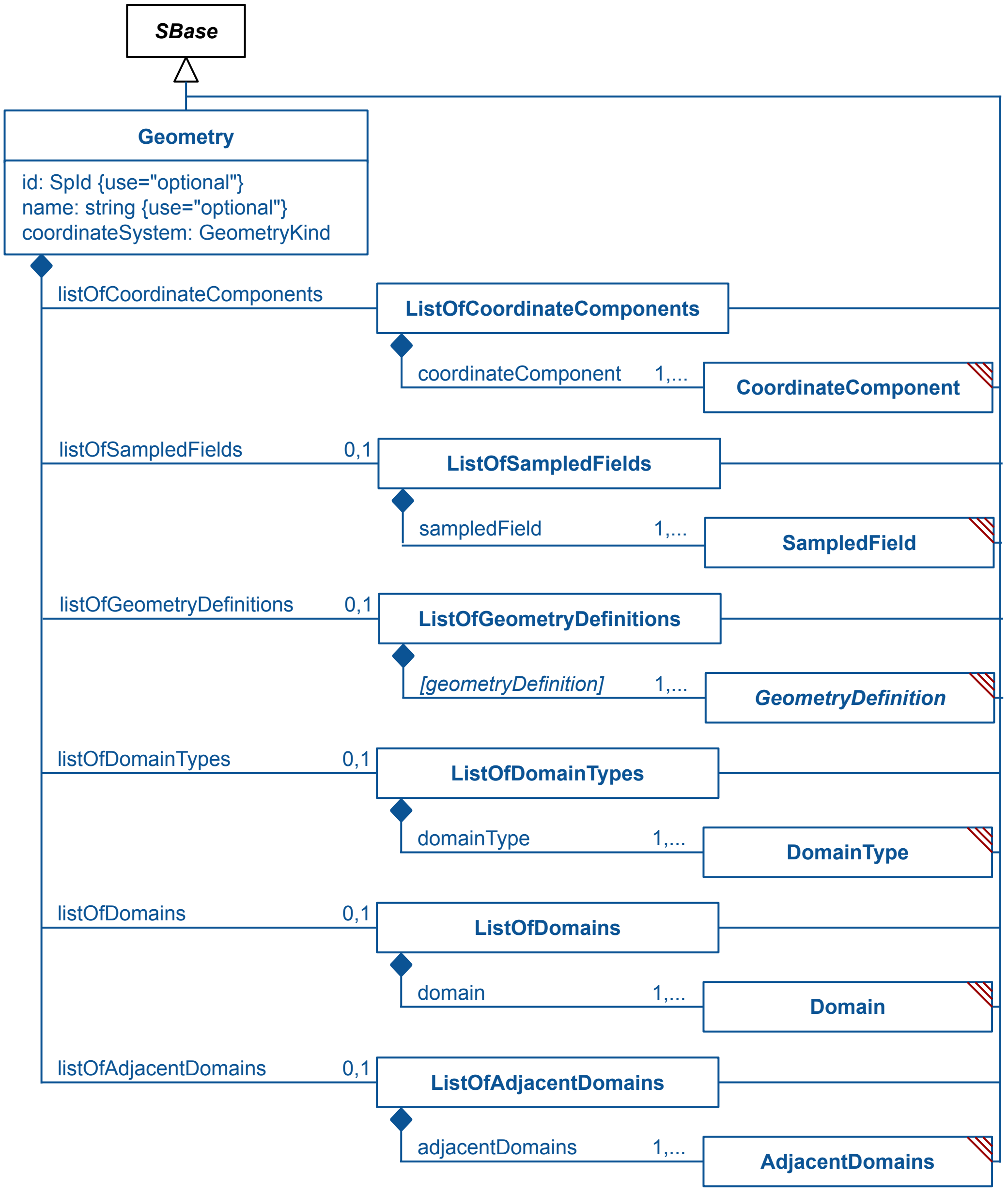

***Figure 8:*** *The definition of the* ***Geometry****,* ***ListOfCoordinateComponents****,* ***ListOfDomainTypes****,* ***ListOfDomains****,* ***ListOfAdjacentDomains****,* ***ListOfGeometryDefinitions****, and* ***ListOfSampledFields*** *classes from the Spatial package. The various children of the ListOf- classes are defined in their own sections.*

#### *3.17.1 The `id` and `name` attributes*

The `id` attribute is of type `SpId`, uniquely identifies the **Geometry** element, and is optional. It has no mathematical meaning, and cannot be connected to a **Parameter** via a **SpatialSymbolReference** element. The optional `name` attribute is of type `string`, may be used to add a human-readable label to the object, and has no uniqueness requirements.

#### 3.17.2 The coordinateSystem attribute

The coordinateSystem attribute is a required attribute and is of type GeometryKind. It represents the coordinate system used by the **Geometry**. A value of "cartesian" indicates that the geometry is a cartesian coordinate system, with the coordinate components corresponding to the x, y, and z components of that system (which could be 1-, 2-, or 3-dimensional). This is the only coordinate system defined in this version of the specification–in the future, if necessary, "cylendrical", "spherical", and "polar" may be added as possibilities, along with n-dimensional cartesian modeling, should there be interest in the modeling community to exchange these types of models.

#### 3.17.3 The listOf container classes

The **Geometry** has listOfCoordinateComponents, listOfDomainTypes, listOfDomains, listOfAdjacentDomains, listOfGeometryDefinitions, and listOfSampledFields that help define the geometry. The **ListOfCoordinateComponents** is a list of **CoordinateComponent** objects, the **ListOfDomainTypes** is a list of **DomainType** objects, the **ListOfDomains** is a list of **Domain** objects, **ListOfAdjacentDomains** is a list of **AdjacentDomains** objects, the **ListOfGeometryDefinitions** is a list of alternative ***GeometryDefinitions*** (**ParametricGeometry**, **CSGeometry**, **SampledFieldGeometry**, **AnalyticGeometry**, and **MixedGeometry**), and the **ListOfSampledFields** is a list of **SampledField** objects. Of these, only the **ListOfCoordinateComponents** is required, but, if present, none of them may be empty.

Note that the children of the **ListOfGeometryDefinitions** object are not called geometryDefinition but rather take the name of the derived class, decapitalized. Thus, they may be called analyticGeometry, csGeometry, parametricGeometry, sampledFieldGeometry, or mixedGeometry.

The **ListOfCoordinateComponents** is unique in that it defines the dimensions of the **Geometry**. Thus, every **Geometry** must have a child **ListOfCoordinateComponents** with exactly one, two, or three children. When there is one child, it must be of type cartesianX; when there are two, they must be of type cartesianX and cartesianY, and when there are three, they must be of types cartesianX, cartesianY, and cartesianZ.

### 3.18 The Boundary class

The minimum and the maximum for a **CoordinateComponent** represent the bounds in each coordinate. For example, for three dimensional Cartesian coordinate system with x, y, and z coordinates, the minimum and maximum limits for each coordinates define planes orthogonal to each coordinate axis and passing through the minimum or maximum. If max-min is the same for each x,y,z then the bounds on the geometry is a cube. The **Boundary** class interacts with the **BoundaryCondition** class, allowing modelers to define how model elements behave at the boundary of the model. For species defined within volumes adjacent to these surfaces, **BoundaryCondition** elements must be introduced.

The minimum limit of a **CoordinateComponent** is represented by the boundaryMin object and the maximum limit is represented by the boundaryMax object, and apply to **CoordinateComponent** elements. Both are **Boundary** objects, and have the following attributes:

#### 3.18.1 The id and name attributes

The id attribute of the **Boundary** object identifies the object. The attribute is required and is of type SpId. This attribute is used when specifying the **BoundaryCondition** for a species as an extension of an SBML core **Parameter**. The mathematical value of a **Boundary** is its value attribute, and can be bound to a **Parameter** by using a **SpatialSymbolReference** to the **Boundary** id. The units are the same as its parent **CoordinateComponent**, and are not set separately. The optional name attribute is of type string, may be used to add a human-readable label to the object, and has no uniqueness requirements.

#### 3.18.2 The value attribute

The value attribute is of type double. In a boundaryMin object, it represents the minimum limit of the **CoordinateComponent**. In a boundaryMax object, it represents the maximum limit of the **CoordinateComponent**.

If connected to a **Parameter** via a **SpatialSymbolReference**, this `value` may be overridden by an **InitialAssignment**. However, the **Parameter** must be set `constant`=“`true`”, because it may not change over the course of the simulation.

#### *3.18.3 A CoordinateComponent example*

In this example, a **CoordinateComponent** is used to define an X axis that goes from 0 to 10 um.

```
<spatial:coordinateComponent spatial:id="x" spatial:type="cartesianX" spatial:unit="um">
  <spatial:boundaryMin spatial:id="Xmin" spatial:value="0"/>
  <spatial:boundaryMax spatial:id="Xmax" spatial:value="10"/>
</spatial:coordinateComponent>
```

### 3.19 The DomainType class

A **DomainType** is a class of domains that are identified as being anatomically and physiologically similar. For example, a **DomainType** "cytosol" may be defined in a **Geometry** as identifying the structure and function of the cell interior. If there is one cell, then there is one domain, if there are multiple cells, then there are multiple disjoint domains ("cytosol1","cytosol2", etc.) identified with the **DomainType** "cytosol". **CompartmentMappings**, defined as an extension to an SBML core **Compartment**, map compartments to domain types such that each corresponding domain is assigned the same biological and mathematical function. Figure 9 shows the **DomainType** object.

Each SBML **Compartment** maps to a single **DomainType**, meaning that the initial condition of each **Species** in each **Compartment** will be defined consistently across **Domains** that map to a given **DomainType**. If those **Species** are spatially distributed, they will subsequently evolve independently from each other. However, if modeling two **Domains** that are similar but whose **Species** have initial conditions that must be defined in different ways, those **Domains** should be modeled as separate **DomainTypes**.

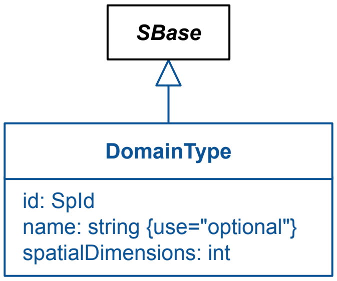

***Figure 9:*** *The definition of the **DomainType** class. One or more instances of **DomainType** in a **ListOfDomainTypes** instance can be present in a **Geometry** object.*

#### *3.19.1 The `id` and `name` attributes*

Each **DomainType** is identified with a `id` of type `SpId`. The mathematical value of a **CompartmentMapping** is the sum of the sizes of all domains associated with this **DomainType**, and can be bound to a **Parameter** by using a **SpatialSymbolReference** to the **CompartmentMapping** id. The optional `name` attribute is of type `string`, may be used to add a human-readable label to the object, and has no uniqueness requirements.

As a derived quantity, if connected to a **Parameter** via a **SpatialSymbolReference**, this value may *not* be overridden by an **InitialAssignment**, nor by the use of a **Rule** or **Event**. Its value is always connected to the size of its component **Domains** instead. The units of a **DomainType** are the units of the corresponding base units of the SBML **Model** for length (for one-dimensional domains), area (for two-dimensional domains), or volume (for three-dimensional domains). It is required to define the corresponding base units for every **DomainType** in the **Model**.

#### *3.19.2 The* `spatialDimensions` *attribute*

The `spatialDimensions` attribute of the **DomainType** is of type `int` and can take on a value of 0, 1, 2, or 3. The spatial dimension is specified for a **DomainType**, rather than being repeated for each **Domain** that is represented by the **DomainType**.

The `spatialDimensions` attribute of a **DomainType** may be the same dimensionality as the **Geometry** to which it refers (via the **Domain**), or may be one less. In the latter case, it is considered to describe the surface of that **Geometry** in the 3D case, the perimeter of the **Geometry** in the 2D case, or the end point of the **Geometry** in the 1D case. Since there is no defined perimeter of a 3D object, it is illegal to have a **DomainType** with a `spatialDimensions` of "`1`" where the corresponding **Geometry** is three-dimensional. Similarly, there is no end point of a 3D or 2D object, making it illegal to have a **DomainType** with a `spatialDimensions` of "`0`" where the corresponding **Geometry** is two- or three-dimensional. A **DomainType** may also not have a higher dimensionality than the **Geometry**.

### 3.20 The Domain class

**Domains** represent contiguous regions identified by the same **DomainType**. One, two, and three dimensional domains are contiguous linear regions, surface regions, and volume regions (respectively), bounded by the limits of the coordinate system (e.g. min/max of x,y,z) and adjacent domains corresponding to different domain types. Zero-dimensional domains are also allowed as boundaries of one-dimensional geometry domains. **Domain** is shown in Figure 10.

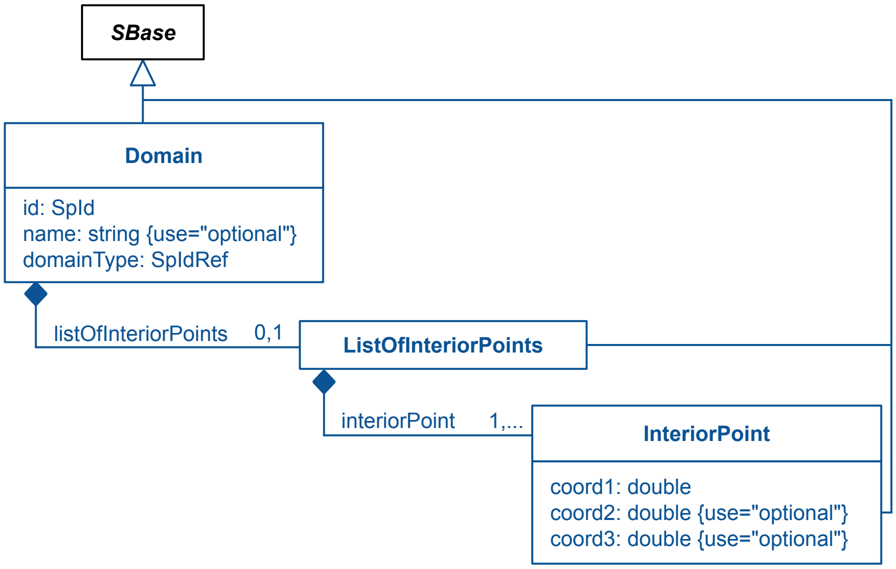

***Figure 10:*** *The definition of the **Domain**, **ListOfInteriorPoints**, and **InteriorPoint** classes. A **ListOfDomains** instance in **Geometry** can contain one or more **Domain** object instances.*

#### *3.20.1 The* `id` *and* `name` *attributes*

A **Domain** is identified with an `id` attribute of type `SpId`. This `id` may be used within a **SpatialSymbolReference** object that is extended from an SBML core **Parameter** and can be used in an expression. The mathematical value of a **Domain** is the absolute size of that domain as used by the simulator (the meshed size), and can be bound to a **Parameter** by using a **SpatialSymbolReference** to the **SpatialSymbolReference** id. The optional `name` attribute is of type `string`, may be used to add a human-readable label to the object, and has no uniqueness requirements.

As a derived quantity, if connected to a **Parameter** via a **SpatialSymbolReference**, this value may *not* be overridden by an **InitialAssignment**, nor by the use of a **Rule** or **Event**. Its value is always connected to the size of the corresponding **Geometry** instead. The units of the **Domain** are the same as the units of the corresponding **DomainType**.

#### *3.20.2 The `domainType` attribute*

The `domainType` attribute refers to the `SpId` of the **DomainType** that describes the anatomy and physiology of this domain. The attribute is of type `SpIdRef`. It is through this association that compartments, and hence the whole SBML model, gets mapped to the individual domains.

### 3.21 The InteriorPoint class

Each **Domain** can contain a **ListOfInteriorPoints**. The list of spatial points for a domain is interior to that domain. This list is optional for a **Domain** if it is the only **Domain** defined for its **DomainType**, but is required otherwise.

For those geometric descriptions that can describe multiple disjoint domains belonging to the same `domainType`, these interior points allow unambiguous identification of each domain. Formally, a single point would suffice, but in practice some tools (e.g. Smoldyn) require multiple points to handle non-convex volumes bounded by explicit surfaces. For discontinuous surfaces with the same `domainType`, the interior point identifies which domain is associated with which surface patch defined in the geometry definition.

Each **InteriorPoint** has three attributes: `coord1`, `coord2`, and `coord3`.

#### *3.21.1 The `coord1`, `coord2`, and `coord3` attributes*

An InteriorPoint element represents a single point within the defined coordinate system and should be in the interior of the domain that contains it. It has three attributes, `coord1`, `coord2`, and `coord3`, of type `double`, representing the position along each of the up to three coordinate axes defined by the **CoordinateComponents** (with `type` "`cartesianX`", "`cartesianY`", and "`cartesianZ`", respectively, for each `coord_` attribute; see Table 1 on page 20).

Each **InteriorPoint** must define the same number of attributes as there are dimensions of the corresponding **Geometry** to which it belongs.

In the case of surfaces, interior points are sometimes required to make unambiguous identification of multiple surfaces (e.g multiple plasma membranes for multiple cells present in a geometry). Due to roundoff error and finite word lengths, it is difficult to find a three dimensional point that lies on a surface. In this case, the distance from the surface will be used to provide unambiguous identification.

### 3.22 The AdjacentDomains class

**AdjacentDomains** (or domain adjacencies) captures the topological relationships within the **Geometry**. Consider that the **Domains** are nodes in a graph. The **AdjacentDomains** objects are the edges that specify the spatial connectivity of these nodes. Armed with the topology and the domain sizes, one can readily perform a compartmental approximation. Figure 11 on the next page shows the definition of the **AdjacentDomains** object.

#### *3.22.1 The `id` and `name` attributes*

This attribute identifies an **AdjacentDomains** object. The attribute is of type `SpId`. It has no mathematical meaning, and cannot be connected to a **Parameter** via a **SpatialSymbolReference** element. The optional `name` attribute is of type `string`, may be used to add a human-readable label to the object, and has no uniqueness requirements.

#### *3.22.2 The `domain1` and `domain2` attributes*

The `domain1` and `domain2` attributes, of type `SpIdRef`, are required attributes. They are the `SpId`'s of two domains that touch each other (spatially adjacent). These are typically surface-volume contacts.

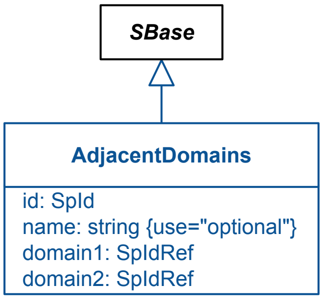

***Figure 11:*** *The definition of the **AdjacentDomains** class. **Geometry** can contain one instance of **ListOfAdjacentDomains** that can have one or more instances of **AdjacentDomains** objects.*

### 3.23 Domain example

The following is an example of a set of **Domain**, **DomainType**, and **AdjacentDomains** objects.

```
<spatial:listOfDomainTypes>
  <spatial:domainType spatial:id="Cytosol" spatial:spatialDimensions="3"/>
  <spatial:domainType spatial:id="Nucleus" spatial:spatialDimensions="3"/>
</spatial:listOfDomainTypes>
<spatial:listOfDomains>
  <spatial:domain spatial:id="Cytosol0" spatial:domainType="Cytosol">
    <spatial:listOfInteriorPoints>
      <spatial:interiorPoint spatial:coord1="2.4" spatial:coord2="2.4" spatial:coord3="0.5"/>
    </spatial:listOfInteriorPoints>
  </spatial:domain>
  <spatial:domain spatial:id="Nucleus0" spatial:domainType="Nucleus">
    <spatial:listOfInteriorPoints>
      <spatial:interiorPoint spatial:coord1="4.8" spatial:coord2="4.8" spatial:coord3="0.5"/>
    </spatial:listOfInteriorPoints>
  </spatial:domain>
</spatial:listOfDomains>
<spatial:listOfAdjacentDomains>
  <spatial:adjacentDomains spatial:domain1="Cytosol0" spatial:domain2="Nucleus0"
                           spatial:id="Nucleus_Cytosol"/>
</spatial:listOfAdjacentDomains>
```

### 3.24 The GeometryDefinition class

A **Geometry** can specify a list of ***GeometryDefinitions***. The ***GeometryDefinition*** is an abstract class that is the general term for the container which defines the concrete geometric constructs represented by the **Geometry**. Four types of ***GeometryDefinitions*** have been identified - **AnalyticGeometry**, **SampledFieldGeometry**, **ParametricGeometry**, **CSGeometry** (Constructed Solid Geometry). In addition, a **MixedGeometry** may combine elements of any of the other four basic types. These geometries are elaborated in the following sections. The definition of the ***GeometryDefinition*** element is displayed in Figure 12 on the following page. The spatial dimension of the ***GeometryDefinition*** must match the `spatialDimensions` of the **DomainType** defined for the associated **Domain**.

#### *3.24.1 The `id` and `name` attributes*

The `id` attribute is common to all the ***GeometryDefinition*** types and is used to uniquely identify the ***GeometryDefinition***. The attribute is of type `SpId`. It has no mathematical meaning, and cannot be connected to a **Parameter** via a **SpatialSymbolReference** element. The optional `name` attribute is of type `string`, may be used to add a human-readable label to the object, and has no uniqueness requirements.

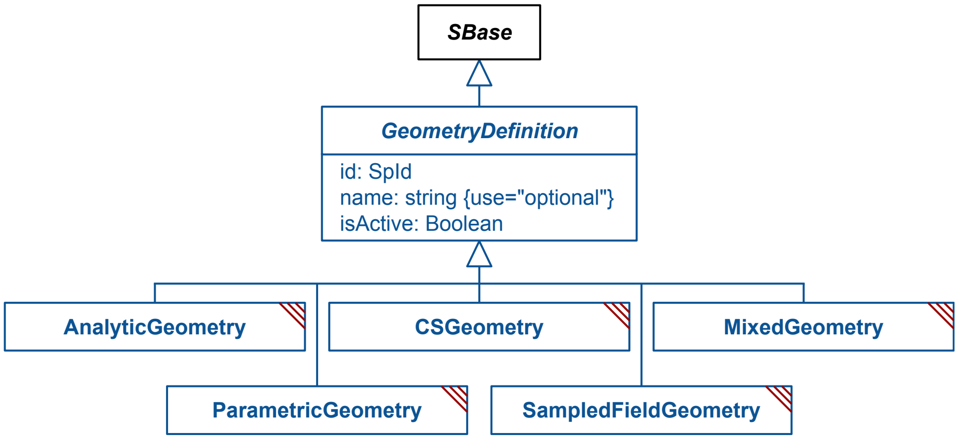

***Figure 12:*** *The **GeometryDefinition** element. **Geometry** contains one instance of listOfGeometryDefinitions that can contain one or more instances of **GeometryDefinition** (one of **AnalyticGeometry**, **SampledFieldGeometry**, **CSGeometry**, **ParametricGeometry**, or **MixedGeometry**, defined below).*

#### *3.24.2 The `isActive` attribute*

The `isActive` attribute that is common to all the ***GeometryDefinition*** types is used to identify the ***GeometryDefinition*** that is considered the active ***GeometryDefinition*** for the document. When multiple ***GeometryDefinition*** elements define the same underlying geometry, each may set their `isActive` attribute to “`true`”. At least one ***GeometryDefinition*** in a **Model** must have an `isActive` attribute of “`true`”, and any other ***GeometryDefinition*** that does not describe that same underlying physical geometry must have an `isActive` value of “`false`”.

### 3.25 The AnalyticGeometry class

The **AnalyticGeometry** is a class of ***GeometryDefinition*** where the geometry of each domain is defined by an analytic expression. An **AnalyticGeometry** is defined as a collection of **AnalyticVolumes**, one **AnalyticVolume** for each volumetric domain in the geometry. In this representation, the surfaces are treated as the boundaries between dissimilar **AnalyticVolumes**. The **AnalyticGeometry** object contains a **ListOfAnalyticVolumes**. Figure 13 on the next page shows the definition of the **AnalyticGeometry** object.

### 3.26 The AnalyticVolume class

The **AnalyticVolume** is used to specify the analytic expression of a domain. The analytic expression for the **AnalyticVolume** is defined in the Math element. Despite the word 'volume' in the name, an **AnalyticVolume** may be defined for geometries of any dimension. The only difference is that the **Math** of an **AnalyticVolume** for a 3-dimensional geometry will contain references to the three **CoordinateComponent** axes (i.e. “`x`”, “`y`”, and “`z`”), but will contain fewer for lower-dimensional geometries.

#### *3.26.1 The `id` and `name` attributes*

The `id` attribute uniquely identifies the **AnalyticVolume**. The attribute is required and is of type `SpId`. It has no mathematical meaning, and cannot be connected to a **Parameter** via a **SpatialSymbolReference** element. The optional `name` attribute is of type `string`, may be used to add a human-readable label to the object, and has no uniqueness requirements.

#### *3.26.2 The `functionType` attribute*

The `functionType` attribute is of type `FunctionKind` and is currently limited to just “`layered`” (a possibility for future versions of the specification is to allow the value “`R-function`”). A “`layered`” function type implies that

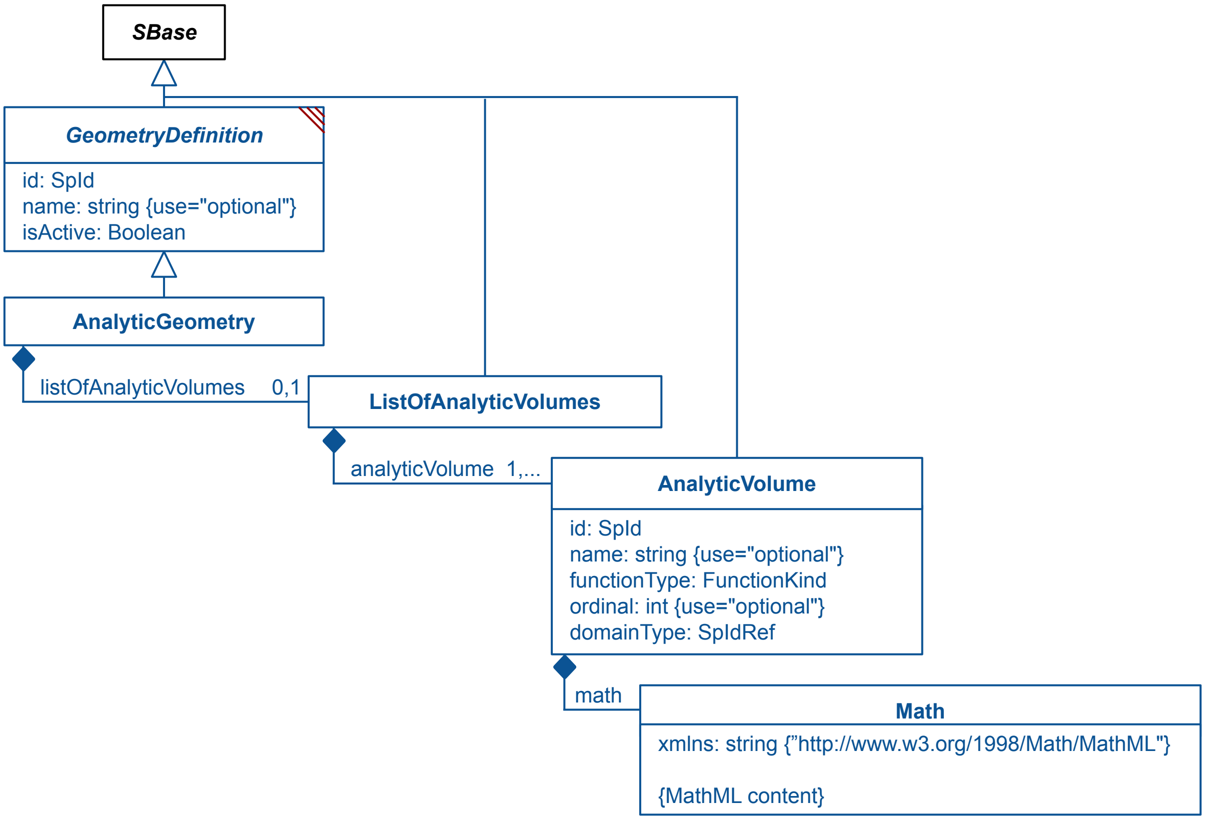

***Figure 13:*** *The definition of the **AnalyticGeometry**, **ListOfAnalyticVolumes**, and **AnalyticVolume** classes.*

the Math child element contains an inequality in the spatial dimensions (e.g. x,y,z) such that evaluation to "`true`" indicates that the point (x,y,z) is within that shape, and "false" indicates that it is not covered by that shape.

#### 3.26.3 The `domainType` attribute

The `domainType` attribute of type `SpIdRef` is a required attribute. It represents the `SpId` of the **DomainType** of the **Domain** that is represented by this **AnalyticVolume**.

#### 3.26.4 The `ordinal` attribute

The `ordinal` attribute is of type `int`, and is required. It is used to represent the order of the **AnalyticVolume**. The `ordinal` is useful while reconstructing the geometry in the specific software tool - it represents the order in which the **AnalyticVolumes** representing geometric domains have to be evaluated.

Rather than struggle with the task of preventing overlapping regions of space from different **AnalyticVolumes**, the **AnalyticVolumes** are to be considered to be evaluated in the reverse order of their ordinals. In this way, any **AnalyticVolumes** that have already been processed will cover those with a smaller ordinal, thus resolving any ambiguities and removing the constraint that all **AnalyticVolumes** be disjoint and cover the entire geometric domain. The **AnalyticVolume** with `ordinal` 0 can be the "background" layer (typically the extracellular space).

No two **AnalyticVolume** elements should have the same `ordinal` value, even if they should not overlap, because some tools may not calculate the geometries to the same level of precision as other tools, and may end up with overlap due to rounding errors, and will still need to resolve the ambiguity for their own purposes. If a software tool discovers two overlapping **AnalyticVolume** elements with the same `ordinal` value, it may resolve the situation however it sees fit.

### 3.27 The Math class

The Math element is a required element for an **AnalyticVolume**. The Math element contains a MathML expression that defines the analytic expression for the **AnalyticVolume** referencing the coordinate components that are specified in the **ListOfCoordinateComponents** in the **Geometry**, according to the `functionType`.

### 3.28 AnalyticGeometry example

The following is an example of an analytic geometry, with a single volume described by the formula "$8*(x-1)^2+8*(y-1)^2+8*(z-1)^2<1$" (the formula for a sphere).

```
<spatial:analyticGeometry spatial:id="spatial3d_spheres" spatial:isActive="true">
  <spatial:listOfAnalyticVolumes>
    <spatial:analyticVolume spatial:id="Nucleus1" spatial:functionType="layered" spatial:ordinal="2"
                            spatial:domainType="Nucleus">
      <math xmlns="http://www.w3.org/1998/Math/MathML">
        <apply> <lt/> <apply> <plus/> <apply> <times/> <cn>8</cn> <apply> <power/> <apply> <minus/>
            <ci>x</ci> <cn>1</cn> </apply> <cn>2</cn> </apply> </apply> <apply> <times/>
            <cn>8</cn> <apply> <power/> <apply> <minus/> <ci>y</ci> <cn>1</cn> </apply> <cn>2</cn>
            </apply> </apply> <apply> <times/> <cn>8</cn> <apply> <power/> <apply> <minus/>
            <ci>z</ci> <cn>1</cn> </apply> <cn>2</cn> </apply> </apply> </apply> <cn>1</cn>
        </apply>
      </math>
    </spatial:analyticVolume>
  </spatial:listOfAnalyticVolumes>
</spatial:analyticGeometry>
```

### 3.29 The SampledFieldGeometry class

**SampledFieldGeometry** is a type of ***GeometryDefinition*** that defines a sampled image-based geometry or a geometry based on samples from a level set. **SampledFieldGeometry** is defined by referencing a **SampledField** from the **ListOfSampledFields** on the **Geometry** element that specifies the sampled image, together with a a list of **SampledVolumes** that represent the volumetric domains as sampled image regions. Figure 14 on the following page shows the definition of the **SampledFieldGeometry** object. It may be used for geometries of any dimension.

#### *3.29.1 The* `sampledField` *attribute*

The `sampledField` attribute is of type SpIdRef, and must reference a **SampledField** from the same **Geometry**. That referenced field is to be used to set up the different spatial areas in the geometry of the **Model**, according to the **SampledVolume** child elements.

### 3.30 The SampledVolume class

A **SampledVolume** represents an interval of the sampled field that constitutes one or more contiguous regions. A **SampledVolume** is defined for each volumetric (3-dimensional) **Domain** in the **Geometry**. Volumes are defined as regions within the referenced image that match a particular pixel value (`sampledValue`) or that match a range of pixel values (`minValue` and `maxValue`). A given **SampledVolume** must define for itself either a single value or a range of values to which it applies, but not both. Within a **ListOfSampledVolumes**, there must be at most one **SampledVolume** that corresponds to any given pixel value. That is, any given pixel value may only appear as the `sampledVolume` of a single **SampledVolume**, or be between the `minValue` and `maxValue` (inclusive) of a single **SampledVolume**. It has the following attributes:

#### *3.30.1 The* `id` *and* `name` *attributes*

The `id` attribute identifies a **SampledVolume** object. The attribute is of type `SpId` and is required when specifying a **SampledVolume**. It has no mathematical meaning, and cannot be connected to a **Parameter** via a **SpatialSymbolRef-**

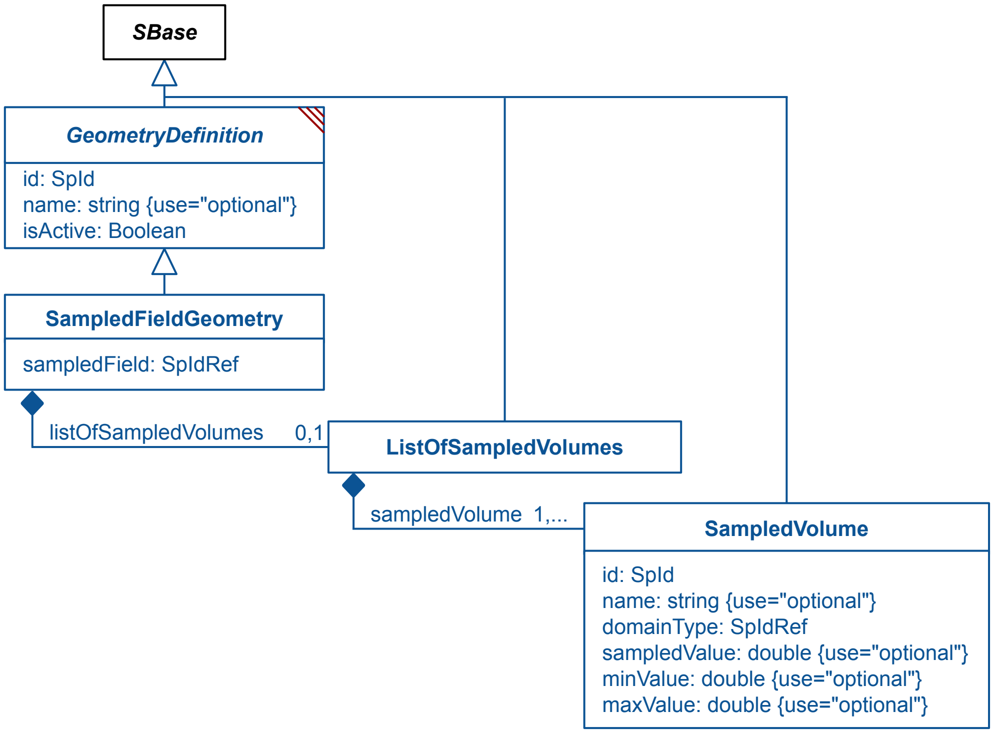

***Figure 14:*** *The definition of the* ***SampledFieldGeometry****,* ***ListOfSampledVolumes****, and* ***SampledVolume*** *classes.*

**erence** element. The optional `name` attribute is of type `string`, may be used to add a human-readable label to the object, and has no uniqueness requirements.

#### 3.30.2 The `domainType` attribute

The required `domainType` attribute is of type `SpIdRef`. It is the `SpId` of the **DomainType** that represents this class of anatomical features. If there are more than one contiguous regions, then more than one domain will be defined corresponding to each **SampledVolume**.

#### 3.30.3 The `sampledValue` attribute

The optional `sampledValue` attribute is of type `double`. It represents the pixel value of a **SampledVolume**. When used, all pixels with this particular value are to be assigned to this **SampledVolume**, and no pixel without this value is assigned to the **SampledVolume**. It is illegal to define a `sampledValue` attribute for a **SampledVolume** that also has a `minValue` and `maxValue` defined.

#### 3.30.4 The `minValue` attribute

The optional `minValue` attribute is of type `double`. It represents the minimum of the pixel value (`sampledValue`) range to be assigned to this **SampledVolume**. If a `minValue` attribute is defined, a `maxValue` attribute must also be defined, and the `sampledValue` attribute must not be defined. Areas in the **SampledField** with *exactly* this value are to be included in this **SampledVolume**.

#### 3.30.5 The `maxValue` attribute

The optional `maxValue` attribute is of type `double`. It represents the maximum of the pixel value (`sampledValue`) range to be assigned to this **SampledVolume**. If a `maxValue` attribute is defined, a `minValue` attribute must also be defined, and the `sampledValue` attribute must not be defined. Areas in the **SampledField** with *exactly* this value are to be excluded from this **SampledVolume**.

Having the `minValue` be included and the `maxValue` be excluded allows modelers to define adjacent volumes without ambiguity at the boundaries. One **SampledVolume** may be defined from 0 to 100, and a second volume defined from 100 to 200, and any location with a **SampledField** value of exactly 100 is only assigned to the first **SampledVolume**, and every location with a value close to 100 is included in exactly one **SampledVolume**.

### 3.31 SampledFieldGeometry example

The following is an example of a sampled field geometry with three volumes. The referenced **SampledField** (defined in Section 3.16 on page 20) is also included (though truncated).

```
<spatial:listOfGeometryDefinitions>
  <spatial:sampledFieldGeometry spatial:isActive="true" spatial:sampledField="imgvals">
    <spatial:listOfSampledVolumes>
      <spatial:sampledVolume spatial:id="Extracellular2" spatial:domainType="Extracellular"
                             spatial:minValue="0" spatial:maxValue="64"/>
      <spatial:sampledVolume spatial:id="Cytosol2" spatial:domainType="Cytosol2"
                             spatial:minValue="64" spatial:maxValue="192"/>
      <spatial:sampledVolume spatial:id="Nucleus2" spatial:domainType="Nucleus"
                             spatial:minValue="192" spatial:maxValue="256"/>
    </spatial:listOfSampledVolumes>
  </spatial:sampledFieldGeometry>
</spatial:listOfGeometryDefinitions>
<spatial:listOfSampledFields>
  <spatial:sampledField spatial:id="imgvals" spatial:dataType="uint8" spatial:numSamples1="51"
                        spatial:numSamples2="59" spatial:numSamples3="23"
                        spatial:interpolationType="linear" spatial:compression="uncompressed"
                        spatial:samplesLength="69207">
                              120 218 237 [...] 131 28 215
  </spatial:sampledField>
</spatial:listOfSampledFields>
```

### 3.32 The CSGeometry class

**CSGeometry** (Constructed Solid Geometry) is a type of ***GeometryDefinition*** that defines a combined, solid, volumetric object from a number of primitive solid volumes by the application of set operations such as union, intersection and difference and affine transformations such as rotation, scaling, translation, etc. The **CSGeometry** element is defined by a `listOfCSGObjects` element that contains a collection of **CSGObjects**. Figure 15 on the following page shows the definition of the **CSGeometry** object.

### 3.33 The CSGObject class

Each **CSGObject** is a scene graph representing a particular geometric object using constructed solid geometry. A node in a tree (scene graph) is made up of **CSGPrimitives**, **CSGSetOperators**, and ***CSGTransformations***. Note that the **CSGPrimitives** are always leaves in this tree. The **CSGObject** is analogous to an **AnalyticVolume** element in the sense that it is a constructed geometry (from primitives) used to specify a volumetric (3-dimensional) domain. The **CSGObject** element has three attributes : `id`, `domainType` and `ordinal`. The definition of the **CSGObject** is completed by defining a ***CSGNode*** which is the root of the **CSGObject** scene graph.

#### *3.33.1 The `id` and `name` attributes*

The `id` attribute uniquely identifies the **CSGObject** element. The attribute is required and is of type `SpId`. It has no mathematical meaning, and cannot be connected to a **Parameter** via a **SpatialSymbolReference** element. The optional `name` attribute is of type `string`, may be used to add a human-readable label to the object, and has no uniqueness requirements.

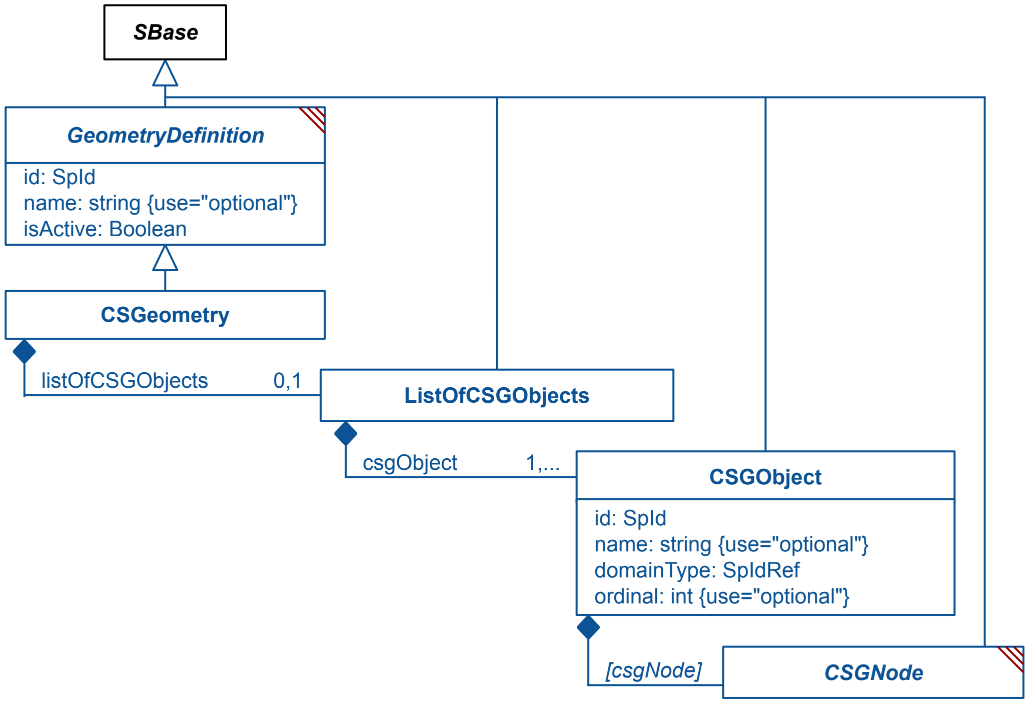

***Figure 15:*** *The definition of the **CSGeometry**, **ListOfCSGObjects**, and **CSGObject** classes.*

#### *3.33.2 The* `domainType` *attribute*

The `domainType` attribute is of type `SpIdRef` and is a required attribute. It is a reference to the `id` of the **DomainType** that this **CSGObject** represents.

All **InteriorPoints** of the corresponding **DomainType** must be points inside the geometry this **CSGObject** describes.

#### *3.33.3 The* `ordinal` *attribute*

The `ordinal` attribute is of type `int`. It is used to represent the order of the **CSGObject**. The `ordinal` is useful while reconstructing the geometry in the specific software tool - it represents the order in which the **CSGObjects** representing geometric domains have to be placed.

No two **CSGObject** elements should have the same `ordinal` value, even if they should not overlap, because some tools may not calculate the geometries to the same level of precision as other tools, and may end up with overlap due to rounding errors, and will still need to resolve the ambiguity for their own purposes. If a software tool discovers two overlapping **CSGObject** elements with the same `ordinal` value, it may resolve the situation however it sees fit.

#### *3.33.4 The* [`csgNode`] *child*

The child [`csgNode`] element represents the geometry that is to be linked to the `domainType` of the **CSGObject**. Note that the child of the **CSGObject** element is not called “`csgNode`” but rather takes the name of the derived class, decapitalized. Thus, a **CSGObject** may have a `csgPrimitive`, `csgSetOperator`, `csgTranslation`, `csgRotation`, `csgScale`, or `csgHomogeneousTransformation` child.

### 3.34 The CSGNode class

The operators and operands used to construct a constructed solid geometry are generalized as a ***CSGNode***, defined in Figure 16 on the next page as an abstract base class. The classes that inherit from ***CSGNode*** can be one of the following: **CSGSetOperator**, ***CSGTransformation*** (operators; itself another abstract base class), or **CSGPrimitive**

(operands). The ***CSGNode*** has one attribute: `id`. The **CSGObject** contains a ***CSGNode*** object which is the root of the **CSGObject** scene graph (representing one constructed solid geometry domain).

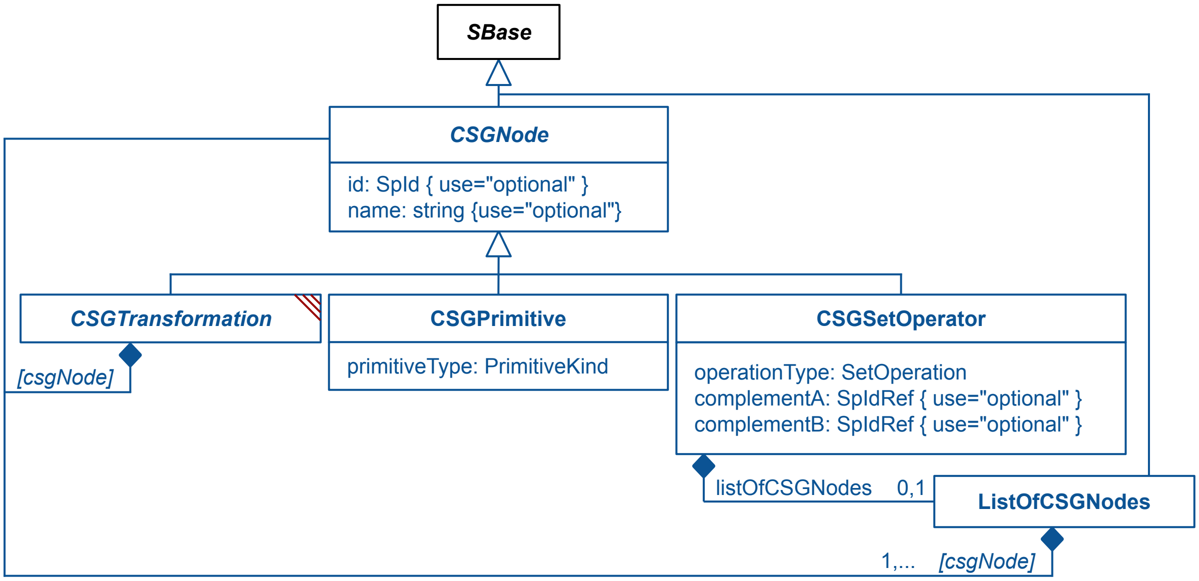

***Figure 16:*** *The definition of the abstract base class* ***CSGNode****, and its subclasses* ***CSGPrimitive****, and* ***CSGSetOperator****. The abstract base class* ***CSGTransformation*** *(also a subclass of* ***CSGNode****) is defined in* *Section 3.38 on the next page**.*

#### *3.34.1 The* `id` *and* `name` *attributes*

The `id` attribute uniquely identifies the ***CSGNode*** element. The attribute is optional and is of type `SpId`. It has no mathematical meaning, and cannot be connected to a **Parameter** via a **SpatialSymbolReference** element. The optional `name` attribute is of type `string`, may be used to add a human-readable label to the object, and has no uniqueness requirements.

### 3.35 The CSGPrimitive class

**CSGPrimitive** element represents the primitive geometric shapes that can be represented by the **CSGeometry**. These shapes are defined in Table 2 on the following page with a predefined orientation and fitting within the unit cube (+/- 1 in x, y, and z) or unit square (+/- 1 in x and y). This element has one required attribute : `primitiveType` of type `PrimitiveKind`.

#### *3.35.1 The* `primitiveType` *attribute*

The `primitiveType` attribute is a required attribute that is of type `PrimitiveKind`. It represents one of the predefined primitive shapes. The meaning and definition of those types is listed in Table 2 on the next page.

### 3.36 The CSGSetOperator class

The **CSGSetOperator** element represents the set operations (union, intersection, difference) that can be performed on a set of primitive geometric shapes (**CSGPrimitives**) or on a set of ***CSGNodes*** (a transformation or set operation on one or a set of **CSGPrimitives**). This element has one attribute of type `string`. It also contains a required child **ListOfCSGNodes** that represents the set of nodes on which the set operation is performed.

| PrimitiveKind | Dimensions | Definition |
|---|---|---|
| sphere | 3 | A sphere with radius 1, centered at the origin. |
| cube | 3 | A cube with sides of length 2, centered at the origin. |
| cylinder | 3 | A cylinder with a base circle of radius 1, centered at (0,0,-1), and top circle of radius 1, centered at (0,0,1). The height is 2. |
| cone | 3 | A cone with a base circle of radius 1, centered at (0,0,-1), and top vertex at (0,0,1). |
| circle | 2 | A circle with radius 1, centered at the origin. |
| square | 2 | A square with sides of length 2, centered at the origin |

***Table 2:*** *Definitions of the possible values of the* `primitiveType` *attribute of the* ***CSGPrimitive*** *class.*

#### *3.36.1 The* `operationType` *attribute*

The `operationType` attribute is of type `SetOperation` and represents an operation that can be performed on a set of ***CSGNodes***. The possible values that the `operationType` attribute can take are "`union`", "`intersection`", or "`difference`". The values "`union`" and "`intersection`" are n-ary, meaning they are defined for any number of child nodes of this **CSGSetOperator**. The intersection or union of the empty set (zero children) is defined as the empty set, and the intersection or union of a single child is defined as that child. The union of multiple sets is defined as including any element that appears in any of those component sets, and the intersection of multiple sets is defined as including only those elements that appear in all of the component sets.

The value "`difference`" is binary, meaning that it must have exactly two children. Its meaning is defined according to the `complement` attributes, below.

#### *3.36.2 The* `complementA` *and* `complementB` *attributes*

The `complement` attributes are of type `SpIdRef`. If the `operationType` of the **CSGSetOperator** has the value "`difference`", they both must be set to indicate the order in which the complement is to be carried out, and must refer, respectively, to the two `csgNode` children of this **CSGSetOperator**. The relative complement of the children (and thus the meaning of this node) is defined as the set of elements in `complementB`, but not in `complementA`.

If the `operationType` of the **CSGSetOperator** is not "`difference`", neither `complement` attribute may be set.

### 3.37 The ListOfCSGNodes class

The **ListOfCSGNodes** must contain one or more `csgNode` children that are to be combined according to the set operation of the parent **CSGSetOperator**. While having a single child is legal, this is semantically equivalent to simply putting that child in the model instead of the **CSGSetOperator**, and therefore has limited modeling benefit. Note that the children of the **ListOfCSGNodes** object are not called `csgNode` but rather take the name of the derived class, decapitalized. Thus, they may be called `csgPrimitive`, `csgSetOperator`, `csgTranslation`, `csgRotation`, `csgScale`, or `csgHomogeneousTransformation`.

### 3.38 The CSGTransformation class

The ***CSGTransformation*** represents a generalization for the type of transformation that can be performed on a primitive geometric shape (**CSGPrimitive**) or on a ***CSGNode*** (a transformation or set operation on one or a set of **CSGPrimitives**). The types of possible transformations are 'rotation', 'translation', 'scaling', and 'homogeneous transformation', defined below. The ***CSGTransformation*** element contains a ***CSGNode*** element upon which the transformation is performed.

Each transformation is performed directly on its child ***CSGNode***, with any transformation or set operation from that node already performed. This essentially defines a 'bottom-up' approach, where the tips of the XML tree children of a **CSGObject** are all **CSGPrimitive** objects, that are progressively transformed or combined with other **CSGPrimitive** objects by each successive node moving rootward through the XML. For an example, see Section 3.44 on page 39.

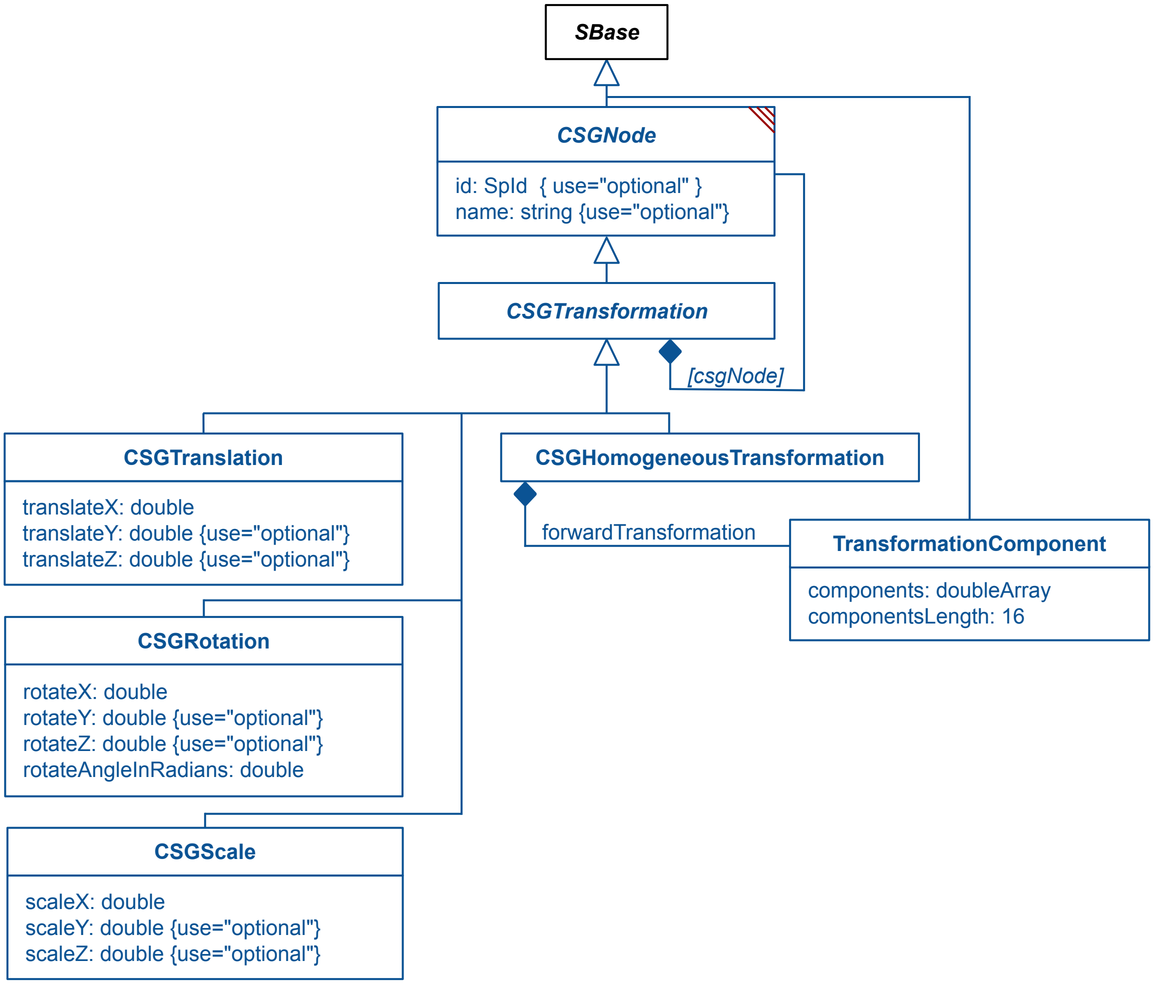

***Figure 17:*** *The definition of the abstract base class* ***CSGTransformation****, its subclasses (****CSGTranslation****,* ***CSGRotation****,* ***CSGScale****, and* ***CSGHomogeneousTransformation****), and the* ***TransformationComponent*** *class.*

#### *3.38.1 The `csgNode` child*

The child `csgNode` element represents the geometry that is to be transformed by the ***CSGTransformation*** element. Note that this child is not called `csgNode` but rather takes the name of the derived class, decapitalized. Thus, it may be called `csgPrimitive`, `csgSetOperator`, `csgTranslation`, `csgRotation`, `csgScale`, or `csgHomogeneousTransformation`.

### 3.39 The CSGTranslation class

The **CSGTranslation** element represents a translation transformation on a ***CSGNode*** (a transformation or set operation on one or a set of **CSGPrimitives**) or a **CSGPrimitive** along the axes defined in the **Geometry**. This element has three attributes:

#### *3.39.1 The `translateX` attribute*

The `translateX` required attribute is of type `double`. It represents the translation of the ***CSGNode*** along the x-axis (the **CoordinateComponent** with the `type` of "`cartesianX`").

#### 3.39.2 The translateY attribute

The translateY attribute is of type double. It represents the translation of the *CSGNode* along the y-axis (the CoordinateComponent with the type of "cartesianY"). This attribute must not be defined if no such CoordinateComponent is present in the parent Geometry, and is required otherwise.

#### 3.39.3 The translateZ attribute

The translateZ attribute is of type double. It represents the translation of the *CSGNode* along the z-axis (the CoordinateComponent with the type of "cartesianZ"). This attribute must not be defined if no such CoordinateComponent is present in the parent Geometry, and is required otherwise.

### 3.40 The CSGRotation class

The CSGRotation element represents a rotation transformation on a *CSGNode* (a transformation or set operation on one or a set of CSGPrimitives) or a CSGPrimitive about the axes defined in the Geometry. This element has 4 attributes:

#### 3.40.1 The rotateX, rotateY, and rotateZ attributes

The rotate attributes are of type double, and must be defined for the same number of CoordinateComponents as are present in the parent Geometry. If all three are defined, they define a point in space that determines the vector (from the origin) of the axis about which the rotation is to occur in three-dimensional space. They must therefore not all be equal to zero. If two are defined (rotateX and rotateY), they define the point in two-dimensional space about which the rotation is to occur. (In this case, (0,0) would be legal.) Nothing can be rotated in 1-dimensional space, and therefore defining only rotateX is meaningless.

#### 3.40.2 The rotateAngleInRadians attribute

The rotateAngleInRadians attribute is of type double. It represents the rotation angle of the *CSGNode*, in radians, along the defined axis. In three-dimensional space, this rotation is defined as counterclockwise from the perspective of the origin, viewing the constructed vector. In two-dimensional space, this rotation is defined the same way, as a counterclockwise rotation along the Z axis emerging from the defined point. From the perspective of the surface of the shape, this view looks down the negative side of the Z axis, appearing as a clockwise rotation.

Overall, the rotate attributes define *where* the rotation is to occur, and the rotateAngleInRadians define *how much* rotation is to take place. A rotateAngleInRadians value of "0" would therefore define a CSGRotation that left the shape fixed in space.

### 3.41 The CSGScale class

The CSGScale element represents a scale transformation on a *CSGNode* (a transformation or set operation on one or a set of CSGPrimitives) or a CSGPrimitive along the axes defined in the Geometry. All scaling occurs collectively for the component primitive shapes, and the expansion occurs from the geometrical center of the object, i.e. the center of the smallest bounding box that would contain the current volume of the object. This means, for example, that if the child of the CSGScale object is a hemisphere, defined as the intersection of a sphere and a cube, the bounding box would be half the size of a box that would have included the original entire sphere.

This element has three attributes:

#### 3.41.1 The scaleX attribute

The scaleX required attribute is of type double. It represents the amount of scaling of the *CSGNode* along the x-axis (the CoordinateComponent with the type of "cartesianX").

#### *3.41.2 The `scaleY` attribute*

The `scaleY` attribute is of type `double`. It represents the amount of scaling of the ***CSGNode*** along the y-axis (the **CoordinateComponent** with the `type` of "`cartesianY`"). This attribute must not be defined if no such **CoordinateComponent** is present in the parent **Geometry**, and is required otherwise.

#### *3.41.3 The `scaleZ` attribute*

The `scaleZ` attribute is of type `double`. It represents the amount of scaling of the ***CSGNode*** along the z-axis (the **CoordinateComponent** with the `type` of "`cartesianZ`"). This attribute must not be defined if no such **CoordinateComponent** is present in the parent **Geometry**, and is required otherwise.

The amount of scaling for all three attributes is relative to the original size of the object. In other words, if a **CSGScale** object has a `scaleX` value of "`1.0`", is it not scaled along the X axis at all, with "`0.5`" halving it, "`2.0`" doubling it, and "`0.0`" making the entire object disappear completely. Negative values produce effective inversions of the object, e.g. inverting a unit cone by giving it a `scaleZ` scaling factor of "`-1`".

### 3.42 The CSGHomogeneousTransformation class

The **CSGHomogeneousTransformation** element represents a homogeneous transformation on a ***CSGNode***: a transformation or set operation on one or more **CSGPrimitives**. This element contains one required **TransformationComponent** element child named `forwardTransformation`.

### 3.43 The TransformationComponent class

The **TransformationComponent** element represents an affine transformation that can be applied to a ***CSGNode***. This element has the following two attributes:

#### *3.43.1 The `components` attribute*

The `components` attribute is of type `doubleArray`, whose values represent the 4x4 affine transformation matrix. This attribute is required.

An affine transformation is essentially a method to transform a shape's scale, rotation, and translation all at once instead of breaking it down into its component tranformations. For one description of how to transform a model with an affine transformation, see the OpenGL Programming Guide, Chapter 5 (Shreiner et al., 2013).

#### *3.43.2 The `componentsLength` attribute*

The `componentsLength` attribute is of type `int`, is required, and must be 16. It represents the array length of the `components` attribute (number of values in the `components` array), which must be a 4x4 matrix.

### 3.44 CSGObject examples

The following is an example of a **CSGObject** which has been scaled, rotated, and translated:

```
<spatial:csgObject spatial:id="vesicleObj" spatial:domainType="vesicle"
                   spatial:ordinal="2">
  <spatial:csgTranslation spatial:id="translation1" spatial:translateX="5.439"
                          spatial:translateY="5.88" spatial:translateZ="1.078">
    <spatial:csgRotation  spatial:id="rotation1" spatial:rotateX="2.4391"
                          spatial:rotateY="1.5373" spatial:rotateZ="0.58404"
                          spatial:rotateAngleInRadians="0.9104">
      <spatial:csgScale   spatial:id="scale1" spatial:scaleX="0.075833"
                          spatial:scaleY="0.080451" spatial:scaleZ="0.029583">
        <spatial:csgPrimitive spatial:id="sphere1" spatial:primitiveType="sphere"/>
     </spatial:csgScale>
    </spatial:csgRotation>
```

```
    </spatial:csgTranslation>
  </spatial:csgObject>
```

The manipulations can be read from the inside out: at the base level, we have the **CSGPrimitive** sphere: a sphere with radius 1.0, centered at the origin. It's then scaled such that it is compressed along each axis by different amounts: along the X axis to 7.5833%, along the Y axis to 8.0451%, and along the Z axis to 2.9583%. Then the scaled figure is rotated around a vector with its origin at (0,0,0) and its point at (2.4391, 1.5373, 0.58404), by 0.9104 radians. Finally, it is translated by 5.439 along the X axis, 5.88 along the Y axis, and 1.078 along the Z axis.

In this second example, a hemisphere is created through the intersection of a primitive sphere and translated cube:

```
  <spatial:csgObject spatial:id="hemisphere" spatial:domainType="Nucleus">
    <spatial:csgSetOperator spatial:operationType="intersection">
      <spatial:listOfCSGNodes>
        <spatial:csgPrimitive spatial:primitiveType="sphere"/>
        <spatial:csgTranslation spatial:translateX="1" spatial:translateY="0"
                                spatial:translateZ="0">
          <spatial:csgPrimitive spatial:primitiveType="cube"/>
        </spatial:csgTranslation>
      </spatial:listOfCSGNodes>
    </spatial:csgSetOperator>
  </spatial:csgObject>
```

### 3.45 The ParametricGeometry class

**ParametricGeometry** is a type of ***GeometryDefinition*** that parametrically defines geometric strucutures/domains. The **ParametricGeometry** element is defined with a **SpatialPoints** object and a `listOfParametricObjects` that is a collection of **ParametricObjects**. Each point in the **SpatialPoints** list is given an index, and those indices are used in the creation of each **ParametricObject**. There may be points whose indices are never used; this does not affect the **ParametricGeometry**. Figure 18 on the next page shows the definition of the **ParametricGeometry** object.

### 3.46 The SpatialPoints class

The **SpatialPoints** element represents the set of points to be used as vertices in the **ParametricGeometry**. In essence, the **SpatialPoints** defines a lattice on which each **ParametricObject** is to be drawn. There may be unused points in the list if no **ParametricObject** ever uses that index to draw its shape.

#### *3.46.1 The* `compression` *attribute*

The required `compression` attribute is of type `CompressionKind`. It is used to specify the compression used when encoding the data, and can have the value "`uncompressed`" if no compression was used, or "`deflated`" if the deflation algorithm was used to compress the data. The deflation compression algorithm to be used is gzip, which adds a header to the deflated data. This algorithm is freely available. The version of the data to be compressed is the string version of the values in the array, which may consist of numbers, whitespace, commas, and semicolons.

#### *3.46.2 The* `arrayDataLength` *attribute*

The `arrayDataLength` attribute is of type `int` and is required. It represents the array length of the `arrayData` text child of this node. If uncompressed, this will equal the total number of coordinates, but if compressed, this will equal the new compressed length of the array, not including any added whitespace. It is included for convenience and validation purposes.

#### *3.46.3 The* `dataType` *attribute*

The `dataType` attribute is of type `DataKind` and is optional. It is used to specify the type of the data being stored, so that the uncompressed data can be stored in an appropriate storage type. The three main value types are "`uint`"

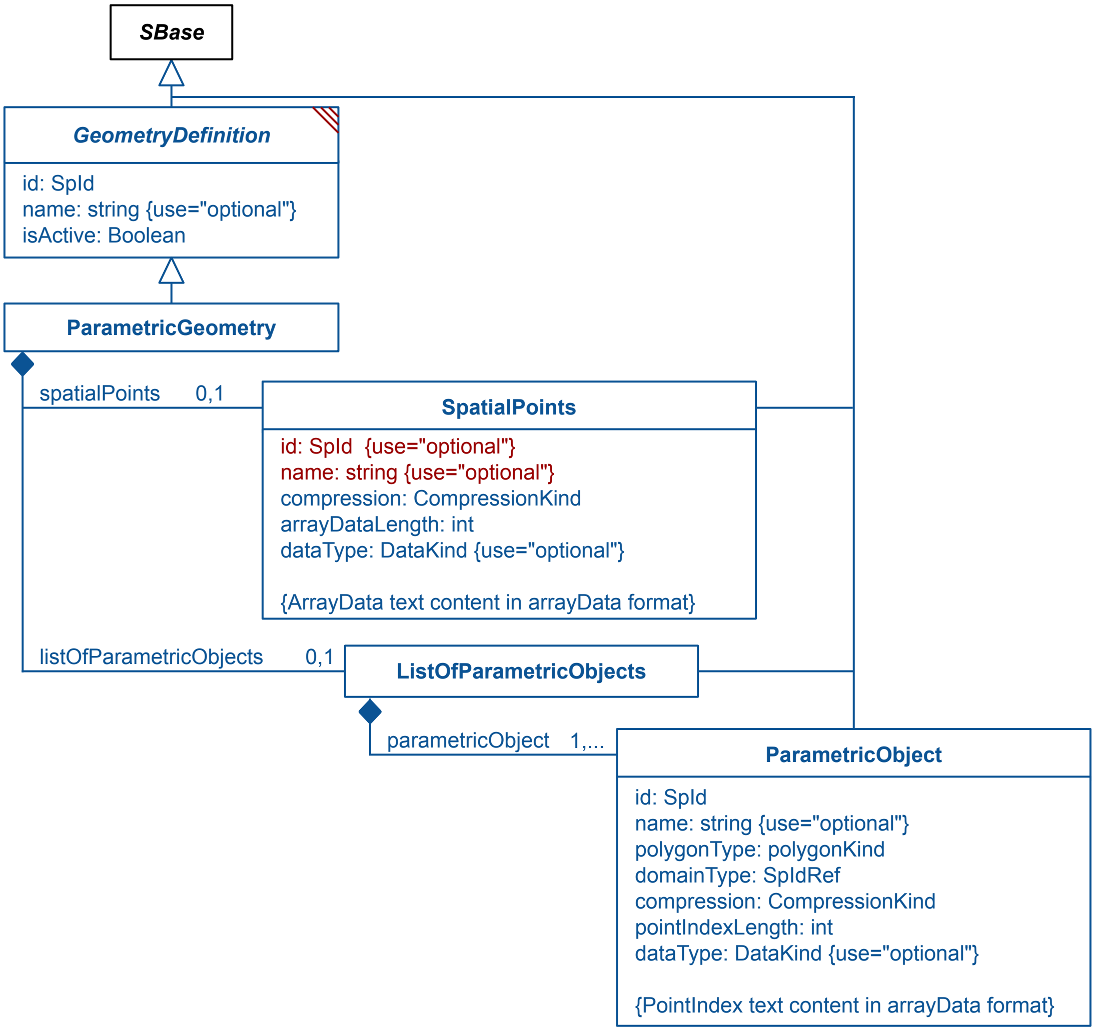

***Figure 18:*** *The definition of the* ***ParametricGeometry***, ***SpatialPoints***, ***ListOfParametricObjects***, *and* ***ParametricObject*** *classes.*

for unsigned integers, "`int`" for signed integers, and "`double`" for double-precision floating point values. For backwards compatibility, and for cases where storage space might be an issue, other values may also be used: "`float`" to indicate single-precision (32-bit) floating point values, and "`uint8`", "`uint16`", and "`uint32`" to indicate 8-bit, 16-bit, and 32-bit unsigned integer values, respectively.

#### *3.46.4 The `ArrayData` text child*

The `ArrayData` text child of the **SpatialPoints** is in `arrayData` format, and represents an ordered list of sets of coordinates that will be used as the vertices of **ParametricObject** elements in this **ParametricGeometry**, with "`0`" representing the first such coordinate, "`1`" the second, etc. The list will define vertexes with as many values as there are **CoordinateComponent** children of the parent **Geometry**: three values for representing the X, Y, and Z coordinates (respectively) of 3-dimensional geometries, or two values for representing the X and Y coordinates (respectively) of 2-dimensional geometries. (**ParametricGeometry** elements cannot be created in 1-dimensional geometries.) A semicolon may be used in uncompressed data to visually distinguish grouped values.

#### 3.46.5 The id and name attributes

Each SpatialPoints is identified with a id of type SpId. A SpatialPoints has no mathematical value. The optional name attribute is of type string, may be used to add a human-readable label to the object, and has no uniqueness requirements.

### 3.47 The ParametricObject class

The ParametricObject element represents a single parametric geometry object. It contains a list of point indices from the parent ParametricGeometry's SpatialPoints, which collectively define the faces of the object.

#### 3.47.1 The id and name attributes

The id attribute is a required attribute of type SpId. It uniquely identifies the ParametricObject element. It has no mathematical meaning, and cannot be connected to a **Parameter** via a SpatialSymbolReference element. The optional name attribute is of type string, may be used to add a human-readable label to the object, and has no uniqueness requirements.

#### 3.47.2 The polygonType attribute

The polygonType attribute is of type PolygonKind and is a required attribute. It represents the type of polygon that describes the ParametricObject. ParametricObject elements of type "triangle" have three points, and are currently the only type of polygons allowed.

#### 3.47.3 The domainType attribute

The domainType attribute is of type SpIdRef and is a required attribute. It is a reference to the id of the DomainType that this ParametricObject represents.

#### 3.47.4 The compression attribute

The required compression attribute is of type CompressionKind. It is used to specify the compression used when encoding the data, and can have the value "uncompressed" if no compression was used, or "deflated" if the deflation algorithm was used to compress the data. The deflation compression algorithm to be used is gzip, which adds a header to the deflated data. This algorithm is freely available. The version of the data to be compressed is the string version of the values in the array, which may consist of numbers, whitespace, commas, and semicolons.

#### 3.47.5 The pointIndexLength attribute

The pointIndexLength attribute is of type int and is required. It represents the array length of the arrayData text child of this node. If uncompressed, this will equal the number of referenced indices, but if compressed, this will equal the new compressed length of the array, not including any added whitespace. It is included for convenience and validation purposes.

#### 3.47.6 The dataType attribute

The dataType attribute is of type DataKind and is optional. It is used to specify the type of the data being stored, so that the uncompressed data can be stored in an appropriate storage type. Because all the data will be indexes into the ArrayData of the SpatialPoints element, "uint" is the suggested value for this attribute (indicating unsigned integer values), with "uint8", "uint16", and "uint32" (indicating 8-bit, 16-bit, and 32-bit unsigned integer values, respectively) also being allowed. No other value is allowed, including the other types of DataKind ("int", "float", and "double"), since the data will never be that type.

#### *3.47.7 The `PointIndex` text child*

The `PointIndex` text child of the **ParametricObject** is in `arrayData` format, and represents an ordered list of indices that refer to elements in the **SpatialPoints** array and are interpreted by considering the `polygonType` attribute of the **ParametricObject** with a value of “`triangle`” indicating that the data is to be grouped in sets of three. The sequence of indices must follow adjacent edges, with an implied edge between the first and last vertex. Additionally, the order of that sequence should be consistently clockwise or counter-clockwise for any contiguous surface. This can be accomplished by ensuring that when an edge is used for two different faces, the order of that edge is reversed for the second face. A semicolon may be used in uncompressed data to visually distinguish grouped values. Each set of indices that define a polygon face should each refer to a different location. This means that one should not re-use index values with a single polygon face, nor should one use two index values in the same face that are mapped to the same location.

### 3.48 A ParametricGeometry example

As an example, if the **SpatialPoints** element in a three-dimensional **Geometry** is:

```
<spatialPoints compression="uncompressed" arrayDataLength="24">
    0 0 0; 0 0 1; 0 1 0; 1 0 0; 0 1 1; 1 0 1; 1 1 0; 1 1 1
</spatialPoints>
```

This defines eight points, with point ’0’ at coordinates [0, 0, 0], point ’1’ at coordinates [0, 0, 1], etc., that happen to form the vertexes of a cube. These point indexes are then used in the following **ParametricObject**:

```
<parametricObject id="pyramid" polygonType="triangle" domainType="dt1"
                  compression="uncompressed" pointIndexLength="12">
    0 2 5; 0 6 2; 0 5 6; 2 6 5
</parametricObject>
```

This defines a pyramid with four triangular faces. The first triangle is defined by points [0,0,0], [0,1,0], and [0,1,1]; the second triangle by points [0,0,0], [1,1,0], and [0,1,0]; the third by [0,0,0], [0,1,1], and [1,1,0]; and the fourth by [0,1,0], [1,1,0], and [0,1,1]. Note also that the order of the faces is consistent, defining each face in a counter-clockwise order, as observed from ’outside’ the pyramid. This can be seen, for example, in the last two faces defined, where the shared edge is defined in the order ’5 6’ in the first face, but ’6 5’ in the second.

A more realistic example can be found below, though most of the values are elided for reasons of space:

```
<spatial:parametricGeometry spatial:id="id051101043048054" spatial:isActive="true">
  <spatial:spatialPoints spatial:compression="uncompressed" spatial:arrayDataLength="179901">
        -0.472894296 -0.4420042305 -0.457890447 [...]
        0.830988196507629 0.857225748552666 0.827138489800772
  </spatial:spatialPoints>
  <spatial:listOfParametricObjects>
    <spatial:parametricObject spatial:id="nucObj" spatial:polygonType="triangle"
                              spatial:domainType="nuc" spatial:pointIndexLength="24414"
                              spatial:compression="uncompressed">
        69 225 253 247 217 24 181 451 452 [...] 732 2417 3925
    </spatial:parametricObject>
    <spatial:parametricObject spatial:id="cellObj" spatial:polygonType="triangle"
                              spatial:domainType="cell" spatial:pointIndexLength="46242"
                              spatial:compression="uncompressed">
        423 8 418 2240 439 180 187 97 299 [...] 8197 1262 3043
    </spatial:parametricObject>
  </spatial:listOfParametricObjects>
</spatial:parametricGeometry>
```

Here, an array of 179901 values is defined in the **SpatialPoints** object. Because this is a model with 3D geometry (described elsewhere in the model), the points are grouped in threes, with the first value representing the X value for the first point, the second value the Y of the first point, and the third the Z value of the first point. The next value (elided) would be the X value of the second point, etc., until the final three values encode the final point, for a total of 59967 points (179901/3). The first point (-0.472894296, -0.4420042305, -0.457890447) is referenced by its index of "`0`", the second by its index of "`1`", etc., through (0.830988196507629, 0.857225748552666, 0.827138489800772) for the point with index 59966.

These points are then used as in two meshes: one for the nucObj, and one for the cellObj. Because both have the `polygonType` "`triangle`", the list of points are intrepreted in groups of three: (69, 225, 253) being the three vertices of the first triangle of "`nucObj`", (245, 217, 24) the vertices of the second, etc. Similarly, (423, 8, 418) is the first defined triangle for "`cellObj`", and (8197, 1262, 3043) the last.

Because so many values were included, a decision was made to omit the optional "`;`" characters, which would have principally served to increase the file size.

### 3.49 The MixedGeometry class

A **MixedGeometry** defines a **Geometry** constructed from a collection of various ***GeometryDefinition*** objects that together define the complete geometry for the **Model**. It has a child **ListOfGeometryDefinitions** object that behaves exactly the same as the **ListOfGeometryDefinitions** child of the **Geometry**, but instead of that collection of geometry definitions defining alternate geometries, or alternate ways to define one geometry, the collection of geometry definitions in a **MixedGeometry** together define a single space. For example, a **MixedGeometry** may contain a **ParametricGeometry** that defines the contours of a cell membrane, plus a **CSGeometry** that defines a sphere that models the nucleus of that cell. The definition of a **MixedGeometry** is shown in Figure 19. Its **OrdinalMapping** children define how those geometries overlap one another.

Note that every child ***GeometryDefinition*** of a **MixedGeometry** must have an `isActive` value of "`false`". 'Active' geometries are a concept that applies only to the **Model** and its direct children, not to component geometries of a **MixedGeometry**.

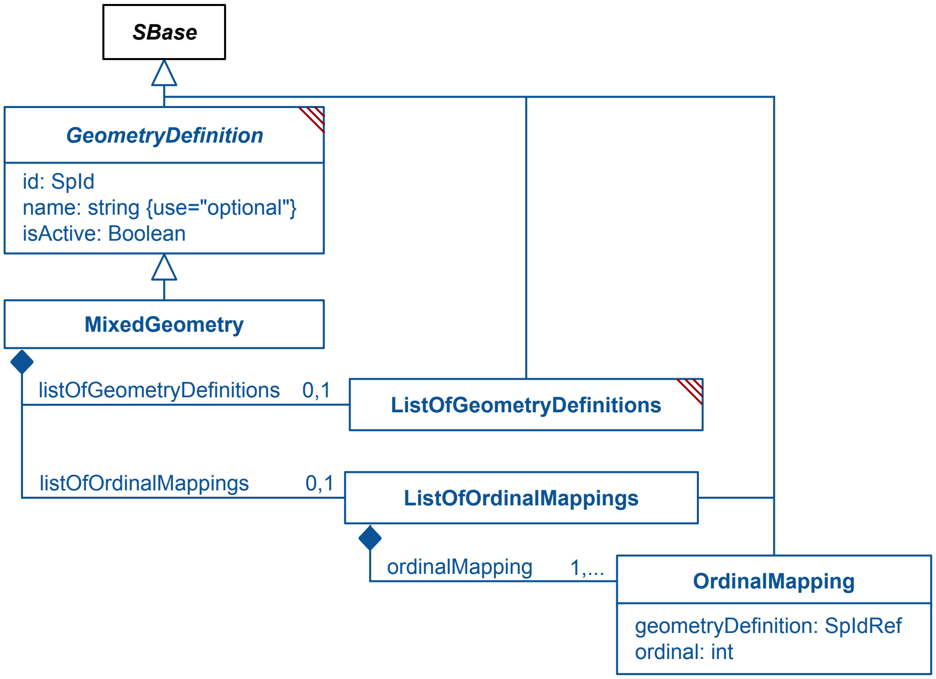

***Figure 19:*** *The definition of the* ***MixedGeometry****,* ***ListOfOrdinalMappings****, and* ***OrdinalMapping*** *classes from the Spatial package. The* ***ListOfGeometryDefinitions*** *class is defined in Section 3.17 on page 22*

### 3.50 The OrdinalMapping class

A **OrdinalMapping** defines an ordinal level for the various geometries that comprised this **MixedGeometry**. In this way, the overlap between them can be resolved cleanly. There must be exactly one **OrdinalMapping** for each child ***GeometryDefinition*** of a **MixedGeometry**.

#### *3.50.1 The* `geometryDefinition` *attribute*

The tokengeometryDefinition attribute is of type `SpIdRef`, and is required. It must reference a direct child ***GeometryDefinition*** of this **MixedGeometry**. The `ordinal` value is then taken to refer to that geometry.

#### *3.50.2 The* `ordinal` *attribute*

The `ordinal` attribute is of type `int`, and is required. It is used to represent the order of the corresponding ***GeometryDefinition*** within this **MixedGeometry**. The `ordinal` is useful while reconstructing the geometry in the specific software tool - it represents the order in which each ***GeometryDefinition*** have to be evaluated.

Rather than struggle with the task of preventing overlapping regions of space from each different ***GeometryDefinition***, they are to be considered to be evaluated in the reverse order of their ordinals. In this way, any ***GeometryDefinition*** that has already been processed will cover those with a smaller ordinal, thus resolving any ambiguities and removing the constraint that each ***GeometryDefinition*** be disjoint and cover the entire geometric domain. The ***GeometryDefinition*** with `ordinal` 0 can be the "background" layer (typically the extracellular space).

No two ***GeometryDefinition*** elements should have the same `ordinal` value, even if they should not overlap, because some tools may not calculate the geometries to the same level of precision as other tools, and may end up with overlap due to rounding errors, and will still need to resolve the ambiguity for their own purposes. If a software tool discovers two overlapping ***GeometryDefinition*** elements with the same `ordinal` value, it may resolve the situation however it sees fit.

Note that these ordinals only apply at the level of the immediate parent **MixedGeometry**. Any `ordinal` attribute from any **AnalyticVolume** or **CSGObject** applies only at the level of those volumes or objects, and serve to distinguish within-***GeometryDefinition*** layout order, not between-***GeometryDefinition*** layout order, defined here. Likewise, if a ***GeometryDefinition*** child of a **MixedGeometry** it itself a **MixedGeometry**, those ordinals also only apply at the level of that **MixedGeometry**, and not at the level of the parent **MixedGeometry**.

## 4 Examples

Several models have been created to demonstrate the basic capabilities of the 'spatial' package. Most of the smaller examples in this specification are snippets from these models.

- https://github.com/sbmlteam/sbml-specifications/tree/release/sbml-level-3/version-1/spatial/specification/examples/analytic_3d.xml: An example that uses the **AnalyticGeometry** construct to define spatial domains by the application of mathematical formulas.
- https://github.com/sbmlteam/sbml-specifications/tree/release/sbml-level-3/version-1/spatial/specification/examples/csgOnly.xml: An example that uses the **CSGeometry** construct to define spatial domains through building elements based on primitive shapes.
- https://github.com/sbmlteam/sbml-specifications/tree/release/sbml-level-3/version-1/spatial/specification/examples/parametric_1dom.xml: An example that uses the **ParametricGeometry** construct to define spatial domains through building a mesh that defines a surface.
- https://github.com/sbmlteam/sbml-specifications/tree/release/sbml-level-3/version-1/spatial/specification/examples/parametric_2dom.xml: A slightly more complicated **ParametricGeometry** example that defines three nested three-dimensional domains, with two-dimensional domains at the boundaries.
- https://github.com/sbmlteam/sbml-specifications/tree/release/sbml-level-3/version-1/spatial/specification/examples/sampledfield_3d.xml: An example that uses the **SampledFieldGeometry** construct to define spatial domains through assigning different values in a field (such as those produced in an image) to different domains.
- https://github.com/sbmlteam/sbml-specifications/tree/release/sbml-level-3/version-1/spatial/specification/examples/sampledfield_asnt.xml: The same **SampledFieldGeometry** example extended to use the **SampledField** values in an initial assignment for a species.

## 5 Interaction with other packages

The SBML Spatial package was designed to be orthogonal to other SBML packages, and as such should interact freely with all of them. However, there are no current implementations that take use of these interactions, so the following descriptions should all be taken as guidelines for future development, and do not represent established protocols at this time.

### 5.1 SBML Level 3 Version 2

This package may be used with either SBML Level 3 Version 1 Core, or SBML Level 3 Version 2 Core, with no essential change in functionality. SBML L3v2 adds an 'id' and 'name' to all constructs, so a few more Spatial elements that don't currently have an id would gain one. Those ids would fall in the SId namespace and not interact with the rest of the Spatial package.

Even though Level 3 Version 2 elements may reference package elements in the SId namespace with mathematical meaning, it is impossible to do this with Spatial elements, as the ids of all elements with mathematical meaning are in the SpId namespace instead. Both versions of SBML Level 3 should handle this the same way.

### 5.2 The `comp` package

The Hierarchical Model Composition package allows models to be composed hierarchically, with submodels that become part of the containing model, and connections that define how to integrate the submodels into the containing model. Spatial models may be composed in this way just like other SBML constructs, with the caveat that because Spatial ids are in the SpId namespace, and because comp does not come with an element of type SpIdRef, metaids must be used instead.

Also, since there may only be a single **Geometry** object child of the **Model**, the **Geometry** of any child submodel must be integrated into the parent model's **Geometry**: the `id`, `name`, and `coordinateSystem` of the parent remains unchanged, and the children of the **Geometry** should be merged into the parent **Geometry** in much the same way that the children of the child **Model** are merged into the parent **Model**.

### 5.3 The `distrib` package

The Distributions package allows modelers to include new MathML `csymbol` constructs that define draws from distributions, and the ability to define the uncertainty in any element with mathematical meaning. Both could be employed as-is by users of the Spatial package: the `csymbol` constructs can be used in spatial MathML, and in core MathML to affect spatial elements through the **SpatialSymbolReference** link. Any Spatial element with mathematical meaning could additionally be given an **Uncertainty** child defining its uncertainty.

### 5.4 The `multi` package

The Multistate and Multicomponent package allows modelers to define template species and reactions that are only realized upon actually simulating the model. In principle, the extended **Species** and **Reaction** objects defined in that package should be able to be placed in a spatial context.

The work involved in interpreting and simulating such a model would be substantial, however, and more research needs to be done to identify best practices in using the two packages together.

### 5.5 The `qual` package

The Qualitative Models package allows modelers to define interaction networks that are defined by element state instead of element levels. In principle, such state transition modeling could be modeled in a spatially-distinguished context, but no effort has been made to do so at this time. Mechanically, new extensions would have to be created to allow the **QualitativeSpecies** and **Transition** elements from the `qual` package to be spatially defined before any such model definitions could be created.

### 5.6 The `layout` and `render` packages

The Layout Package and the Render Package allow the visualization of SBML models. In principle, both could be used as-is to display the underlying reaction networks being used in a spatial model. However, there are no special constructs available to visualize the geometry defined in the spatial package: simulators typically display this geometry to the user with species levels superimposed, but nothing in `layout` or `render` would help the simulator displaying this information. Thus, at present, the **Geometry** itself is used for visualization of spatial simulations, while `layout` and `render` are used separately to visualize the reaction network.

## A Validation of SBML documents

### A.1 Validation and consistency rules

This section summarizes all the conditions that must (or in some cases, at least *should*) be true of an SBML Level 3 Version 1 model that uses the Spatial Processes. We use the same conventions as are used in the SBML Level 3 Version 1 Core specification document. In particular, there are different degrees of rule strictness. Formally, the differences are expressed in the statement of a rule: either a rule states that a condition *must* be true, or a rule states that it *should* be true. Rules of the former kind are strict SBML validation rules—a model encoded in SBML must conform to all of them in order to be considered valid. Rules of the latter kind are consistency rules. To help highlight these differences, we use the following three symbols next to the rule numbers:

☑ A checked box indicates a *requirement* for SBML conformance. If a model does not follow this rule, it does not conform to the Spatial Processes specification. (Mnemonic intention behind the choice of symbol: "This must be checked.")

▲ A triangle indicates a *recommendation* for model consistency. If a model does not follow this rule, it is not considered strictly invalid as far as the Spatial Processes specification is concerned; however, it indicates that the model contains a physical or conceptual inconsistency. (Mnemonic intention behind the choice of symbol: "This is a cause for warning.")

★ A star indicates a strong recommendation for good modeling practice. This rule is not strictly a matter of SBML encoding, but the recommendation comes from logical reasoning. As in the previous case, if a model does not follow this rule, it is not strictly considered an invalid SBML encoding. (Mnemonic intention behind the choice of symbol: "You're a star if you heed this.")

The validation rules listed in the following subsections are all stated or implied in the rest of this specification document. They are enumerated here for convenience. Unless explicitly stated, all validation rules concern objects and attributes specifically defined in the Spatial Processes package.

☞ For convenience and brevity, we use the shorthand "`spatial:x`" to stand for an attribute or element name `x` in the namespace for the Spatial Processes package, using the namespace prefix `spatial`. In reality, the prefix string may be different from the literal "`spatial`" used here (and indeed, it can be any valid XML namespace prefix that the modeler or software chooses). We use "`spatial:x`" because it is shorter than to write a full explanation everywhere we refer to an attribute or element in the Spatial Processes namespace.

Attributes from this package are listed in these rules as having the "`spatial:`" prefix, but as is convention for SBML packages, this prefix is optional.

#### *General rules about this package*

**spatial-10101** ☑ To conform to the Spatial Processes specification for SBML Level 3 Version 1, an SBML document must declare "`http://www.sbml.org/sbml/level3/version1/spatial/version1`" as the XMLNamespace to use for elements of this package. (Reference: SBML Level 3 Specification for Spatial Processes, Version 1 Section 3.2 on page 8.)

**spatial-10102** ☑ Wherever they appear in an SBML document, elements and attributes from the Spatial Processes must use the "`http://www.sbml.org/sbml/level3/version1/spatial/version1`" namespace, declaring so either explicitly or implicitly. (Reference: SBML Level 3 Specification for Spatial Processes, Version 1 Section 3.2 on page 8.)

#### *General rules about identifiers*

**spatial-10301** ☑ The value of the attribute `id` on every instance of the following classes of objects must be unique across the set of all `id` attribute values of all such objects in the model: **AdjacentDo-**

**mains**, **AdvectionCoefficient**, **AnalyticGeometry**, **AnalyticVolume**, **Boundary**, **BoundaryCondition**, **CSGHomogeneousTransformation**, ***CSGNode***, **CSGObject**, **CSGPrimitive**, **CSGRotation**, **CSGScale**, **CSGSetOperator**, ***CSGTransformation***, **CSGTranslation**, **CSGeometry**, **CompartmentMapping**, **CoordinateComponent**, **DiffusionCoefficient**, **Domain**, **DomainType**, **Geometry**, ***GeometryDefinition***, **InteriorPoint**, **ListOfAdjacentDomains**, **ListOfAnalyticVolumes**, **ListOfCSGNodes**, **ListOfCSGObjects**, **ListOfCoordinateComponents**, **ListOfDomainTypes**, **ListOfDomains**, **ListOfGeometryDefinitions**, **ListOfInteriorPoints**, **ListOfOrdinalMappings**, **ListOfParametricObjects**, **ListOfSampledFields**, **ListOfSampledVolumes**, **MixedGeometry**, **OrdinalMapping**, **ParametricGeometry**, **ParametricObject**, **SampledField**, **SampledFieldGeometry**, **SampledVolume**, **SpatialPoints**, **SpatialSymbolReference**, and **TransformationComponent**. All of these objects are defined to be in their own spatial "`SpId`" namespace of the **Model**. None of them are part of any other namespace (including the SId and UnitSId namespaces in core SBML), and need not be unique among the set of `id` values from core elements nor elements from other packages. (Reference: SBML Level 3 Specification for Spatial Processes, Version 1 Section 3.3.1 on page 9.)

**spatial-10302** ☑ The value of a `spatial:id` must conform to the syntax of the **SBML** data type `SId` (Reference: SBML Level 3 Version 1 Core, Section 3.1.7.)

#### *Rules for the extended SBML class*

**spatial-20101** ☑ In all SBML documents using the Spatial Processes, the **SBML** object must have the `spatial:required` attribute. (Reference: SBML Level 3 Version 1 Core, Section 4.1.2.)

**spatial-20102** ☑ The value of attribute `spatial:required` on the **SBML** object must be of data type `boolean`. (Reference: SBML Level 3 Version 1 Core, Section 4.1.2.)

**spatial-20103** ☑ The value of attribute `spatial:required` on the **SBML** object must be set to "`true`". (Reference: SBML Level 3 Specification for Spatial Processes, Version 1 Section 3.2 on page 8.)

#### *Rules for extended Model object*

**spatial-20201** ☑ A **Model** object may contain one and only one instance of the **Geometry** element. No other elements from the SBML Level 3 Spatial Processes namespaces are permitted on a **Model** object. (Reference: SBML Level 3 Specification for Spatial Processes, Version 1, Section 3.4 on page 11.)

#### *Rules for extended Compartment object*

**spatial-20301** ☑ A **Compartment** object may contain one and only one instance of the **CompartmentMapping** element. No other elements from the SBML Level 3 Spatial Processes namespaces are permitted on a **Compartment** object. (Reference: SBML Level 3 Specification for Spatial Processes, Version 1, Section 3.5 on page 11.)

#### *Rules for extended Species object*

**spatial-20401** ☑ A **Species** object may have the optional attribute `spatial:isSpatial`. No other attributes from the SBML Level 3 Spatial Processes namespaces are permitted on a **Species** object. (Reference: SBML Level 3 Specification for Spatial Processes, Version 1, Section 3.7 on page 13.)

**spatial-20402** ☑ The attribute `spatial:isSpatial` on a **Species** must have a value of data type `boolean`. (Reference: SBML Level 3 Specification for Spatial Processes, Version 1, Section 3.7 on page 13.)

**spatial-20450** ☑ The **Compartment** of any **Species** that has a value of "`true`" for the `spatial:isSpatial` attribute must have a child **CompartmentMapping**. (Reference: SBML Level 3 Specification for Spatial Processes, Version 1, Section 3.7 on page 13.)

#### Rules for extended Parameter object

**spatial-20501** ☑ A **Parameter** object may contain up to one child element from the SBML Level 3 Spatial Processes namespace: a **SpatialSymbolReference**, an **AdvectionCoefficient**, a **BoundaryCondition**, or a **DiffusionCoefficient**. (Reference: SBML Level 3 Specification for Spatial Processes, Version 1, Section 3.8 on page 14.)

#### Rules for extended Reaction object

**spatial-20601** ☑ A **Reaction** object must have the required attribute `spatial:isLocal`. No other attributes from the SBML Level 3 Spatial Processes namespaces are permitted on a **Reaction** object. (Reference: SBML Level 3 Specification for Spatial Processes, Version 1, Section 3.14 on page 18.)

**spatial-20602** ☑ The attribute `spatial:isLocal` on a **Reaction** must have a value of data type `boolean`. (Reference: SBML Level 3 Specification for Spatial Processes, Version 1, Section 3.14 on page 18.)

**spatial-20650** ☑ If the attribute `spatial:isLocal` of a **Reaction** is "`true`", the **Reaction** must also define a value for the `compartment` attribute. (Reference: SBML Level 3 Specification for Spatial Processes, Version 1, Section 3.14 on page 18.)

**spatial-20651** ▲ The units of a **KineticLaw** of a **Reaction** with a `spatial:isLocal` attribute value of "`true`" should be $concentration/time$ instead of $substance/time$. (Reference: SBML Level 3 Specification for Spatial Processes, Version 1, Section 3.14 on page 18.)

#### Rules for DomainType object

**spatial-20701** ☑ A **DomainType** object may have the optional SBML Level 3 Core attributes `metaid` and `sboTerm`. No other attributes from the SBML Level 3 Core namespaces are permitted on a **DomainType**. (Reference: SBML Level 3 Version 1 Core, Section 3.2.)

**spatial-20702** ☑ A **DomainType** object may have the optional SBML Level 3 Core subobjects for notes and annotations. No other elements from the SBML Level 3 Core namespaces are permitted on a **DomainType**. (Reference: SBML Level 3 Version 1 Core, Section 3.2.)

**spatial-20703** ☑ A **DomainType** object must have the required attributes `spatial:id` and `spatialDimensions`, and may have the optional attribute `spatial:name`. No other attributes from the SBML Level 3 Spatial Processes namespaces are permitted on a **DomainType** object. (Reference: SBML Level 3 Specification for Spatial Processes, Version 1, Section 3.19 on page 25.)

**spatial-20704** ☑ The attribute `spatial:spatialDimensions` on a **DomainType** must have a value of data type `integer`. (Reference: SBML Level 3 Specification for Spatial Processes, Version 1, Section 3.19 on page 25.)

**spatial-20705** ☑ The attribute `spatial:name` on a **DomainType** must have a value of data type `string`. (Reference: SBML Level 3 Specification for Spatial Processes, Version 1, Section 3.19 on page 25.)

**spatial-20750** ☑ If the **Geometry** of the **Model** has exactly three **CoordinateComponent** children, the attribute `spatial:spatialDimensions` of a **DomainType** may only have a value of "`2`" or "`3`". (Reference: SBML Level 3 Specification for Spatial Processes, Version 1, Section 3.19 on page 25.)

**spatial-20751** ☑ If the **Geometry** of the **Model** has exactly two **CoordinateComponent** children, the attribute `spatial:spatialDimensions` of a **DomainType** may only have a value of "`1`" or "`2`". (Reference: SBML Level 3 Specification for Spatial Processes, Version 1, Section 3.19 on page 25.)

**spatial-20752** ☑ If the **Geometry** of the **Model** has exactly one **CoordinateComponent** child, the attribute `spatial:spatialDimensions` of a **DomainType** may only have a value of "`0`" or "`1`". (Reference: SBML Level 3 Specification for Spatial Processes, Version 1, Section 3.19 on page 25.)

**spatial-20753** ☑ If a **Parameter** has a child **SpatialSymbolReference** that points to a **DomainType**, that **Parameter** may not be the target of an **InitialAssignment**, **EventAssignment**, **RateRule**, or **AssignmentRule**, may not be determined by an **AlgebraicRule**, and may not define the `value` attribute. (Reference: SBML Level 3 Specification for Spatial Processes, Version 1, Section 3.19 on page 25.)

#### *Rules for Domain object*

**spatial-20801** ☑ A **Domain** object may have the optional SBML Level 3 Core attributes `metaid` and `sboTerm`. No other attributes from the SBML Level 3 Core namespaces are permitted on a **Domain**. (Reference: SBML Level 3 Version 1 Core, Section 3.2.)

**spatial-20802** ☑ A **Domain** object may have the optional SBML Level 3 Core subobjects for notes and annotations. No other elements from the SBML Level 3 Core namespaces are permitted on a **Domain**. (Reference: SBML Level 3 Version 1 Core, Section 3.2.)

**spatial-20803** ☑ A **Domain** object must have the required attributes `spatial:id` and `spatial:domainType`, and may have the optional attribute `spatial:name`. No other attributes from the SBML Level 3 Spatial Processes namespaces are permitted on a **Domain** object. (Reference: SBML Level 3 Specification for Spatial Processes, Version 1, Section 3.20 on page 26.)

**spatial-20804** ☑ A **Domain** object may contain one and only one instance of the **ListOfInteriorPoints** element. No other elements from the SBML Level 3 Spatial Processes namespaces are permitted on a **Domain** object. (Reference: SBML Level 3 Specification for Spatial Processes, Version 1, Section 3.20 on page 26.)

**spatial-20805** ☑ The value of the attribute `spatial:domainType` of a **Domain** object must be the identifier of an existing **DomainType** object defined in the enclosing **Model** object. (Reference: SBML Level 3 Specification for Spatial Processes, Version 1, Section 3.20 on page 26.)

**spatial-20806** ☑ The attribute `spatial:name` on a **Domain** must have a value of data type `string`. (Reference: SBML Level 3 Specification for Spatial Processes, Version 1, Section 3.20 on page 26.)

**spatial-20807** ☑ Apart from the general notes and annotations subobjects permitted on all SBML objects, a **ListOfInteriorPoints** container object may only contain **InteriorPoint** objects. (Reference: SBML Level 3 Specification for Spatial Processes, Version 1, Section 3.20 on page 26.)

**spatial-20808** ☑ A **ListOfInteriorPoints** object may have the optional SBML Level 3 Core attributes `metaid` and `sboTerm`. No other attributes from the SBML Level 3 Core namespaces are permitted on a **ListOfInteriorPoints** object. (Reference: SBML Level 3 Specification for Spatial Processes, Version 1, Section 3.20 on page 26.)

**spatial-20850** ☑ If a **Parameter** has a child **SpatialSymbolReference** that points to a **Domain**, that **Parameter** may not be the target of an **InitialAssignment**, **EventAssignment**, **RateRule**, or **AssignmentRule**, may not be determined by an **AlgebraicRule**, and may not define the `value` attribute. (Reference: SBML Level 3 Specification for Spatial Processes, Version 1, Section 3.20 on page 26.)

#### *Rules for InteriorPoint object*

**spatial-20901** ☑ An **InteriorPoint** object may have the optional SBML Level 3 Core attributes `metaid` and `sboTerm`. No other attributes from the SBML Level 3 Core namespaces are permitted on an **InteriorPoint**. (Reference: SBML Level 3 Version 1 Core, Section 3.2.)

**spatial-20902** ☑ An **InteriorPoint** object may have the optional SBML Level 3 Core subobjects for notes and annotations. No other elements from the SBML Level 3 Core namespaces are permitted on an **InteriorPoint**. (Reference: SBML Level 3 Version 1 Core, Section 3.2.)

**spatial-20903** ☑ An **InteriorPoint** object must have the required attribute `spatial:coord1`, and may have the optional attributes `spatial:coord2` and `spatial:coord3`. No other attributes from the SBML Level 3 Spatial Processes namespaces are permitted on an **InteriorPoint** object. (Reference: SBML Level 3 Specification for Spatial Processes, Version 1, Section 3.21 on page 27.)

**spatial-20904** ☑ The attribute `spatial:coord1` on an **InteriorPoint** must have a value of data type `double`. (Reference: SBML Level 3 Specification for Spatial Processes, Version 1, Section 3.21 on page 27.)

**spatial-20905** ☑ The attribute `spatial:coord2` on an **InteriorPoint** must have a value of data type `double`. (Reference: SBML Level 3 Specification for Spatial Processes, Version 1, Section 3.21 on page 27.)

**spatial-20906** ☑ The attribute `spatial:coord3` on an **InteriorPoint** must have a value of data type `double`. (Reference: SBML Level 3 Specification for Spatial Processes, Version 1, Section 3.21 on page 27.)

**spatial-20950** ☑ If the **Geometry** of the **Model** has exactly one **CoordinateComponent** child, an **InteriorPoint** object must define the attribute `coord1`, and must not define the attributes `coord2` and `coord3`. (Reference: SBML Level 3 Specification for Spatial Processes, Version 1, Section 3.21 on page 27.)

**spatial-20951** ☑ If the **Geometry** of the **Model** has exactly two **CoordinateComponent** children, an **InteriorPoint** object must define the attributes `coord1` and `coord2`, and must not define the attribute `coord3`. (Reference: SBML Level 3 Specification for Spatial Processes, Version 1, Section 3.21 on page 27.)

**spatial-20952** ☑ If the **Geometry** of the **Model** has exactly three **CoordinateComponent** children, an **InteriorPoint** object must define the attributes `coord1`, `coord2`, and `coord3`. (Reference: SBML Level 3 Specification for Spatial Processes, Version 1, Section 3.21 on page 27.)

#### *Rules for Boundary object*

**spatial-21001** ☑ A **Boundary** object may have the optional SBML Level 3 Core attributes `metaid` and `sboTerm`. No other attributes from the SBML Level 3 Core namespaces are permitted on a **Boundary**. (Reference: SBML Level 3 Version 1 Core, Section 3.2.)

**spatial-21002** ☑ A **Boundary** object may have the optional SBML Level 3 Core subobjects for notes and annotations. No other elements from the SBML Level 3 Core namespaces are permitted on a **Boundary**. (Reference: SBML Level 3 Version 1 Core, Section 3.2.)

**spatial-21003** ☑ A **Boundary** object must have the required attributes `spatial:id` and `spatial:value`, and may have the optional attribute `spatial:name`. No other attributes from the SBML Level 3 Spatial Processes namespaces are permitted on a **Boundary** object. (Reference: SBML Level 3 Specification for Spatial Processes, Version 1, Section 3.18 on page 24.)

**spatial-21004** ☑ The attribute `spatial:value` on a **Boundary** must have a value of data type `double`. (Reference: SBML Level 3 Specification for Spatial Processes, Version 1, Section 3.18 on page 24.)

**spatial-21005** ☑ The attribute `spatial:name` on a **Boundary** must have a value of data type `string`. (Reference: SBML Level 3 Specification for Spatial Processes, Version 1, Section 3.18 on page 24.)

**spatial-21050** ☑ If set, the `value` attribute of a `<boundaryMin>` must be less than or equal to the `value` attribute of a `<boundaryMax>` of the same **CoordinateComponent**. (Reference: SBML Level 3 Specification for Spatial Processes, Version 1, Section 3.15 on page 18.)

**spatial-21051** ☑ If a **Parameter** has a child **SpatialSymbolReference** that points to a **Boundary**, the `constant` attribute of that **Parameter** must have a value of "`true`". (Reference: SBML Level 3 Specification for Spatial Processes, Version 1, Section 3.15 on page 18.)

**spatial-21052** ▲ If a **Parameter** has a child **SpatialSymbolReference** that points to a **Boundary**, the units of that **Parameter** should be equal to the units of the **Boundary**'s parent **CoordinateComponent**. (Reference: SBML Level 3 Specification for Spatial Processes, Version 1, Section 3.15 on page 18.)

#### *Rules for AdjacentDomains object*

**spatial-21101** ☑ An **AdjacentDomains** object may have the optional SBML Level 3 Core attributes `metaid` and `sboTerm`. No other attributes from the SBML Level 3 Core namespaces are permitted on an **AdjacentDomains**. (Reference: SBML Level 3 Version 1 Core, Section 3.2.)

**spatial-21102** ☑ An **AdjacentDomains** object may have the optional SBML Level 3 Core subobjects for notes and annotations. No other elements from the SBML Level 3 Core namespaces are permitted on an **AdjacentDomains**. (Reference: SBML Level 3 Version 1 Core, Section 3.2.)

**spatial-21103** ☑ An **AdjacentDomains** object must have the required attributes `spatial:id`, `spatial:domain1` and `spatial:domain2`, and may have the optional attribute `spatial:name`. No other attributes from the SBML Level 3 Spatial Processes namespaces are permitted on an **AdjacentDomains** object. (Reference: SBML Level 3 Specification for Spatial Processes, Version 1, Section 3.22 on page 27.)

**spatial-21104** ☑ The value of the attribute `spatial:domain1` of an **AdjacentDomains** object must be the identifier of an existing **Domain** object defined in the enclosing **Model** object. (Reference: SBML Level 3 Specification for Spatial Processes, Version 1, Section 3.22 on page 27.)

**spatial-21105** ☑ The value of the attribute `spatial:domain2` of an **AdjacentDomains** object must be the identifier of an existing **Domain** object defined in the enclosing **Model** object. (Reference: SBML Level 3 Specification for Spatial Processes, Version 1, Section 3.22 on page 27.)

**spatial-21106** ☑ The attribute `spatial:name` on an **AdjacentDomains** must have a value of data type `string`. (Reference: SBML Level 3 Specification for Spatial Processes, Version 1, Section 3.22 on page 27.)

**spatial-21150** ☑ The attributes `spatial:domain1` and `spatial:domain2` of an **AdjacentDomains** must reference two different domains that are spatially adjacent. (Reference: SBML Level 3 Specification for Spatial Processes, Version 1, Section 3.22 on page 27.)

#### *Rules for GeometryDefinition object*

**spatial-21201** ☑ A ***GeometryDefinition*** object may have the optional SBML Level 3 Core attributes `metaid` and `sboTerm`. No other attributes from the SBML Level 3 Core namespaces are permitted on a ***GeometryDefinition***. (Reference: SBML Level 3 Version 1 Core, Section 3.2.)

**spatial-21202** ☑ A ***GeometryDefinition*** object may have the optional SBML Level 3 Core subobjects for notes and annotations. No other elements from the SBML Level 3 Core namespaces are permitted on a ***GeometryDefinition***. (Reference: SBML Level 3 Version 1 Core, Section 3.2.)

**spatial-21203** ☑ A ***GeometryDefinition*** object must have the required attributes `spatial:id` and `spatial:isActive`, and may have the optional attribute `spatial:name`. No other attributes from the SBML Level 3 Spatial Processes namespaces are permitted on a ***GeometryDefinition*** object. (Reference: SBML Level 3 Specification for Spatial Processes, Version 1, Section 3.24 on page 28.)

**spatial-21204** ☑ The attribute `spatial:isActive` on a ***GeometryDefinition*** must have a value of data type `boolean`. (Reference: SBML Level 3 Specification for Spatial Processes, Version 1, Section 3.24 on page 28.)

**spatial-21205** ☑ The attribute `spatial:name` on a ***GeometryDefinition*** must have a value of data type `string`. (Reference: SBML Level 3 Specification for Spatial Processes, Version 1, Section 3.24 on page 28.)

**spatial-21250** ☑ At least one ***GeometryDefinition*** child of a **Geometry** must have a `spatial:isActive` attribute value of "`true`". (Reference: SBML Level 3 Specification for Spatial Processes, Version 1, Section 3.24 on page 28.)

#### *Rules for CompartmentMapping object*

**spatial-21301** ☑ A **CompartmentMapping** object may have the optional SBML Level 3 Core attributes `metaid` and `sboTerm`. No other attributes from the SBML Level 3 Core namespaces are permitted on a **CompartmentMapping**. (Reference: SBML Level 3 Version 1 Core, Section 3.2.)

**spatial-21302** ☑ A **CompartmentMapping** object may have the optional SBML Level 3 Core subobjects for notes and annotations. No other elements from the SBML Level 3 Core namespaces are permitted on a **CompartmentMapping**. (Reference: SBML Level 3 Version 1 Core, Section 3.2.)

**spatial-21303** ☑ A **CompartmentMapping** object must have the required attributes `spatial:id`, `spatial:domainType` and `spatial:unitSize`, and may have the optional attribute `spatial:name`. No other attributes from the SBML Level 3 Spatial Processes namespaces are permitted on a **CompartmentMapping** object. (Reference: SBML Level 3 Specification for Spatial Processes, Version 1, Section 3.6 on page 12.)

**spatial-21304** ☑ The value of the attribute `spatial:domainType` of a **CompartmentMapping** object must be the identifier of an existing **DomainType** object defined in the enclosing **Model** object. (Reference: SBML Level 3 Specification for Spatial Processes, Version 1, Section 3.6 on page 12.)

**spatial-21305** ☑ The attribute `spatial:unitSize` on a **CompartmentMapping** must have a value of data type `double`. (Reference: SBML Level 3 Specification for Spatial Processes, Version 1, Section 3.6 on page 12.)

**spatial-21306** ☑ The attribute `spatial:name` on a **CompartmentMapping** must have a value of data type `string`. (Reference: SBML Level 3 Specification for Spatial Processes, Version 1, Section 3.6 on page 12.)

**spatial-21350** ▲ The value of the attribute `spatial:unitSize` on a **CompartmentMapping** should have a value between 0 and 1, inclusive, when the dimensions of the referenced compartments are the same. (Reference: SBML Level 3 Specification for Spatial Processes, Version 1, Section 3.6 on page 12.)

**spatial-21351** ▲ The values of the `spatial:unitSize` attributes of every **CompartmentMapping** with the same `spatial:domainType` should sum to 1, when the dimensions of the referenced compartments are the same. (Reference: SBML Level 3 Specification for Spatial Processes, Version 1, Section 3.6 on page 12.)

**spatial-21352** ▲ If a **Parameter** has a child **SpatialSymbolReference** that points to a **CompartmentMapping**, the units of that **Parameter** should be equivalent to the units of the parent **Compartment** of the **CompartmentMapping**, divided by the units of the **DomainType** referenced by the **CompartmentMapping**. (Reference: SBML Level 3 Specification for Spatial Processes, Version 1, Section 3.6 on page 12.)

#### *Rules for CoordinateComponent object*

**spatial-21401** ☑ A **CoordinateComponent** object may have the optional SBML Level 3 Core attributes `metaid` and `sboTerm`. No other attributes from the SBML Level 3 Core namespaces are permitted on a **CoordinateComponent**. (Reference: SBML Level 3 Version 1 Core, Section 3.2.)

**spatial-21402** ☑ A **CoordinateComponent** object may have the optional SBML Level 3 Core subobjects for notes and annotations. No other elements from the SBML Level 3 Core namespaces are permitted on a **CoordinateComponent**. (Reference: SBML Level 3 Version 1 Core, Section 3.2.)

**spatial-21403** ☑ A **CoordinateComponent** object must have the required attributes `spatial:id` and `spatial:type`, and may have the optional attributes `spatial:name` and `spatial:unit`. No other attributes from the SBML Level 3 Spatial Processes namespaces are permitted on a **CoordinateComponent** object. (Reference: SBML Level 3 Specification for Spatial Processes, Version 1, Section 3.15 on page 18.)

**spatial-21404** ☑ A **CoordinateComponent** object must contain one and only one instance of each of the two **Boundary** elements "`boundaryMin`" and "`boundaryMax`". No other elements from the SBML Level 3 Spatial Processes namespaces are permitted on a **CoordinateComponent** object. (Reference: SBML Level 3 Specification for Spatial Processes, Version 1, Section 3.15 on page 18.)

**spatial-21405** ☑ The value of the attribute `spatial:type` of a **CoordinateComponent** object must conform to the syntax of SBML data type `CoordinateKind` and may only take on the allowed values of `CoordinateKind` defined in SBML; that is, the value must be one of the following: "`cartesianX`", "`cartesianY`" or "`cartesianZ`". (Reference: SBML Level 3 Specification for Spatial Processes, Version 1, Section 3.15 on page 18.)

**spatial-21406** ☑ The attribute `spatial:name` on a **CoordinateComponent** must have a value of data type `string`. (Reference: SBML Level 3 Specification for Spatial Processes, Version 1, Section 3.15 on page 18.)

**spatial-21407** ☑ The value of the attribute `spatial:unit` on a **CoordinateComponent** must have a taken from the following: the identifier of a **UnitDefinition** object in the enclosing **Model**, or one of the base units in SBML. (Reference: SBML Level 3 Specification for Spatial Processes, Version 1, Section 3.15 on page 18.)

#### *Rules for SampledFieldGeometry object*

**spatial-21501** ☑ A **SampledFieldGeometry** object may have the optional SBML Level 3 Core attributes `metaid` and `sboTerm`. No other attributes from the SBML Level 3 Core namespaces are permitted on a **SampledFieldGeometry**. (Reference: SBML Level 3 Version 1 Core, Section 3.2.)

**spatial-21502** ☑ A **SampledFieldGeometry** object may have the optional SBML Level 3 Core subobjects for notes and annotations. No other elements from the SBML Level 3 Core namespaces are permitted on a **SampledFieldGeometry**. (Reference: SBML Level 3 Version 1 Core, Section 3.2.)

**spatial-21503** ☑ A **SampledFieldGeometry** object must have the required attribute `spatial:sampledField`. No other attributes from the SBML Level 3 Spatial Processes namespaces are permitted on a **SampledFieldGeometry** object. (Reference: SBML Level 3 Specification for Spatial Processes, Version 1, Section 3.29 on page 31.)

**spatial-21504** ☑ A **SampledFieldGeometry** object may contain one and only one instance of the **ListOfSampledVolumes** element. No other elements from the SBML Level 3 Spatial Processes namespaces are permitted on a **SampledFieldGeometry** object. (Reference: SBML Level 3 Specification for Spatial Processes, Version 1, Section 3.29 on page 31.)

**spatial-21505** ☑ The value of the attribute `spatial:sampledField` of a **SampledFieldGeometry** object must be the identifier of an existing **SampledField** object defined in the enclosing **Model** object. (Reference: SBML Level 3 Specification for Spatial Processes, Version 1, Section 3.29 on page 31.)

**spatial-21506** ☑ Apart from the general notes and annotations subobjects permitted on all SBML objects, a **ListOfSampledVolumes** container object may only contain **SampledVolume** objects. (Reference: SBML Level 3 Specification for Spatial Processes, Version 1, Section 3.29 on page 31.)

**spatial-21507** ☑ A **ListOfSampledVolumes** object may have the optional SBML Level 3 Core attributes `metaid` and `sboTerm`. No other attributes from the SBML Level 3 Core namespaces are permitted on a **ListOfSampledVolumes** object. (Reference: SBML Level 3 Specification for Spatial Processes, Version 1, Section 3.29 on page 31.)

#### *Rules for SampledField object*

**spatial-21601** ☑ A **SampledField** object may have the optional SBML Level 3 Core attributes `metaid` and `sboTerm`. No other attributes from the SBML Level 3 Core namespaces are permitted on a **SampledField**. (Reference: SBML Level 3 Version 1 Core, Section 3.2.)

**spatial-21602** ☑ A **SampledField** object may have the optional SBML Level 3 Core subobjects for notes and annotations. No other elements from the SBML Level 3 Core namespaces are permitted on a **SampledField**. (Reference: SBML Level 3 Version 1 Core, Section 3.2.)

**spatial-21603** ☑ A **SampledField** object must have the required attributes `spatial:id`, `spatial:dataType`, `spatial:numSamples1`, `spatial:interpolationType`, `spatial:compression`, and `spatial-:samplesLength`, and may have the optional attributes `spatial:name`, `spatial:numSamples2` and `spatial:numSamples3`. No other attributes from the SBML Level 3 Spatial Processes namespaces are permitted on a **SampledField** object. (Reference: SBML Level 3 Specification for Spatial Processes, Version 1, Section 3.16 on page 20.)

**spatial-21604** ☑ The value of the attribute `spatial:dataType` of a **SampledField** object must conform to the syntax of SBML data type `DataKind` and may only take on the allowed values of `DataKind` defined in SBML; that is, the value must be one of the following: "`double`", "`float`", "`int`", "`uint`", "`uint8`", "`uint16`" or "`uint32`". (Reference: SBML Level 3 Specification for Spatial Processes, Version 1, Section 3.16 on page 20.)

**spatial-21605** ☑ The attribute `spatial:numSamples1` on a **SampledField** must have a value of data type `integer`. (Reference: SBML Level 3 Specification for Spatial Processes, Version 1, Section 3.16 on page 20.)

**spatial-21606** ☑ The value of the attribute `spatial:interpolationType` of a **SampledField** object must conform to the syntax of SBML data type `InterpolationKind` and may only take on the allowed values of `InterpolationKind` defined in SBML; that is, the value must be one of the following: "`nearestNeighbor`" or "`linear`". (Reference: SBML Level 3 Specification for Spatial Processes, Version 1, Section 3.16 on page 20.)

**spatial-21607** ☑ The value of the attribute `spatial:compression` of a **SampledField** object must conform to the syntax of SBML data type `CompressionKind` and may only take on the allowed values of `CompressionKind` defined in SBML; that is, the value must be one of the following: "`uncompressed`" or "`deflated`". (Reference: SBML Level 3 Specification for Spatial Processes, Version 1, Section 3.16 on page 20.)

**spatial-21608** ☑ The value of the text child of a **SampledField** object must be an array of numeric values. (Reference: SBML Level 3 Specification for Spatial Processes, Version 1, Section 3.16 on page 20.)

**spatial-21609** ☑ The attribute `spatial:samplesLength` on a **SampledField** must have a value of data type `integer`. (Reference: SBML Level 3 Specification for Spatial Processes, Version 1, Section 3.16 on page 20.)

**spatial-21610** ☑ The attribute `spatial:name` on a **SampledField** must have a value of data type `string`. (Reference: SBML Level 3 Specification for Spatial Processes, Version 1, Section 3.16 on page 20.)

**spatial-21611** ☑ The attribute `spatial:numSamples2` on a **SampledField** must have a value of data type `integer`. (Reference: SBML Level 3 Specification for Spatial Processes, Version 1, Section 3.16 on page 20.)

**spatial-21612** ☑ The attribute `spatial:numSamples3` on a **SampledField** must have a value of data type `integer`. (Reference: SBML Level 3 Specification for Spatial Processes, Version 1, Section 3.16 on page 20.)

**spatial-21650** ☑ If the **Geometry** of the **Model** has exactly one **CoordinateComponent** child, a **SampledField** object must define the attribute `spatial:numSamples1`, and must not define the attributes `spatial:numSamples2` nor `spatial:numSamples3`. (Reference: SBML Level 3 Specification for Spatial Processes, Version 1, Section 3.16 on page 20.)

**spatial-21651** ☑ If the **Geometry** of the **Model** has exactly two **CoordinateComponent** children, a **SampledField** object must define the attributes `spatial:numSamples1` and `spatial:numSamples2`, and must not define the attribute `spatial:numSamples3`. (Reference: SBML Level 3 Specification for Spatial Processes, Version 1, Section 3.16 on page 20.)

**spatial-21652** ☑ If the **Geometry** of the **Model** has exactly three **CoordinateComponent** children, a **SampledField** object must define the attributes `spatial:numSamples1`, `spatial:numSamples2`, and `spatial:numSamples3`. (Reference: SBML Level 3 Specification for Spatial Processes, Version 1, Section 3.16 on page 20.)

**spatial-21653** ☑ If the `spatial:compression` attribute of a **SampledField** has the value "`uncompressed`", the `spatial:samplesLength` attribute of that **SampledField** must equal the number of entries in the **ArrayData** child of the **SampledField**. (Reference: SBML Level 3 Specification for Spatial Processes, Version 1, Section 3.16 on page 20.)

**spatial-21654** ☑ If the `spatial:compression` attribute of a **SampledField** has the value "`deflated`", the `spatial:samplesLength` attribute of that **SampledField** must equal the number of entries of the **ArrayData** child of the **SampledField**. (Reference: SBML Level 3 Specification for Spatial Processes, Version 1, Section 3.16 on page 20.)

**spatial-21655** ☑ If the `spatial:dataType` attribute of a **SampledField** has the value "`float`", none of the uncompressed entries in the **ArrayData** child of the **SampledField** may have a value outside of the range of an IEEE 754-1985 single-precision floating point value (approximately +/-3.4028235e38, and +/-1.17549e-38). (Reference: SBML Level 3 Specification for Spatial Processes, Version 1, Section 3.16 on page 20.)

**spatial-21656** ☑ If the `spatial:dataType` attribute of a **SampledField** has the value "`uint`", none of the uncompressed entries in the **ArrayData** child of the **SampledField** may be negative. (Reference: SBML Level 3 Specification for Spatial Processes, Version 1, Section 3.16 on page 20.)

**spatial-21657** ☑ If the `spatial:dataType` attribute of a **SampledField** has the value "`uint`" or "`int`", all of the uncompressed entries in the **ArrayData** child of the **SampledField** must be integers. (Reference: SBML Level 3 Specification for Spatial Processes, Version 1, Section 3.16 on page 20.)

**spatial-21658** ☑ The value of the children of a **SampledField** object must be an array of values of type `integer` if the `spatial:compression` attribute has the value "`deflated`". (Reference: SBML Level 3 Specification for Spatial Processes, Version 1, Section 3.16 on page 20.)

#### *Rules for SampledVolume object*

**spatial-21701** ☑ A **SampledVolume** object may have the optional SBML Level 3 Core attributes `metaid` and `sboTerm`. No other attributes from the SBML Level 3 Core namespaces are permitted on a **SampledVolume**. (Reference: SBML Level 3 Version 1 Core, Section 3.2.)

**spatial-21702** ☑ A **SampledVolume** object may have the optional SBML Level 3 Core subobjects for notes and annotations. No other elements from the SBML Level 3 Core namespaces are permitted on a **SampledVolume**. (Reference: SBML Level 3 Version 1 Core, Section 3.2.)

**spatial-21703** ☑ A **SampledVolume** object must have the required attributes `spatial:id` and `spatial:domainType`, and may have the optional attributes `spatial:name`, `spatial:sampledValue`, `spatial:minValue` and `spatial:maxValue`. No other attributes from the SBML Level 3 Spatial Processes namespaces are permitted on a **SampledVolume** object. (Reference: SBML Level 3 Specification for Spatial Processes, Version 1, Section 3.30 on page 31.)

**spatial-21704** ☑ The value of the attribute `spatial:domainType` of a **SampledVolume** object must be the identifier of an existing **DomainType** object defined in the enclosing **Model** object. (Reference: SBML Level 3 Specification for Spatial Processes, Version 1, Section 3.30 on page 31.)

**spatial-21705** ☑ The attribute `spatial:name` on a **SampledVolume** must have a value of data type `string`. (Reference: SBML Level 3 Specification for Spatial Processes, Version 1, Section 3.30 on page 31.)

**spatial-21706** ☑ The attribute `spatial:sampledValue` on a **SampledVolume** must have a value of data type `double`. (Reference: SBML Level 3 Specification for Spatial Processes, Version 1, Section 3.30 on page 31.)

**spatial-21707** ☑ The attribute `spatial:minValue` on a **SampledVolume** must have a value of data type `double`. (Reference: SBML Level 3 Specification for Spatial Processes, Version 1, Section 3.30 on page 31.)

**spatial-21708** ☑ The attribute `spatial:maxValue` on a **SampledVolume** must have a value of data type `double`. (Reference: SBML Level 3 Specification for Spatial Processes, Version 1, Section 3.30 on page 31.)

**spatial-21750** ☑ A **SampledVolume** either must define the attribute `spatial:sampledValue` or must define both the `spatial:minValue` and `spatial:maxValue` attributes, but may not define any other combination of those three attributes. (Reference: SBML Level 3 Specification for Spatial Processes, Version 1, Section 3.30 on page 31.)

**spatial-21751** ☑ The attribute `spatial:minValue` of a **SampledVolume** must have a value less than or equal to the value of the `spatial:maxValue` attribute of that **SampledVolume**. (Reference: SBML Level 3 Specification for Spatial Processes, Version 1, Section 3.30 on page 31.)

**spatial-21752** ☑ The `spatial:sampledValue` of one **SampledVolume** may not be the same as a `spatial:sampledValue` from a different **SampledVolume** from the same **SampledFieldGeometry**. (Reference: SBML Level 3 Specification for Spatial Processes, Version 1, Section 3.30 on page 31.)

**spatial-21753** ☑ The `spatial:sampledValue` from one **SampledVolume** may not be less than the value of the attribute `spatial:maxValue` and greater than or equal to the value of the attribute `spatial:minValue` of a different **SampledVolume** from the same **SampledFieldGeometry**. (Reference: SBML Level 3 Specification for Spatial Processes, Version 1, Section 3.30 on page 31.)

**spatial-21754** ☑ The spatial:minValue and spatial:maxValue attribute values from one **SampledVolume** may not define a range that overlaps the spatial:minValue and spatial:maxValue attribute values of a different **SampledVolume** from the same **SampledFieldGeometry**, with the exception that the spatial:maxValue of one **SampledVolume** may equal the spatial:minValue of another **SampledVolume** from the same **SampledFieldGeometry**. (Reference: SBML Level 3 Specification for Spatial Processes, Version 1, Section 3.30 on page 31.)

#### *Rules for AnalyticGeometry object*

**spatial-21801** ☑ An **AnalyticGeometry** object may have the optional SBML Level 3 Core attributes metaid and sboTerm. No other attributes from the SBML Level 3 Core namespaces are permitted on an **AnalyticGeometry**. (Reference: SBML Level 3 Version 1 Core, Section 3.2.)

**spatial-21802** ☑ An **AnalyticGeometry** object may have the optional SBML Level 3 Core subobjects for notes and annotations. No other elements from the SBML Level 3 Core namespaces are permitted on an **AnalyticGeometry**. (Reference: SBML Level 3 Version 1 Core, Section 3.2.)

**spatial-21803** ☑ An **AnalyticGeometry** object may contain one and only one instance of the **ListOfAnalyticVolumes** element. No other elements from the SBML Level 3 Spatial Processes namespaces are permitted on an **AnalyticGeometry** object. (Reference: SBML Level 3 Specification for Spatial Processes, Version 1, Section 3.25 on page 29.)

**spatial-21804** ☑ Apart from the general notes and annotations subobjects permitted on all SBML objects, a **ListOfAnalyticVolumes** container object may only contain **AnalyticVolume** objects. (Reference: SBML Level 3 Specification for Spatial Processes, Version 1, Section 3.25 on page 29.)

**spatial-21805** ☑ A **ListOfAnalyticVolumes** object may have the optional SBML Level 3 Core attributes metaid and sboTerm. No other attributes from the SBML Level 3 Core namespaces are permitted on a **ListOfAnalyticVolumes** object. (Reference: SBML Level 3 Specification for Spatial Processes, Version 1, Section 3.25 on page 29.)

#### *Rules for AnalyticVolume object*

**spatial-21901** ☑ An **AnalyticVolume** object may have the optional SBML Level 3 Core attributes metaid and sboTerm. No other attributes from the SBML Level 3 Core namespaces are permitted on an **AnalyticVolume**. (Reference: SBML Level 3 Version 1 Core, Section 3.2.)

**spatial-21902** ☑ An **AnalyticVolume** object may have the optional SBML Level 3 Core subobjects for notes and annotations. No other elements from the SBML Level 3 Core namespaces are permitted on an **AnalyticVolume**. (Reference: SBML Level 3 Version 1 Core, Section 3.2.)

**spatial-21903** ☑ An **AnalyticVolume** object must have the required attributes spatial:id, spatial:functionType and spatial:domainType, and may have the optional attributes spatial:name and spatial:ordinal. No other attributes from the SBML Level 3 Spatial Processes namespaces are permitted on an **AnalyticVolume** object. (Reference: SBML Level 3 Specification for Spatial Processes, Version 1, Section 3.26 on page 29.)

**spatial-21904** ☑ An **AnalyticVolume** object may contain one and only one instance of the ASTNode element. No other elements from the SBML Level 3 Spatial Processes namespaces are permitted on an **AnalyticVolume** object. (Reference: SBML Level 3 Specification for Spatial Processes, Version 1, Section 3.26 on page 29.)

**spatial-21905** ☑ The value of the attribute spatial:functionType of an **AnalyticVolume** object must conform to the syntax of SBML data type FunctionKind and may only take on the allowed value of FunctionKind defined in SBML; that is, the value must be "layered". (Reference: SBML Level 3 Specification for Spatial Processes, Version 1, Section 3.26 on page 29.)

**spatial-21906** ☑ The value of the attribute `spatial:domainType` of an **AnalyticVolume** object must be the identifier of an existing **DomainType** object defined in the enclosing **Model** object. (Reference: SBML Level 3 Specification for Spatial Processes, Version 1, Section 3.26 on page 29.)

**spatial-21907** ☑ The attribute `spatial:name` on an **AnalyticVolume** must have a value of data type `string`. (Reference: SBML Level 3 Specification for Spatial Processes, Version 1, Section 3.26 on page 29.)

**spatial-21908** ☑ The attribute `spatial:ordinal` on an **AnalyticVolume** must have a value of data type `integer`. (Reference: SBML Level 3 Specification for Spatial Processes, Version 1, Section 3.26 on page 29.)

**spatial-21950** ▲ No **AnalyticVolume** should have a `spatial:ordinal` attribute with the same value as a different **AnalyticVolume**. (Reference: SBML Level 3 Specification for Spatial Processes, Version 1, Section 3.26 on page 29.)

#### *Rules for ParametricGeometry object*

**spatial-22001** ☑ A **ParametricGeometry** object may have the optional SBML Level 3 Core attributes `metaid` and `sboTerm`. No other attributes from the SBML Level 3 Core namespaces are permitted on a **ParametricGeometry**. (Reference: SBML Level 3 Version 1 Core, Section 3.2.)

**spatial-22002** ☑ A **ParametricGeometry** object may have the optional SBML Level 3 Core subobjects for notes and annotations. No other elements from the SBML Level 3 Core namespaces are permitted on a **ParametricGeometry**. (Reference: SBML Level 3 Version 1 Core, Section 3.2.)

**spatial-22003** ☑ A **ParametricGeometry** object may contain one and only one instance of each of the **SpatialPoints** and **ListOfParametricObjects** elements. No other elements from the SBML Level 3 Spatial Processes namespaces are permitted on a **ParametricGeometry** object. (Reference: SBML Level 3 Specification for Spatial Processes, Version 1, Section 3.45 on page 40.)

**spatial-22004** ☑ Apart from the general notes and annotations subobjects permitted on all SBML objects, a **ListOfParametricObjects** container object may only contain **ParametricObject** objects. (Reference: SBML Level 3 Specification for Spatial Processes, Version 1, Section 3.45 on page 40.)

**spatial-22005** ☑ A **ListOfParametricObjects** object may have the optional SBML Level 3 Core attributes `metaid` and `sboTerm`. No other attributes from the SBML Level 3 Core namespaces are permitted on a **ListOfParametricObjects** object. (Reference: SBML Level 3 Specification for Spatial Processes, Version 1, Section 3.45 on page 40.)

**spatial-22050** ☑ A **Model** may not have a **ParametricGeometry** and a **Geometry** with exactly one **CoordinateComponent** child. (Reference: SBML Level 3 Specification for Spatial Processes, Version 1, Section 3.45 on page 40.)

#### *Rules for ParametricObject object*

**spatial-22101** ☑ A **ParametricObject** object may have the optional SBML Level 3 Core attributes `metaid` and `sboTerm`. No other attributes from the SBML Level 3 Core namespaces are permitted on a **ParametricObject**. (Reference: SBML Level 3 Version 1 Core, Section 3.2.)

**spatial-22102** ☑ A **ParametricObject** object may have the optional SBML Level 3 Core subobjects for notes and annotations. No other elements from the SBML Level 3 Core namespaces are permitted on a **ParametricObject**. (Reference: SBML Level 3 Version 1 Core, Section 3.2.)

**spatial-22103** ☑ A **ParametricObject** object must have the required attributes `spatial:id`, `spatial:polygonType`, `spatial:domainType`, `spatial:pointIndexLength` and `spatial:compression`, and may have the optional attributes spatial:name and spatial:dataType. No other attributes from the SBML Level 3 Spatial Processes namespaces are permitted on a **ParametricObject** object. (Reference: SBML Level 3 Specification for Spatial Processes, Version 1, Section 3.47 on page 42.)

**spatial-22104** ☑ The value of the attribute spatial:polygonType of a **ParametricObject** object must conform to the syntax of SBML data type PolygonKind and may only take on the allowed values of PolygonKind defined in SBML; that is, the value must be "triangle". (Reference: SBML Level 3 Specification for Spatial Processes, Version 1, Section 3.47 on page 42.)

**spatial-22105** ☑ The value of the attribute spatial:domainType of a **ParametricObject** object must be the identifier of an existing **DomainType** object defined in the enclosing **Model** object. (Reference: SBML Level 3 Specification for Spatial Processes, Version 1, Section 3.47 on page 42.)

**spatial-22106** ☑ The value of the text child of a **ParametricObject** object must be an array of values of type integer. (Reference: SBML Level 3 Specification for Spatial Processes, Version 1, Section 3.47 on page 42.)

**spatial-22107** ☑ The attribute spatial:pointIndexLength on a **ParametricObject** must have a value of data type integer. (Reference: SBML Level 3 Specification for Spatial Processes, Version 1, Section 3.47 on page 42.)

**spatial-22108** ☑ The value of the attribute spatial:compression of a **ParametricObject** object must conform to the syntax of SBML data type CompressionKind and may only take on the allowed values of CompressionKind defined in SBML; that is, the value must be one of the following: "uncompressed" or "deflated". (Reference: SBML Level 3 Specification for Spatial Processes, Version 1, Section 3.47 on page 42.)

**spatial-22109** ☑ The attribute spatial:name on a **ParametricObject** must have a value of data type string. (Reference: SBML Level 3 Specification for Spatial Processes, Version 1, Section 3.47 on page 42.)

**spatial-22110** ☑ The value of the attribute spatial:dataType of a **ParametricObject** object must conform to the syntax of SBML data type DataKind and may only take on a subset of the allowed values of DataKind defined in SBML; that is, the value must be one of the following: "uint", "uint8", "uint16" or "uint32". (Reference: SBML Level 3 Specification for Spatial Processes, Version 1, Section 3.47 on page 42.)

**spatial-22150** ☑ If the spatial:compression attribute of a **ParametricObject** has the value "uncompressed", the spatial:pointIndexLength attribute of that **ParametricObject** must equal the number of entries in the **ArrayData** child of the **ParametricObject**. (Reference: SBML Level 3 Specification for Spatial Processes, Version 1, Section 3.47 on page 42.)

**spatial-22151** ☑ If the spatial:compression attribute of a **ParametricObject** has the value "deflated", the spatial:pointIndexLength attribute of that **ParametricObject** must equal the number of entries of the **ArrayData** child of the **ParametricObject**. (Reference: SBML Level 3 Specification for Spatial Processes, Version 1, Section 3.47 on page 42.)

**spatial-22152** ☑ If the attribute spatial:polygonType of a **ParametricObject** has the value "triangle", the number of uncompressed entries in its **ArrayData** child must be evenly divisible by three. (Reference: SBML Level 3 Specification for Spatial Processes, Version 1, Section 3.47 on page 42.)

**spatial-22154** ☑ Every element of an uncompressed **ArrayData** child of a **ParametricObject** must be a non-negative integer. (Reference: SBML Level 3 Specification for Spatial Processes, Version 1, Section 3.47 on page 42.)

**spatial-22155** ☑ Every element of an uncompressed **ArrayData** child of a **ParametricObject** must be a value less than the number of points described by the **SpatialPoints** object in the same **ParametricGeometry**. (Reference: SBML Level 3 Specification for Spatial Processes, Version 1, Section 3.47 on page 42.)

**spatial-22156** ▲ Every face defined in the **ArrayData** child of a **ParametricObject** should be consistently defined in a clockwise or counter-clockwise fashion across all faces in the same **ParametricObject**. (Reference: SBML Level 3 Specification for Spatial Processes, Version 1, Section 3.47 on page 42.)

**spatial-22157** ☑ No two faces defined in the **ArrayData** child of a **ParametricObject** may share more than two coordinates. (Reference: SBML Level 3 Specification for Spatial Processes, Version 1, Section 3.47 on page 42.)

#### *Rules for CSGeometry object*

**spatial-22201** ☑ A **CSGeometry** object may have the optional SBML Level 3 Core attributes `metaid` and `sboTerm`. No other attributes from the SBML Level 3 Core namespaces are permitted on a **CSGeometry**. (Reference: SBML Level 3 Version 1 Core, Section 3.2.)

**spatial-22202** ☑ A **CSGeometry** object may have the optional SBML Level 3 Core subobjects for notes and annotations. No other elements from the SBML Level 3 Core namespaces are permitted on a **CSGeometry**. (Reference: SBML Level 3 Version 1 Core, Section 3.2.)

**spatial-22203** ☑ A **CSGeometry** object may contain one and only one instance of the **ListOfCSGObjects** element. No other elements from the SBML Level 3 Spatial Processes namespaces are permitted on a **CSGeometry** object. (Reference: SBML Level 3 Specification for Spatial Processes, Version 1, Section 3.32 on page 33.)

**spatial-22204** ☑ Apart from the general notes and annotations subobjects permitted on all SBML objects, a **ListOfCSGObjects** container object may only contain **CSGObject** objects. (Reference: SBML Level 3 Specification for Spatial Processes, Version 1, Section 3.32 on page 33.)

**spatial-22205** ☑ A **ListOfCSGObjects** object may have the optional SBML Level 3 Core attributes `metaid` and `sboTerm`. No other attributes from the SBML Level 3 Core namespaces are permitted on a **ListOfCSGObjects** object. (Reference: SBML Level 3 Specification for Spatial Processes, Version 1, Section 3.32 on page 33.)

#### *Rules for CSGObject object*

**spatial-22301** ☑ A **CSGObject** object may have the optional SBML Level 3 Core attributes `metaid` and `sboTerm`. No other attributes from the SBML Level 3 Core namespaces are permitted on a **CSGObject**. (Reference: SBML Level 3 Version 1 Core, Section 3.2.)

**spatial-22302** ☑ A **CSGObject** object may have the optional SBML Level 3 Core subobjects for notes and annotations. No other elements from the SBML Level 3 Core namespaces are permitted on a **CSGObject**. (Reference: SBML Level 3 Version 1 Core, Section 3.2.)

**spatial-22303** ☑ A **CSGObject** object must have the required attributes `spatial:id` and `spatial:domainType`, and may have the optional attributes `spatial:name` and `spatial:ordinal`. No other attributes from the SBML Level 3 Spatial Processes namespaces are permitted on a **CSGObject** object. (Reference: SBML Level 3 Specification for Spatial Processes, Version 1, Section 3.33 on page 33.)

**spatial-22304** ☑ A **CSGObject** object must contain one and only one instance of the ***CSGNode*** element. No other elements from the SBML Level 3 Spatial Processes namespaces are permitted on a

**CSGObject** object. (Reference: SBML Level 3 Specification for Spatial Processes, Version 1, Section 3.33 on page 33.)

**spatial-22305** ☑ The value of the attribute `spatial:domainType` of a **CSGObject** object must be the identifier of an existing **DomainType** object defined in the enclosing **Model** object. (Reference: SBML Level 3 Specification for Spatial Processes, Version 1, Section 3.33 on page 33.)

**spatial-22306** ☑ The attribute `spatial:name` on a **CSGObject** must have a value of data type `string`. (Reference: SBML Level 3 Specification for Spatial Processes, Version 1, Section 3.33 on page 33.)

**spatial-22307** ☑ The attribute `spatial:ordinal` on a **CSGObject** must have a value of data type `integer`. (Reference: SBML Level 3 Specification for Spatial Processes, Version 1, Section 3.33 on page 33.)

**spatial-22350** ▲ No **CSGObject** should have an `spatial:ordinal` attribute with the same value as a different **CSGObject**. (Reference: SBML Level 3 Specification for Spatial Processes, Version 1, Section 3.33 on page 33.)

**spatial-22351** ☑ Any **InteriorPoint** of the **DomainType** referenced by a **CSGObject** must be inside the geometry the **CSGObject** describes. (Reference: SBML Level 3 Specification for Spatial Processes, Version 1, Section 3.33 on page 33.)

#### *Rules for CSGNode object*

**spatial-22401** ☑ A ***CSGNode*** object may have the optional SBML Level 3 Core attributes `metaid` and `sboTerm`. No other attributes from the SBML Level 3 Core namespaces are permitted on a ***CSGNode***. (Reference: SBML Level 3 Version 1 Core, Section 3.2.)

**spatial-22402** ☑ A ***CSGNode*** object may have the optional SBML Level 3 Core subobjects for notes and annotations. No other elements from the SBML Level 3 Core namespaces are permitted on a ***CSGNode***. (Reference: SBML Level 3 Version 1 Core, Section 3.2.)

**spatial-22403** ☑ A ***CSGNode*** object may have the optional attributes `spatial:id` and `spatial:name`. No other attributes from the SBML Level 3 Spatial Processes namespaces are permitted on a ***CSGNode*** object. (Reference: SBML Level 3 Specification for Spatial Processes, Version 1, Section 3.34 on page 34.)

**spatial-22404** ☑ The attribute `spatial:name` on a ***CSGNode*** must have a value of data type `string`. (Reference: SBML Level 3 Specification for Spatial Processes, Version 1, Section 3.34 on page 34.)

#### *Rules for CSGTranslation object*

**spatial-22601** ☑ A **CSGTranslation** object may have the optional SBML Level 3 Core attributes `metaid` and `sboTerm`. No other attributes from the SBML Level 3 Core namespaces are permitted on a **CSGTranslation**. (Reference: SBML Level 3 Version 1 Core, Section 3.2.)

**spatial-22602** ☑ A **CSGTranslation** object may have the optional SBML Level 3 Core subobjects for notes and annotations. No other elements from the SBML Level 3 Core namespaces are permitted on a **CSGTranslation**. (Reference: SBML Level 3 Version 1 Core, Section 3.2.)

**spatial-22603** ☑ A **CSGTranslation** object must have the required attribute `spatial:translateX`, and may have the optional attributes `spatial:translateY` and `spatial:translateZ`. No other attributes from the SBML Level 3 Spatial Processes namespaces are permitted on a **CSGTranslation** object. (Reference: SBML Level 3 Specification for Spatial Processes, Version 1, Section 3.39 on page 37.)

**spatial-22604** ☑ The attribute `spatial:translateX` on a **CSGTranslation** must have a value of data type `double`. (Reference: SBML Level 3 Specification for Spatial Processes, Version 1, Section 3.39 on page 37.)

**spatial-22605** ☑ The attribute `spatial:translateY` on a **CSGTranslation** must have a value of data type `double`. (Reference: SBML Level 3 Specification for Spatial Processes, Version 1, Section 3.39 on page 37.)

**spatial-22606** ☑ The attribute `spatial:translateZ` on a **CSGTranslation** must have a value of data type `double`. (Reference: SBML Level 3 Specification for Spatial Processes, Version 1, Section 3.39 on page 37.)

**spatial-22650** ☑ A **CSGTranslation** object may contain one and only one child ***CSGNode*** element. No other elements from the SBML Level 3 Spatial Processes namespaces are permitted on a **CSGTranslation** object. (Reference: SBML Level 3 Specification for Spatial Processes, Version 1, Section 3.38 on page 36.)

**spatial-22651** ☑ The attribute `spatial:translateY` on a **CSGTranslation** is required if the **Geometry** of the **Model** has a **CoordinateComponent** child of type "`cartesianY`". (Reference: SBML Level 3 Specification for Spatial Processes, Version 1, Section 3.38 on page 36.)

**spatial-22652** ☑ The attribute `spatial:translateZ` on a **CSGTranslation** is required if the **Geometry** of the **Model** has a **CoordinateComponent** child of type "`cartesianZ`". (Reference: SBML Level 3 Specification for Spatial Processes, Version 1, Section 3.38 on page 36.)

**spatial-22653** ☑ The attribute `spatial:translateY` on a **CSGTranslation** must not be defined if the **Geometry** of the **Model** has no **CoordinateComponent** child of type "`cartesianY`". (Reference: SBML Level 3 Specification for Spatial Processes, Version 1, Section 3.38 on page 36.)

**spatial-22654** ☑ The attribute `spatial:translateZ` on a **CSGTranslation** must not be defined if the **Geometry** of the **Model** has no **CoordinateComponent** child of type "`cartesianZ`". (Reference: SBML Level 3 Specification for Spatial Processes, Version 1, Section 3.38 on page 36.)

#### *Rules for CSGRotation object*

**spatial-22701** ☑ A **CSGRotation** object may have the optional SBML Level 3 Core attributes `metaid` and `sboTerm`. No other attributes from the SBML Level 3 Core namespaces are permitted on a **CSGRotation**. (Reference: SBML Level 3 Version 1 Core, Section 3.2.)

**spatial-22702** ☑ A **CSGRotation** object may have the optional SBML Level 3 Core subobjects for notes and annotations. No other elements from the SBML Level 3 Core namespaces are permitted on a **CSGRotation**. (Reference: SBML Level 3 Version 1 Core, Section 3.2.)

**spatial-22703** ☑ A **CSGRotation** object must have the required attributes `spatial:rotateX` and `spatial:rotateAngleInRadians`, and may have the optional attributes `spatial:rotateY` and `spatial:rotateZ`. No other attributes from the SBML Level 3 Spatial Processes namespaces are permitted on a **CSGRotation** object. (Reference: SBML Level 3 Specification for Spatial Processes, Version 1, Section 3.40 on page 38.)

**spatial-22704** ☑ The attribute `spatial:rotateX` on a **CSGRotation** must have a value of data type `double`. (Reference: SBML Level 3 Specification for Spatial Processes, Version 1, Section 3.40 on page 38.)

**spatial-22705** ☑ The attribute `spatial:rotateAngleInRadians` on a **CSGRotation** must have a value of data type `double`. (Reference: SBML Level 3 Specification for Spatial Processes, Version 1, Section 3.40 on page 38.)

**spatial-22706** ☑ The attribute `spatial:rotateY` on a **CSGRotation** must have a value of data type `double`. (Reference: SBML Level 3 Specification for Spatial Processes, Version 1, Section 3.40 on page 38.)

**spatial-22707** ☑ The attribute `spatial:rotateZ` on a **CSGRotation** must have a value of data type `double`. (Reference: SBML Level 3 Specification for Spatial Processes, Version 1, Section 3.40 on page 38.)

**spatial-22750** ☑ A **CSGRotation** object may contain one and only one child ***CSGNode*** element. No other elements from the SBML Level 3 Spatial Processes namespaces are permitted on a **CSGRotation** object. (Reference: SBML Level 3 Specification for Spatial Processes, Version 1, Section 3.38 on page 36.)

**spatial-22751** ☑ The attribute `spatial:rotateY` on a **CSGRotation** is required if the **Geometry** of the **Model** has a **CoordinateComponent** child of type "`cartesianY`". (Reference: SBML Level 3 Specification for Spatial Processes, Version 1, Section 3.40 on page 38.)

**spatial-22752** ☑ The attribute `spatial:rotateZ` on a **CSGRotation** is required if the **Geometry** of the **Model** has a **CoordinateComponent** child of type "`cartesianZ`". (Reference: SBML Level 3 Specification for Spatial Processes, Version 1, Section 3.40 on page 38.)

**spatial-22753** ☑ The attribute `spatial:rotateY` on a **CSGRotation** must not be defined if the **Geometry** of the **Model** has no **CoordinateComponent** child of type "`cartesianY`". (Reference: SBML Level 3 Specification for Spatial Processes, Version 1, Section 3.40 on page 38.)

**spatial-22754** ☑ The attribute `spatial:rotateZ` on a **CSGRotation** must not be defined if the **Geometry** of the **Model** has no **CoordinateComponent** child of type "`cartesianZ`". (Reference: SBML Level 3 Specification for Spatial Processes, Version 1, Section 3.40 on page 38.)

**spatial-22755** ☑ If the **Geometry** of the **Model** has exactly three **CoordinateComponent** children, at least one of the attributes `spatial:rotateX`, `spatial:rotateY`, or `spatial:rotateZ` of a **CSGRotation** must not be equal to zero. (Reference: SBML Level 3 Specification for Spatial Processes, Version 1, Section 3.40 on page 38.)

#### *Rules for CSGScale object*

**spatial-22801** ☑ A **CSGScale** object may have the optional SBML Level 3 Core attributes `metaid` and `sboTerm`. No other attributes from the SBML Level 3 Core namespaces are permitted on a **CSGScale**. (Reference: SBML Level 3 Version 1 Core, Section 3.2.)

**spatial-22802** ☑ A **CSGScale** object may have the optional SBML Level 3 Core subobjects for notes and annotations. No other elements from the SBML Level 3 Core namespaces are permitted on a **CSGScale**. (Reference: SBML Level 3 Version 1 Core, Section 3.2.)

**spatial-22803** ☑ A **CSGScale** object must have the required attribute `spatial:scaleX`, and may have the optional attributes `spatial:scaleY` and `spatial:scaleZ`. No other attributes from the SBML Level 3 Spatial Processes namespaces are permitted on a **CSGScale** object. (Reference: SBML Level 3 Specification for Spatial Processes, Version 1, Section 3.41 on page 38.)

**spatial-22804** ☑ The attribute `spatial:scaleX` on a **CSGScale** must have a value of data type `double`. (Reference: SBML Level 3 Specification for Spatial Processes, Version 1, Section 3.41 on page 38.)

**spatial-22805** ☑ The attribute `spatial:scaleY` on a **CSGScale** must have a value of data type `double`. (Reference: SBML Level 3 Specification for Spatial Processes, Version 1, Section 3.41 on page 38.)

**spatial-22806** ☑ The attribute `spatial:scaleZ` on a **CSGScale** must have a value of data type `double`. (Reference: SBML Level 3 Specification for Spatial Processes, Version 1, Section 3.41 on page 38.)

**spatial-22850** ☑ A **CSGScale** object may contain one and only one child ***CSGNode*** element. No other elements from the SBML Level 3 Spatial Processes namespaces are permitted on a **CSGScale** object. (Reference: SBML Level 3 Specification for Spatial Processes, Version 1, Section 3.38 on page 36.)

**spatial-22851** ☑ The attribute `spatial:scaleY` on a **CSGScale** is required if the **Geometry** of the **Model** has a **CoordinateComponent** child of type "`cartesianY`". (Reference: SBML Level 3 Specification for Spatial Processes, Version 1, Section 3.41 on page 38.)

**spatial-22852** ☑ The attribute `spatial:scaleZ` on a **CSGScale** is required if the **Geometry** of the **Model** has a **CoordinateComponent** child of type "`cartesianZ`". (Reference: SBML Level 3 Specification for Spatial Processes, Version 1, Section 3.41 on page 38.)

**spatial-22853** ☑ The attribute `spatial:scaleY` on a **CSGScale** must not be defined if the **Geometry** of the **Model** has no **CoordinateComponent** child of type "`cartesianY`". (Reference: SBML Level 3 Specification for Spatial Processes, Version 1, Section 3.41 on page 38.)

**spatial-22854** ☑ The attribute `spatial:scaleZ` on a **CSGScale** must not be defined if the **Geometry** of the **Model** has no **CoordinateComponent** child of type "`cartesianZ`". (Reference: SBML Level 3 Specification for Spatial Processes, Version 1, Section 3.41 on page 38.)

#### *Rules for CSGHomogeneousTransformation object*

**spatial-22901** ☑ A **CSGHomogeneousTransformation** object may have the optional SBML Level 3 Core attributes `metaid` and `sboTerm`. No other attributes from the SBML Level 3 Core namespaces are permitted on a **CSGHomogeneousTransformation**. (Reference: SBML Level 3 Version 1 Core, Section 3.2.)

**spatial-22902** ☑ A **CSGHomogeneousTransformation** object may have the optional SBML Level 3 Core subobjects for notes and annotations. No other elements from the SBML Level 3 Core namespaces are permitted on a **CSGHomogeneousTransformation**. (Reference: SBML Level 3 Version 1 Core, Section 3.2.)

**spatial-22903** ☑ A **CSGHomogeneousTransformation** object must contain one and only one child `forwardTransformation` element of type **TransformationComponent**, and may additionally contain one and only one child ***CSGNode*** element. No other elements from the SBML Level 3 Spatial Processes namespaces are permitted on a **CSGHomogeneousTransformation** object. (Reference: SBML Level 3 Specification for Spatial Processes, Version 1, Section 3.42 on page 39.)

#### *Rules for TransformationComponent object*

**spatial-23001** ☑ A **TransformationComponent** object may have the optional SBML Level 3 Core attributes `metaid` and `sboTerm`. No other attributes from the SBML Level 3 Core namespaces are permitted on a **TransformationComponent**. (Reference: SBML Level 3 Version 1 Core, Section 3.2.)

**spatial-23002** ☑ A **TransformationComponent** object may have the optional SBML Level 3 Core subobjects for notes and annotations. No other elements from the SBML Level 3 Core namespaces are permitted on a **TransformationComponent**. (Reference: SBML Level 3 Version 1 Core, Section 3.2.)

**spatial-23003** ☑ A **TransformationComponent** object must have the required attributes `spatial:components` and `spatial:componentsLength`. No other attributes from the SBML Level 3 Spatial Processes namespaces are permitted on a **TransformationComponent** object. (Reference: SBML Level 3 Specification for Spatial Processes, Version 1, Section 3.43 on page 39.)

**spatial-23004** ☑ The value of the attribute `spatial:components` of a **TransformationComponent** object must be an array of values of type `double`. (Reference: SBML Level 3 Specification for Spatial Processes, Version 1, Section 3.43 on page 39.)

**spatial-23005** ☑ The attribute `spatial:componentsLength` on a **TransformationComponent** must have a value of data type `integer`. (Reference: SBML Level 3 Specification for Spatial Processes, Version 1, Section 3.43 on page 39.)

**spatial-23050** ☑ The attribute `spatial:componentsLength` on a **TransformationComponent** must have a value of "`16`". (Reference: SBML Level 3 Specification for Spatial Processes, Version 1, Section 3.43 on page 39.)

**spatial-23051** ☑ The length of the array of the attribute `spatial:components` on a **TransformationComponent** must be 16. (Reference: SBML Level 3 Specification for Spatial Processes, Version 1, Section 3.43 on page 39.)

#### *Rules for CSGPrimitive object*

**spatial-23101** ☑ A **CSGPrimitive** object may have the optional SBML Level 3 Core attributes `metaid` and `sboTerm`. No other attributes from the SBML Level 3 Core namespaces are permitted on a **CSGPrimitive**. (Reference: SBML Level 3 Version 1 Core, Section 3.2.)

**spatial-23102** ☑ A **CSGPrimitive** object may have the optional SBML Level 3 Core subobjects for notes and annotations. No other elements from the SBML Level 3 Core namespaces are permitted on a **CSGPrimitive**. (Reference: SBML Level 3 Version 1 Core, Section 3.2.)

**spatial-23103** ☑ A **CSGPrimitive** object must have the required attribute `spatial:primitiveType`. No other attributes from the SBML Level 3 Spatial Processes namespaces are permitted on a **CSGPrimitive** object. (Reference: SBML Level 3 Specification for Spatial Processes, Version 1, Section 3.35 on page 35.)

**spatial-23104** ☑ The value of the attribute `spatial:primitiveType` of a **CSGPrimitive** object must conform to the syntax of SBML data type `PrimitiveKind` and may only take on the allowed values of `PrimitiveKind` defined in SBML; that is, the value must be one of the following: "`sphere`", "`cube`", "`cylinder`", "`cone`", "`circle`" or "`square`". (Reference: SBML Level 3 Specification for Spatial Processes, Version 1, Section 3.35 on page 35.)

**spatial-23150** ☑ The `spatial:primitiveType` attribute of a **CSGPrimitive** may have the values "`sphere`", "`cube`", "`cylinder`", or "`cone`" only if the **Geometry** of the **Model** has exactly three **CoordinateComponent** children. (Reference: SBML Level 3 Specification for Spatial Processes, Version 1, Section 3.35 on page 35.)

**spatial-23151** ☑ The `spatial:primitiveType` attribute of a **CSGPrimitive** may have the values "`circle`" or "`square`" only if the **Geometry** of the **Model** has exactly two or three **CoordinateComponent** children. (Reference: SBML Level 3 Specification for Spatial Processes, Version 1, Section 3.35 on page 35.)

#### *Rules for CSGSetOperator object*

**spatial-23201** ☑ A **CSGSetOperator** object may have the optional SBML Level 3 Core attributes `metaid` and `sboTerm`. No other attributes from the SBML Level 3 Core namespaces are permitted on a **CSGSetOperator**. (Reference: SBML Level 3 Version 1 Core, Section 3.2.)

**spatial-23202** ☑ A **CSGSetOperator** object may have the optional SBML Level 3 Core subobjects for notes and annotations. No other elements from the SBML Level 3 Core namespaces are permitted on a **CSGSetOperator**. (Reference: SBML Level 3 Version 1 Core, Section 3.2.)

**spatial-23203** ☑ A **CSGSetOperator** object must have the required attribute `spatial:operationType`, and may have the optional attributes `spatial:complementA` and `spatial:complementB`. No other attributes from the SBML Level 3 Spatial Processes namespaces are permitted on a **CSGSetOperator** object. (Reference: SBML Level 3 Specification for Spatial Processes, Version 1, Section 3.36 on page 35.)

**spatial-23204** ☑ A **CSGSetOperator** object may contain one and only one instance of the **ListOfCSGNodes** element. No other elements from the SBML Level 3 Spatial Processes namespaces are permitted on a **CSGSetOperator** object. (Reference: SBML Level 3 Specification for Spatial Processes, Version 1, Section 3.36 on page 35.)

**spatial-23205** ☑ The value of the attribute `spatial:operationType` of a **CSGSetOperator** object must conform to the syntax of SBML data type `SetOperation` and may only take on the allowed values of `SetOperation` defined in SBML; that is, the value must be one of the following: "`union`", "`intersection`", or "`difference`". (Reference: SBML Level 3 Specification for Spatial Processes, Version 1, Section 3.36 on page 35.)

**spatial-23208** ☑ Apart from the general notes and annotations subobjects permitted on all SBML objects, a **ListOfCSGNodes** container object may only contain ***CSGNode*** objects. (Reference: SBML Level 3 Specification for Spatial Processes, Version 1, Section 3.37 on page 36.)

**spatial-23209** ☑ A **ListOfCSGNodes** object may have the optional SBML Level 3 Core attributes `metaid` and `sboTerm`. No other attributes from the SBML Level 3 Core namespaces are permitted on a **ListOfCSGNodes** object. (Reference: SBML Level 3 Specification for Spatial Processes, Version 1, Section 3.37 on page 36.)

**spatial-23250** ☑ If the `spatial:operationType` attribute of a **CSGSetOperator** has the value "`difference`", it must also define values for the attributes `spatial:complementA` and `spatial:complementB`. (Reference: SBML Level 3 Specification for Spatial Processes, Version 1, Section 3.36 on page 35.)

**spatial-23251** ☑ If the attribute `spatial:operationType` of a **CSGSetOperator** has the value "`union`" or "`intersection`", it must not define values for the attributes `spatial:complementA` nor `spatial:complementB`. (Reference: SBML Level 3 Specification for Spatial Processes, Version 1, Section 3.36 on page 35.)

**spatial-23252** ☑ If the attribute `spatial:operationType` of a **CSGSetOperator** is "`difference`", it must have exactly two ***CSGNode*** children. (Reference: SBML Level 3 Specification for Spatial Processes, Version 1, Section 3.36 on page 35.)

**spatial-23253** ☑ The attributes `spatial:complementA` and `spatial:complementB` of a **CSGSetOperator** must reference its two ***CSGNode*** children. (Reference: SBML Level 3 Specification for Spatial Processes, Version 1, Section 3.36 on page 35.)

**spatial-23254** ★ A **CSGSetOperator** should usually have at least two children, as operations involving zero or one child can be accomplished more efficiently in other ways. (Reference: SBML Level 3 Specification for Spatial Processes, Version 1, Section 3.36 on page 35.)

#### *Rules for SpatialSymbolReference object*

**spatial-23301** ☑ A **SpatialSymbolReference** object may have the optional SBML Level 3 Core attributes `metaid` and `sboTerm`. No other attributes from the SBML Level 3 Core namespaces are permitted on a **SpatialSymbolReference**. (Reference: SBML Level 3 Version 1 Core, Section 3.2.)

**spatial-23302** ☑ A **SpatialSymbolReference** object may have the optional SBML Level 3 Core subobjects for notes and annotations. No other elements from the SBML Level 3 Core namespaces are permitted on a **SpatialSymbolReference**. (Reference: SBML Level 3 Version 1 Core, Section 3.2.)

**spatial-23303** ☑ A **SpatialSymbolReference** object must have the required attribute `spatial:spatialRef`. No other attributes from the SBML Level 3 Spatial Processes namespaces are permitted on a **SpatialSymbolReference** object. (Reference: SBML Level 3 Specification for Spatial Processes, Version 1, Section 3.9 on page 14.)

**spatial-23304** ☑ The value of the attribute `spatial:spatialRef` of a **SpatialSymbolReference** object must be the identifier of an existing **CompartmentMapping**, **CoordinateComponent**, **Boundary**, **DomainType**, **Domain**, or **SampledField** object defined in the enclosing **Model** object. (Reference: SBML Level 3 Specification for Spatial Processes, Version 1, Section 3.9 on page 14.)

**spatial-23350** ☑ Every **SpatialSymbolReference** in a **Model** must reference a unique spatial element. (Reference: SBML Level 3 Specification for Spatial Processes, Version 1, Section 3.9 on page 14.)

#### *Rules for DiffusionCoefficient object*

**spatial-23401** ☑ A **DiffusionCoefficient** object may have the optional SBML Level 3 Core attributes `metaid` and `sboTerm`. No other attributes from the SBML Level 3 Core namespaces are permitted on a **DiffusionCoefficient**. (Reference: SBML Level 3 Version 1 Core, Section 3.2.)

**spatial-23402** ☑ A **DiffusionCoefficient** object may have the optional SBML Level 3 Core subobjects for notes and annotations. No other elements from the SBML Level 3 Core namespaces are permitted on a **DiffusionCoefficient**. (Reference: SBML Level 3 Version 1 Core, Section 3.2.)

**spatial-23403** ☑ A **DiffusionCoefficient** object must have the required attributes `spatial:variable` and `spatial:type`, and may have the optional attributes `spatial:coordinateReference1` and `spatial:coordinateReference2`. No other attributes from the SBML Level 3 Spatial Processes namespaces are permitted on a **DiffusionCoefficient** object. (Reference: SBML Level 3 Specification for Spatial Processes, Version 1, Section 3.10 on page 15.)

**spatial-23404** ☑ The value of the attribute `spatial:variable` of a **DiffusionCoefficient** object must be the identifier of an existing **Species** or **Parameter** object defined in the enclosing **Model** object. (Reference: SBML Level 3 Specification for Spatial Processes, Version 1, Section 3.10 on page 15.)

**spatial-23405** ☑ The value of the attribute `spatial:type` of a **DiffusionCoefficient** object must conform to the syntax of SBML data type `DiffusionKind` and may only take on the allowed values of `DiffusionKind` defined in SBML; that is, the value must be one of the following: "`isotropic`", "`anisotropic`" or "`tensor`". (Reference: SBML Level 3 Specification for Spatial Processes, Version 1, Section 3.10 on page 15.)

**spatial-23406** ☑ The value of the attribute `spatial:coordinateReference1` of a **DiffusionCoefficient** object must conform to the syntax of SBML data type `CoordinateKind` and may only take on the allowed values of `CoordinateKind` defined in SBML; that is, the value must be one of the following: "`cartesianX`", "`cartesianY`" or "`cartesianZ`". (Reference: SBML Level 3 Specification for Spatial Processes, Version 1, Section 3.10 on page 15.)

**spatial-23407** ☑ The value of the attribute `spatial:coordinateReference2` of a **DiffusionCoefficient** object must conform to the syntax of SBML data type `CoordinateKind` and may only take on the allowed values of `CoordinateKind` defined in SBML; that is, the value must be one of the following: "`cartesianX`", "`cartesianY`" or "`cartesianZ`". (Reference: SBML Level 3 Specification for Spatial Processes, Version 1, Section 3.10 on page 15.)

**spatial-23450** ☑ A **DiffusionCoefficient** with an attribute `spatial:type` value of "`isotropic`" may not define the attributes `spatial:coordinateReference1` nor `spatial:coordinateReference2`. (Reference: SBML Level 3 Specification for Spatial Processes, Version 1, Section 3.10 on page 15.)

**spatial-23451** ☑ A **DiffusionCoefficient** with an attribute `spatial:type` value of "`tensor`" must define the attributes `spatial:coordinateReference1` and `spatial:coordinateReference2`. (Reference: SBML Level 3 Specification for Spatial Processes, Version 1, Section 3.10 on page 15.)

**spatial-23452** ☑ A **DiffusionCoefficient** with an attribute `spatial:type` value of "`anisotropic`" must define the attribute `spatial:coordinateReference1`, and must not define the attribute `spatial:coordinateReference2`. (Reference: SBML Level 3 Specification for Spatial Processes, Version 1, Section 3.10 on page 15.)

**spatial-23453** ▲ The units of a **Parameter** with a **DiffusionCoefficient** child should be $length^2/time$. (Reference: SBML Level 3 Specification for Spatial Processes, Version 1, Section 3.10 on page 15.)

**spatial-23454** ☑ The **DiffusionCoefficient** attribute `spatial:coordinateReference2`, if defined, must have a value different from that of the attribute `spatial:coordinateReference1`. (Reference: SBML Level 3 Specification for Spatial Processes, Version 1, Section 3.10 on page 15.)

**spatial-23455** ☑ The **DiffusionCoefficient** attributes `spatial:coordinateReference1` and `spatial:coordinateReference2` may not have a value of "`cartesianY`" if the Geometry has exactly one CoordinateComponent child. (Reference: SBML Level 3 Specification for Spatial Processes, Version 1, Section 3.10 on page 15.)

**spatial-23456** ☑ The **DiffusionCoefficient** attributes `spatial:coordinateReference1` and `spatial:coordinateReference2` may not have a value of "`cartesianZ`" if the Geometry has exactly one or two CoordinateComponent children. (Reference: SBML Level 3 Specification for Spatial Processes, Version 1, Section 3.10 on page 15.)

**spatial-23457** ☑ Any **Species** or **Parameter** may only have a single **DiffusionCoefficient** that applies to any given cardinal axis or plane. A **DiffusionCoefficient** of type "`anisotropic`" applies to the axis it references, and any plane in the Geometry that contains that axis. A **DiffusionCoefficient** of type "`tensor`" applies to the plane defined by the two axes it references. A **DiffusionCoefficient** of type "`isotropic`" is considered to apply to all axes and planes in the **Geometry**. (Reference: SBML Level 3 Specification for Spatial Processes, Version 1, Section 3.10 on page 15.)

**spatial-23458** ☑ The value of the attribute `spatial:variable` of an **DiffusionCoefficient** object must not be the identifier of its parent **Parameter**. (Reference: SBML Level 3 Specification for Spatial Processes, Version 1, Section 3.11 on page 16.)

#### *Rules for AdvectionCoefficient object*

**spatial-23501** ☑ An **AdvectionCoefficient** object may have the optional SBML Level 3 Core attributes `metaid` and `sboTerm`. No other attributes from the SBML Level 3 Core namespaces are permitted on an **AdvectionCoefficient**. (Reference: SBML Level 3 Version 1 Core, Section 3.2.)

**spatial-23502** ☑ An **AdvectionCoefficient** object may have the optional SBML Level 3 Core subobjects for notes and annotations. No other elements from the SBML Level 3 Core namespaces are permitted on an **AdvectionCoefficient**. (Reference: SBML Level 3 Version 1 Core, Section 3.2.)

**spatial-23503** ☑ An **AdvectionCoefficient** object must have the required attributes `spatial:variable` and `spatial:coordinate`. No other attributes from the SBML Level 3 Spatial Processes namespaces are permitted on an **AdvectionCoefficient** object. (Reference: SBML Level 3 Specification for Spatial Processes, Version 1, Section 3.11 on page 16.)

**spatial-23504** ☑ The value of the attribute `spatial:variable` of an **AdvectionCoefficient** object must be the identifier of an existing **Species** or **Parameter** object defined in the enclosing **Model** object. (Reference: SBML Level 3 Specification for Spatial Processes, Version 1, Section 3.11 on page 16.)

**spatial-23505** ☑ The value of the attribute `spatial:coordinate` of an **AdvectionCoefficient** object must conform to the syntax of SBML data type `CoordinateKind` and may only take on the allowed values of `CoordinateKind` defined in SBML; that is, the value must be one of the following: "`cartesianX`", "`cartesianY`" or "`cartesianZ`". (Reference: SBML Level 3 Specification for Spatial Processes, Version 1, Section 3.11 on page 16.)

**spatial-23550** ▲ The units of a **Parameter** with an **AdvectionCoefficient** child should be $length/time$. (Reference: SBML Level 3 Specification for Spatial Processes, Version 1, Section 3.11 on page 16.)

**spatial-23551** ☑ No two **AdvectionCoefficient** elements in the same **Model** may have the same values for the attributes `spatial:variable` and `spatial:coordinate`. Only one advection coefficient may be defined per species (or parameter) per axis. (Reference: SBML Level 3 Specification for Spatial Processes, Version 1, Section 3.11 on page 16.)

**spatial-23552** ☑ The value of the attribute `spatial:variable` of an **AdvectionCoefficient** object must not be the identifier of its parent **Parameter**. (Reference: SBML Level 3 Specification for Spatial Processes, Version 1, Section 3.11 on page 16.)

#### *Rules for BoundaryCondition object*

**spatial-23601** ☑ A **BoundaryCondition** object may have the optional SBML Level 3 Core attributes `metaid` and `sboTerm`. No other attributes from the SBML Level 3 Core namespaces are permitted on a **BoundaryCondition**. (Reference: SBML Level 3 Version 1 Core, Section 3.2.)

**spatial-23602** ☑ A **BoundaryCondition** object may have the optional SBML Level 3 Core subobjects for notes and annotations. No other elements from the SBML Level 3 Core namespaces are permitted on a **BoundaryCondition**. (Reference: SBML Level 3 Version 1 Core, Section 3.2.)

**spatial-23603** ☑ A **BoundaryCondition** object must have the required attributes `spatial:variable` and `spatial:type`, and may have the optional attributes `spatial:coordinateBoundary` and `spatial:boundaryDomainType`. No other attributes from the SBML Level 3 Spatial Processes namespaces are permitted on a **BoundaryCondition** object. (Reference: SBML Level 3 Specification for Spatial Processes, Version 1, Section 3.12 on page 16.)

**spatial-23604** ☑ The value of the attribute `spatial:variable` of a **BoundaryCondition** object must be the identifier of an existing **Species** object defined in the enclosing **Model** object. (Reference: SBML Level 3 Specification for Spatial Processes, Version 1, Section 3.12 on page 16.)

**spatial-23605** ☑ The value of the attribute `spatial:type` of a **BoundaryCondition** object must conform to the syntax of SBML data type `BoundaryKind` and may only take on the allowed values of `BoundaryKind` defined in SBML; that is, the value must be one of the following: "`Neumann`" or "`Dirichlet`". (Reference: SBML Level 3 Specification for Spatial Processes, Version 1, Section 3.12 on page 16.)

**spatial-23606** ☑ The value of the attribute `spatial:coordinateBoundary` of a **BoundaryCondition** object must be the identifier of an existing **Boundary** object defined in the enclosing **Model** object. (Reference: SBML Level 3 Specification for Spatial Processes, Version 1, Section 3.12 on page 16.)

**spatial-23607** ☑ The value of the attribute `spatial:boundaryDomainType` of a **BoundaryCondition** object must be the identifier of an existing **DomainType** object defined in the enclosing **Model** object. (Reference: SBML Level 3 Specification for Spatial Processes, Version 1, Section 3.12 on page 16.)

**spatial-23650** ☑ A **BoundaryCondition** must define a value for either the attribute `spatial:coordinateBoundary` or `spatial:boundaryDomainType`, but not both. (Reference: SBML Level 3 Specification for Spatial Processes, Version 1, Section 3.12 on page 16.)

**spatial-23651** ☑ For every combination of species and boundary, there must be at most exactly one **BoundaryCondition** of type "Neumann", or exactly one **BoundaryCondition** of type "Dirichlet". (Reference: SBML Level 3 Specification for Spatial Processes, Version 1, Section 3.12 on page 16.)

**spatial-23652** ▲ The units of a **Parameter** with a **BoundaryCondition** child of type "Dirichlet" should be the units of concentration of the referenced **Species**. (Reference: SBML Level 3 Specification for Spatial Processes, Version 1, Section 3.12 on page 16.)

**spatial-23653** ▲ The units of a **Parameter** with a **BoundaryCondition** child of type "Neumann" should be the units of concentration of the referenced **Species**, times $length/time$. (Reference: SBML Level 3 Specification for Spatial Processes, Version 1, Section 3.12 on page 16.)

#### *Rules for Geometry object*

**spatial-23701** ☑ A **Geometry** object may have the optional SBML Level 3 Core attributes metaid and sboTerm. No other attributes from the SBML Level 3 Core namespaces are permitted on a **Geometry**. (Reference: SBML Level 3 Version 1 Core, Section 3.2.)

**spatial-23702** ☑ A **Geometry** object may have the optional SBML Level 3 Core subobjects for notes and annotations. No other elements from the SBML Level 3 Core namespaces are permitted on a **Geometry**. (Reference: SBML Level 3 Version 1 Core, Section 3.2.)

**spatial-23703** ☑ A **Geometry** object must have the required attribute spatial:coordinateSystem, and may have the optional attribute spatial:id. No other attributes from the SBML Level 3 Spatial Processes namespaces are permitted on a **Geometry** object. (Reference: SBML Level 3 Specification for Spatial Processes, Version 1, Section 3.17 on page 22.)

**spatial-23704** ☑ A **Geometry** object may contain one and only one instance of each of the **ListOfCoordinateComponents**, **ListOfDomainTypes**, **ListOfDomains**, **ListOfAdjacentDomains**, **ListOfGeometryDefinitions** and **ListOfSampledFields** elements. No other elements from the SBML Level 3 Spatial Processes namespaces are permitted on a **Geometry** object. (Reference: SBML Level 3 Specification for Spatial Processes, Version 1, Section 3.17 on page 22.)

**spatial-23705** ☑ The value of the attribute spatial:coordinateSystem of a **Geometry** object must conform to the syntax of SBML data type GeometryKind and may only take on the allowed value of GeometryKind defined in SBML; that is, the value must be "cartesian". (Reference: SBML Level 3 Specification for Spatial Processes, Version 1, Section 3.17 on page 22.)

**spatial-23706** ☑ Apart from the general notes and annotations subobjects permitted on all SBML objects, a **ListOfCoordinateComponents** container object may only contain **CoordinateComponent** objects. (Reference: SBML Level 3 Specification for Spatial Processes, Version 1, Section 3.17 on page 22.)

**spatial-23707** ☑ Apart from the general notes and annotations subobjects permitted on all SBML objects, a **ListOfDomainTypes** container object may only contain **DomainType** objects. (Reference: SBML Level 3 Specification for Spatial Processes, Version 1, Section 3.17 on page 22.)

**spatial-23708** ☑ Apart from the general notes and annotations subobjects permitted on all SBML objects, a **ListOfDomains** container object may only contain **Domain** objects. (Reference: SBML Level 3 Specification for Spatial Processes, Version 1, Section 3.17 on page 22.)

**spatial-23709** ☑ Apart from the general notes and annotations subobjects permitted on all SBML objects, a **ListOfAdjacentDomains** container object may only contain **AdjacentDomains** objects. (Reference: SBML Level 3 Specification for Spatial Processes, Version 1, Section 3.17 on page 22.)

**spatial-23710** ☑ Apart from the general notes and annotations subobjects permitted on all SBML objects, a **ListOfGeometryDefinitions** container object may only contain ***GeometryDefinition*** objects. (Reference: SBML Level 3 Specification for Spatial Processes, Version 1, Section 3.17 on page 22.)

**spatial-23711** ☑ Apart from the general notes and annotations subobjects permitted on all SBML objects, a **ListOfSampledFields** container object may only contain **SampledField** objects. (Reference: SBML Level 3 Specification for Spatial Processes, Version 1, Section 3.17 on page 22.)

**spatial-23712** ☑ A **ListOfCoordinateComponents** object may have the optional SBML Level 3 Core attributes `metaid` and `sboTerm`. No other attributes from the SBML Level 3 Core namespaces are permitted on a **ListOfCoordinateComponents** object. (Reference: SBML Level 3 Specification for Spatial Processes, Version 1, Section 3.17 on page 22.)

**spatial-23713** ☑ A **ListOfDomainTypes** object may have the optional SBML Level 3 Core attributes `metaid` and `sboTerm`. No other attributes from the SBML Level 3 Core namespaces are permitted on a **ListOfDomainTypes** object. (Reference: SBML Level 3 Specification for Spatial Processes, Version 1, Section 3.17 on page 22.)

**spatial-23714** ☑ A **ListOfDomains** object may have the optional SBML Level 3 Core attributes `metaid` and `sboTerm`. No other attributes from the SBML Level 3 Core namespaces are permitted on a **ListOfDomains** object. (Reference: SBML Level 3 Specification for Spatial Processes, Version 1, Section 3.17 on page 22.)

**spatial-23715** ☑ A **ListOfAdjacentDomains** object may have the optional SBML Level 3 Core attributes `metaid` and `sboTerm`. No other attributes from the SBML Level 3 Core namespaces are permitted on a **ListOfAdjacentDomains** object. (Reference: SBML Level 3 Specification for Spatial Processes, Version 1, Section 3.17 on page 22.)

**spatial-23716** ☑ A **ListOfGeometryDefinitions** object may have the optional SBML Level 3 Core attributes `metaid` and `sboTerm`. No other attributes from the SBML Level 3 Core namespaces are permitted on a **ListOfGeometryDefinitions** object. (Reference: SBML Level 3 Specification for Spatial Processes, Version 1, Section 3.17 on page 22.)

**spatial-23717** ☑ A **ListOfSampledFields** object may have the optional SBML Level 3 Core attributes `metaid` and `sboTerm`. No other attributes from the SBML Level 3 Core namespaces are permitted on a **ListOfSampledFields** object. (Reference: SBML Level 3 Specification for Spatial Processes, Version 1, Section 3.17 on page 22.)

**spatial-23750** ☑ A **Geometry** must have a child **ListOfCoordinateComponents** object. (Reference: SBML Level 3 Specification for Spatial Processes, Version 1, Section 3.17 on page 22.)

**spatial-23751** ☑ A **ListOfCoordinateComponents** must have exactly one, two, or three children. (Reference: SBML Level 3 Specification for Spatial Processes, Version 1, Section 3.17 on page 22.)

**spatial-23752** ☑ If a **ListOfCoordinateComponents** object has exactly one **CoordinateComponents** child, that child must have a `spatial:type` attribute with the value "`cartesianX`". (Reference: SBML Level 3 Specification for Spatial Processes, Version 1, Section 3.17 on page 22.)

**spatial-23753** ☑ If a **ListOfCoordinateComponents** object has exactly two **CoordinateComponents** children, one of them must have a `spatial:type` attribute with the value "`cartesianX`", and the other must have a `spatial:type` attribute with the value "`cartesianY`". (Reference: SBML Level 3 Specification for Spatial Processes, Version 1, Section 3.17 on page 22.)

**spatial-23754** ☑ If a **ListOfCoordinateComponents** object has exactly three **CoordinateComponents** children, one of them must have a `spatial:type` attribute with the value "`cartesianX`", one must have a spatial:type attribute with the value “cartesianY”, and one must have a spatial:type attribute with the value “cartesianZ”. (Reference: SBML Level 3 Specification for Spatial Processes, Version 1, Section 3.17 on page 22.)

#### Rules for MixedGeometry object

**spatial-23801** ☑ A MixedGeometry object may have the optional SBML Level 3 Core attributes metaid and sboTerm. No other attributes from the SBML Level 3 Core namespaces are permitted on a MixedGeometry. (Reference: SBML Level 3 Version 1 Core, Section 3.2.)

**spatial-23802** ☑ A MixedGeometry object may have the optional SBML Level 3 Core subobjects for notes and annotations. No other elements from the SBML Level 3 Core namespaces are permitted on a MixedGeometry. (Reference: SBML Level 3 Version 1 Core, Section 3.2.)

**spatial-23803** ☑ A MixedGeometry object may contain one and only one instance of each of the ListOfGeometryDefinitions and ListOfOrdinalMappings elements. No other elements from the SBML Level 3 Spatial Processes namespaces are permitted on a MixedGeometry object. (Reference: SBML Level 3 Specification for Spatial Processes, Version 1, Section 3.49 on page 44.)

**spatial-23804** ☑ Apart from the general notes and annotations subobjects permitted on all SBML objects, a ListOfGeometryDefinitions container object may only contain *GeometryDefinition* objects. (Reference: SBML Level 3 Specification for Spatial Processes, Version 1, Section 3.17 on page 22.)

**spatial-23805** ☑ Apart from the general notes and annotations subobjects permitted on all SBML objects, a ListOfOrdinalMappings container object may only contain OrdinalMapping objects. (Reference: SBML Level 3 Specification for Spatial Processes, Version 1, Section 3.49 on page 44.)

**spatial-23806** ☑ A ListOfGeometryDefinitions object may have the optional SBML Level 3 Core attributes metaid and sboTerm. No other attributes from the SBML Level 3 Core namespaces are permitted on a ListOfGeometryDefinitions object. (Reference: SBML Level 3 Specification for Spatial Processes, Version 1, Section 3.17 on page 22.)

**spatial-23807** ☑ A ListOfOrdinalMappings object may have the optional SBML Level 3 Core attributes metaid and sboTerm. No other attributes from the SBML Level 3 Core namespaces are permitted on a ListOfOrdinalMappings object. (Reference: SBML Level 3 Specification for Spatial Processes, Version 1, Section 3.49 on page 44.)

**spatial-23850** ☑ Every *GeometryDefinition* child of a MixedGeometry must have a spatial:isActive attribute value of “false”. (Reference: SBML Level 3 Specification for Spatial Processes, Version 1, Section 3.49 on page 44.)

#### Rules for OrdinalMapping object

**spatial-23901** ☑ An OrdinalMapping object may have the optional SBML Level 3 Core attributes metaid and sboTerm. No other attributes from the SBML Level 3 Core namespaces are permitted on an OrdinalMapping. (Reference: SBML Level 3 Version 1 Core, Section 3.2.)

**spatial-23902** ☑ An OrdinalMapping object may have the optional SBML Level 3 Core subobjects for notes and annotations. No other elements from the SBML Level 3 Core namespaces are permitted on an OrdinalMapping. (Reference: SBML Level 3 Version 1 Core, Section 3.2.)

**spatial-23903** ☑ An OrdinalMapping object must have the required attributes spatial:geometryDefinition and spatial:ordinal. No other attributes from the SBML Level 3 Spatial Processes namespaces are permitted on an OrdinalMapping object. (Reference: SBML Level 3 Specification for Spatial Processes, Version 1, Section 3.50 on page 45.)

**spatial-23904** ☑ The value of the attribute `spatial:geometryDefinition` of an **OrdinalMapping** object must be the identifier of an existing ***GeometryDefinition*** object defined in the parent **MixedGeometry** object. (Reference: SBML Level 3 Specification for Spatial Processes, Version 1, Section 3.50 on page 45.)

**spatial-23905** ☑ The attribute `spatial:ordinal` on an **OrdinalMapping** must have a value of data type `integer`. (Reference: SBML Level 3 Specification for Spatial Processes, Version 1, Section 3.50 on page 45.)

**spatial-23950** ▲ No **OrdinalMapping** should have a `spatial:ordinal` attribute with the same value as a different **OrdinalMapping** child of the same **MixedGeometry**. (Reference: SBML Level 3 Specification for Spatial Processes, Version 1, Section 3.50 on page 45.)

#### *Rules for SpatialPoints object*

**spatial-24001** ☑ A **SpatialPoints** object may have the optional SBML Level 3 Core attributes `metaid` and `sboTerm`. No other attributes from the SBML Level 3 Core namespaces are permitted on a **SpatialPoints**. (Reference: SBML Level 3 Version 1 Core, Section 3.2.)

**spatial-24002** ☑ A **SpatialPoints** object may have the optional SBML Level 3 Core subobjects for notes and annotations. No other elements from the SBML Level 3 Core namespaces are permitted on a **SpatialPoints**. (Reference: SBML Level 3 Version 1 Core, Section 3.2.)

**spatial-24003** ☑ A **SpatialPoints** object must have the required attributes `spatial:compression` and `spatial:arrayDataLength`, and may have the optional attributes `spatial:id`, `spatial:name` and `spatial:dataType`. No other attributes from the SBML Level 3 Spatial Processes namespaces are permitted on a **SpatialPoints** object. (Reference: SBML Level 3 Specification for Spatial Processes, Version 1, Section 3.46 on page 40.)

**spatial-24004** ☑ The value of the attribute `spatial:compression` of a **SpatialPoints** object must conform to the syntax of SBML data type `CompressionKind` and may only take on the allowed values of `CompressionKind` defined in SBML; that is, the value must be one of the following: "`uncompressed`" or "`deflated`". (Reference: SBML Level 3 Specification for Spatial Processes, Version 1, Section 3.46 on page 40.)

**spatial-24005** ☑ The value of the text child of a **SpatialPoints** object must be an array of values of type `double`. (Reference: SBML Level 3 Specification for Spatial Processes, Version 1, Section 3.46 on page 40.)

**spatial-24006** ☑ The attribute `spatial:arrayDataLength` on a **SpatialPoints** must have a value of data type `integer`. (Reference: SBML Level 3 Specification for Spatial Processes, Version 1, Section 3.46 on page 40.)

**spatial-24007** ☑ The attribute `spatial:name` on a **SpatialPoints** must have a value of data type `string`. (Reference: SBML Level 3 Specification for Spatial Processes, Version 1, Section 3.46 on page 40.)

**spatial-24008** ☑ The value of the attribute `spatial:dataType` of a **SpatialPoints** object must conform to the syntax of SBML data type `DataKind` and may only take on the allowed values of `DataKind` defined in SBML; that is, the value must be one of the following: "`double`", "`float`", "`int`", "`uint`", "`uint8`", "`uint16`" or "`uint32`". (Reference: SBML Level 3 Specification for Spatial Processes, Version 1, Section 3.46 on page 40.)

**spatial-24050** ☑ If the `spatial:compression` attribute of a **SpatialPoints** has the value "`uncompressed`", the `spatial:arrayDataLength` attribute of that **SpatialPoints** must equal the number of entries in the **ArrayData** child of the **SpatialPoints**. (Reference: SBML Level 3 Specification for Spatial Processes, Version 1, Section 3.46 on page 40.)

**spatial-24051** ☑ If the `spatial:compression` attribute of a **SpatialPoints** has the value "`deflated`", the `spatial:arrayDataLength` attribute of that **SpatialPoints** must equal the number of entries of the **ArrayData** child of the **SpatialPoints**. (Reference: SBML Level 3 Specification for Spatial Processes, Version 1, Section 3.46 on page 40.)

**spatial-24052** ☑ When uncompressed, the number of entries in the **ArrayData** child of a **SpatialPoints** must be evenly divisible by the number of **CoordinateComponent** children of the **Geometry** of the **Model** (two or three). (Reference: SBML Level 3 Specification for Spatial Processes, Version 1, Section 3.46 on page 40.)

**spatial-24053** ☑ If the `spatial:dataType` attribute of a **SpatialPoints** has the value "`float`", none of the uncompressed entries in the **ArrayData** child of the **SpatialPoints** may have a value outside of the range of an IEEE 754-1985 single-precision floating point value (approximately +/- 3.4028235e38, and +/-1.17549e-38). (Reference: SBML Level 3 Specification for Spatial Processes, Version 1, Section 3.46 on page 40.)

**spatial-24054** ☑ If the `spatial:dataType` attribute of a **SpatialPoints** has the value "`uint`", none of the uncompressed entries in the **ArrayData** child of the **SpatialPoints** may be negative. (Reference: SBML Level 3 Specification for Spatial Processes, Version 1, Section 3.46 on page 40.)

**spatial-24055** ☑ If the `spatial:dataType` attribute of a **SpatialPoints** has the value "`uint`" or "`int`", all of the uncompressed entries in the **ArrayData** child of the **SpatialPoints** must be integers. (Reference: SBML Level 3 Specification for Spatial Processes, Version 1, Section 3.46 on page 40.)

**spatial-24056** ☑ The value of the children of a **SpatialPoints** object must be an array of values of type `integer` if the "`spatial:compression`" attribute has the value "`deflated`". (Reference: SBML Level 3 Specification for Spatial Processes, Version 1, Section 3.46 on page 40.)

## Acknowledgments

Work such as this does not take place in a vacuum; many people contributed ideas and discussions that shaped the Groups proposal that you see before you. We particularly thank Bhavye Jain for working on adding validation rules, and the members of the *sbml-discuss* and *sbml-spatial* mailing lists for suggestions and comments.

This work was partially funded by a variety of sources including:

- The German Federal Ministry of Education and Research within de.NBI (031L0104A) (FTB)
- The National Institutes of Health grants P41 GM103712 (RFM), R01 GM090033 (RFM), R24 GM137787 (JCS, AL), and P41 GM109824 (LPS)
- The National Science Foundation award 1933453 (LPS)
- JSPS KAKENHI, grant numbers JP21700328 and JP24300112 (AF)
